# Comprehensive Evaluation of Biological Effects of Pentathiepins on Various Human Cancer Cell Lines and Insights into Their Mode of Action

**DOI:** 10.3390/ijms22147631

**Published:** 2021-07-16

**Authors:** Lisa Wolff, Siva Sankar Murthy Bandaru, Elias Eger, Hoai-Nhi Lam, Martin Napierkowski, Daniel Baecker, Carola Schulzke, Patrick J. Bednarski

**Affiliations:** 1 Pharmazeutische/Medizinische Chemie, Institut für Pharmazie, Universität Greifswald, 17489 Greifswald, Germany; lisa.wolff@uni-greifswald.de (L.W.); hoai-nhi.lam@uni-greifswald.de (H.-N.L.); martin.napierkowski@uni-greifswald.de (M.N.); daniel.baecker@uni-greifswald.de (D.B.); 2 Bioanorganische Chemie, Institut für Biochemie, Universität Greifswald, 17489 Greifswald, Germany; siva.bandaru@uni-greifswald.de; 3 Pharmazeutische Mikrobiologie, Institut für Pharmazie, Universität Greifswald, 17489 Greifswald, Germany; elias.eger@uni-greifswald.de

**Keywords:** cancer cells, cytotoxicity, DNA damage, glutathione peroxidase, pentathiepin, reactive oxygen species, sulfur

## Abstract

Pentathiepins are polysulfur-containing compounds that exert antiproliferative and cytotoxic activity in cancer cells, induce oxidative stress and apoptosis, and inhibit glutathione peroxidase (GPx1). This renders them promising candidates for anticancer drug development. However, the biological effects and how they intertwine have not yet been systematically assessed in diverse cancer cell lines. In this study, six novel pentathiepins were synthesized to suit particular requirements such as fluorescent properties or improved water solubility. Structural elucidation by X-ray crystallography was successful for three derivatives. All six underwent extensive biological evaluation in 14 human cancer cell lines. These studies included investigating the inhibition of GPx1 and cell proliferation, cytotoxicity, and the induction of ROS and DNA strand breaks. Furthermore, selected hallmarks of apoptosis and the impact on cell cycle progression were studied. All six pentathiepins exerted high cytotoxic and antiproliferative activity, while five also strongly inhibited GPx1. There is a clear connection between the potential to provoke oxidative stress and damage to DNA in the form of single- and double-strand breaks. Additionally, these studies support apoptosis but not ferroptosis as the mechanism of cell death in some of the cell lines. As the various pentathiepins give rise to different biological responses, modulation of the biological effects depends on the distinct chemical structures fused to the sulfur ring. This may allow for an optimization of the anticancer activity of pentathiepins in the future.

## 1. Introduction

**Figure 1:**
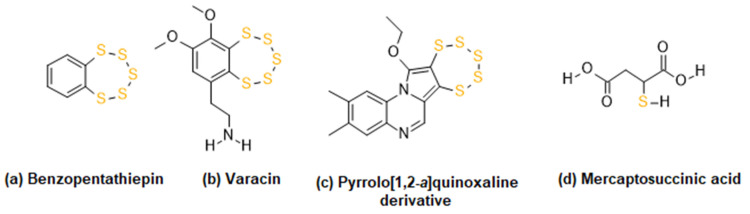
Chemical structures of (**a**) benzopentathiepin as one of the first synthesized pentathiepins; (**b**) varacin as the first isolated natural pentathiepin; (**c**) a pyrrolo[1,2-*a*]quinoxaline-based pentathiepin from our previous study; and (**d**) mercaptosuccinic acid, an inhibitor of the GPx1.

Pentathiepins are a class of compounds characterized by a seven-membered ring consisting of five sulfur and two carbon atoms [1]. First synthetic representatives, including a simple benzopentathiepin (Figure 1a), were already described in 1971 but not investigated in detail regarding possible biological activities [2]. This was first conducted in the early 1990s, when the natural pentathiepin varacin (Figure 1b) was isolated from marine Ascidiacea and shown to possess potent cytotoxic activity [3]. Thereafter, intensified research attributed interesting biological effects to this striking class of compounds, such as antifungal, antiviral, antibacterial, and DNA-cleaving activity, as well as cytotoxicity in cancer cell lines [3,4,5,6,7]. Furthermore, pentathiepins were reported to be specific inhibitors of protein kinase C (PKC), the striatal-enriched protein tyrosine phosphatase (STEP), which is implicated in Alzheimer’s disease, or the tyrosyl-DNA phosphodiesterase 1 (TDP1), which contributes to DNA repair in various cancerous and neurodegenerative disorders [8,9,10,11]. Recently, we reported on several newly synthesized pentathiepins on the basis of the pyrrolo[1,2-*a*]quinoxaline structure (Figure 1c) as potent and specific inhibitors of the glutathione peroxidase 1 (GPx1), adding another unexpected biological property to this promising class of compounds [12].

The GPx1 constitutes one of eight human isoforms of glutathione peroxidases and one of the five that contain a selenocysteine in their catalytic center [13,14]. The most prevalent isoforms are the cytoplasmic GPx1 and the membrane-bound GPx4, which maintain the physiological intracellular redox state and protect the membrane from lipid peroxidation, respectively. The latter is a key player in ferroptosis, an iron-dependent modality of cell death that can be induced by inactivation of GPx4 [15,16]. GPx1 is a key enzyme in cellular redox regulation; its role in cancer initiation, progression, and treatment has been diversely discussed in the literature. In several publications, a high GPx1 expression correlated with a poor outcome prediction [17,18], while others detected decreased GPx1 level in cancerous tissue compared with healthy tissue, thus ascribing the GPx1 a protective effect [19,20]. However, chemotherapeutic resistance mechanisms have been linked to increased expression of GPx1 [21]. Moreover, in a recent publication, GPx1 knockout cells proved to be significantly more sensitive to treatment with the chemotherapeutics cisplatin, lomustine, and temozolomide compared with the parental cell line [22]. Hence, therapeutic inhibition of the GPx1 may be a promising approach for potentiating cancer chemotherapy in drug-resistant cells, one which could be pursued by using pentathiepins.

With this strategy in mind, we have examined novel pentathiepins not only with regards to their potential to inhibit the GPx1 but also in the context of cytotoxicity in cancer cells [12]. The five pyrrolo[1,2-*a*]quinoxaline and three indole-based pentathiepins studied in our previous paper exceeded the GPx1 inhibition potential of the thus far best characterized GPx1 inhibitor, mercaptosuccinic acid (MSA, Figure 1d) [23]. Among the published series of pentathiepins, one representative was selected and further investigated regarding its biological effects (Figure 1). One key finding was the increase of intracellular reactive oxygen species (ROS) levels up to fourfold compared with a solvent-treated control. This was in accordance with earlier studies that postulated a reaction of the polysulfur ring system with thiols, thereby forming H_2_O_2_ via polysulfide anion intermediates [24]. In addition, the accumulation of ROS may result from the decreased capacity of the GPx1 enzyme; GPx1 actually detoxifies peroxides (e.g., H_2_O_2_) but was inhibited by the pentathiepins. Overexposure of cancer cells to intracellular ROS, exceeding the physiological equilibrium, can have a number of dire consequences for cells. This includes the damage of DNA via strand breaks and the induction of apoptosis as well as interference with normal mitochondrial function, as it was recently confirmed by Behnisch-Cornwell et al. after treating cells with a GPx1-inhibiting pentathiepin [12].

The currently available data suggest a broad spectrum of biological effects mediated by pentathiepins that potentially contribute to their anti-cancer activity. Not only the inhibition of the GPx1 renders these polysulfur structures an interesting class of compounds; their great capability to prevent the proliferation of cancer cells and the induction of apoptosis as a controlled form of cell death labels them as potentially useful anti-tumor treatments. Still, no attempts have been made as of yet to explore possible structure-activity relationships (SARs), and comparisons of various biological effects over several cell lines are also lacking. Furthermore, the inhibition of GPx1 and the cytotoxicity caused by the pentathiepins have not been directly correlated to date.

**Figure 2:**
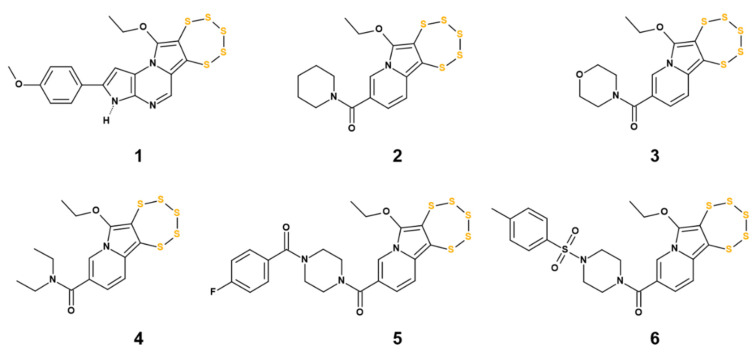
The chemical structures of the novel pentathiepins **1**–**6** that were synthesized and biologically evaluated in this study. Compound **1** contains an aryl-substituted pyrrolo-pyrazine designed to exert fluorescence for intracellular trackability. For the other structures, a nicotinamide backbone was substituted with either a piperidine (**2**), morpholine (**3**), *N*,*N’*-diethylamine (**4**), *p*-fluorophenone-piperazine (**5**), or *p*-tosyl-piperazine (**6**).

The present publication describes the synthesis of six additional pentathiepins (Figure 2) and their comprehensive biological evaluation. Pentathiepin **1** was synthesized and fused to a pyrrolo-pyrazine scaffold with the objective to produce a fluorescent compound that would facilitate the analysis of intracellular distribution; pyrrolo-annelated N-heterocycles are known cores of chromophores with fluorescent properties [25]. Five of the new compounds (**2**–**6**) possess a nicotinamide backbone, a structure that potentially increases water solubility and is well known for its biological activity [26]. This scaffold is substituted with either a piperidine (**2**), morpholine (**3**), *N*,*N*-diethylamine (**4**), p-fluorophenone-piperazine (**5**), or p-tosyl-piperazine (**6**). Piperazines in particular are frequently used in the rational design of therapeutic agents [27]. Additionally, the introduction of fluorine is widely applied in pharmaceutical medicinal chemistry. It can increase metabolic stability but also enhance binding affinities to a protein target or serve as tracer for ^19^F-NMR spectroscopy studies [28,29,30].

**Figure 3:**
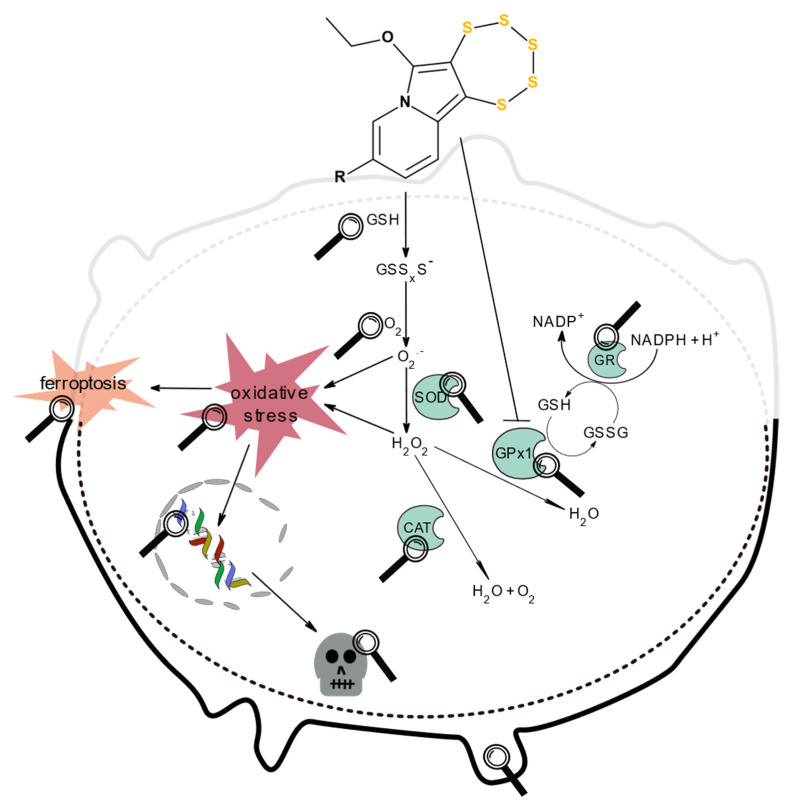
The mechanism of action exerted by pentathiepins in terms of the postulations of previous studies and adapted in order to clarify the objectives for the current comprehensive biological evaluations [12,24]. Magnifying glasses highlight the subjects of the present study.

From a biological perspective, we present a more detailed adaptation of the postulated mechanism of action (Figure 3). First, we assessed the potential of the compounds to inhibit the isolated GPx1 and then screened for cytotoxicity across a panel of 14 human cancer cell lines under normoxic and hypoxic conditions. In selected cell lines, the induction of oxidative stress was analyzed, and subsequently the DNA-damaging potential was studied. Particular emphasis was directed towards the cell death mechanism, with experiments covering the induction of apoptosis or ferroptosis. Moreover, we evaluated how the availability of oxygen and glutathione affects the action of pentathiepins, as well as whether the novel compounds inhibit other antioxidant enzymes such as catalase and glutathione reductase. These investigations shed light on the cellular effects of pentathiepins and in what manner oxidative stress, DNA damage, and apoptosis, as well as cell cycle aberrations, intertwine and contribute to the high cytotoxicity of these compounds.

## 2. Results and Discussion

### 2.1. Chemistry

#### 2.1.1. Synthesis of 11-methoxy-2-(4-methoxyphenyl)-3H-[1,2,3,4,5] pentathiepino[6′,7′:3,4]pyrrolo[1,2-a]pyrrolo[2,3-e]pyrazine (**1**)

**Scheme 1:**
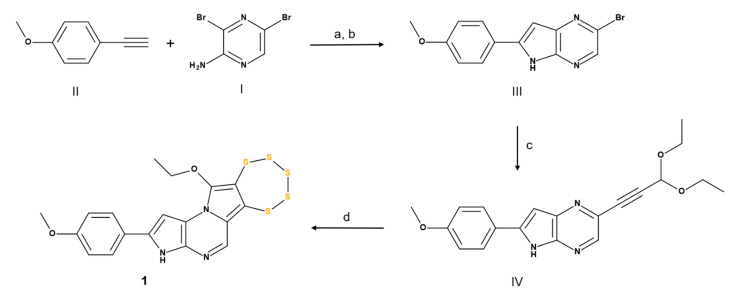
Synthesis of 11-methoxy-2-(4-methoxy < phenyl)-3*H*-[1,2,3,4,5]pentathiepino[6′,7′:3,4]pyrrolo[1,2-*a*]pyrrolo[2,3-*e*]pyrazine: (**a**) Pd(OAc)_2_ 5 mol %, PPh_3_ 7 mol %, 6 mol % CuI, Et_3_N, 3 equiv., CH_3_CN, 80 °C, overnight; (**b**) NaH, dry THF, 60 °C, 18 h, 78% (two steps); (**c**) Pd(PPh_3_)_2_Cl_2_ 5 mol %, 1.2 equiv., 3,3′-diethoxypropyne, 3 mol % CuI, Et_3_N 3 equiv., DMF, rt, overnight; (**d**) (Et_4_N)_2_ [MoO(S_4_)_2_] 0.5 equiv., S_8_ 1 equiv., DMF, 50 °C, 15 h.

Compound **1** was synthesized from 2,6-dibromo-3-amino-pyrazine (**I**) via two sequential Sonogashira cross-coupling steps. Initially, the cross-coupling of compound (**I**) with alkyne synthon (**II**) in the Pd(II)/Cu(I) catalytic system resulted in the Sonogashira product. The resultant crude reaction mixture was utilized in the ring-closing step in the presence of sodium hydride to form the pyrrolo-pyrazine unit (**III**). Compound **III** was isolated in 78% yield after column purification and exhibited fluorescence on the TLC plate under a UV lamp at λ = 356 nm irradiation. It was then coupled with 3,3′-diethoxy-propyne under Sonogashira reaction conditions, with subsequent ring-closing by the molybdenum complex, resulting in the targeted fluorescent pentathiepin derivative **1** as a red solid in 18% isolated yield after column chromatography purification (35% EtOAc/hexane) (Scheme 1). The purified sample was characterized by ^1^H-NMR, APCI-MS, and elemental analysis (Supplementary Materials).

#### 2.1.2. Synthesis of Nicotinamide-Fused Pentathiepins (**2**–**6**)

**Scheme 2:**
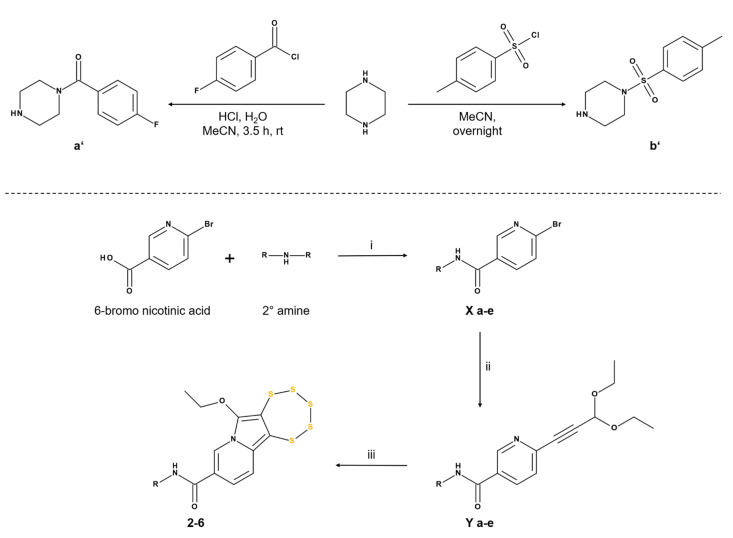
(**i**) HBTU, DIPEA, 0 °C to rt, 18 h; (**ii**) Pd (PPh_3_)_2_Cl_2_ 5 mol %, 3,3′-diethoxypropyne, CuI 5 mol %, 10 equiv. Et_3_N, DMF, 18 h, rt; (**iii**) (Et_4_N)_2_ [MoO(S_2_)_4_] 0.5 equiv., 1 equiv. S_8_, DMF, 15 h, rt; **2** (a, R = piperidine), **3** (b, R = morpholine), **4** (c, R = *N,N’*-diethyl), **5** (d, R = a’), and **6** (e, R = b’).

**Figure 4:**
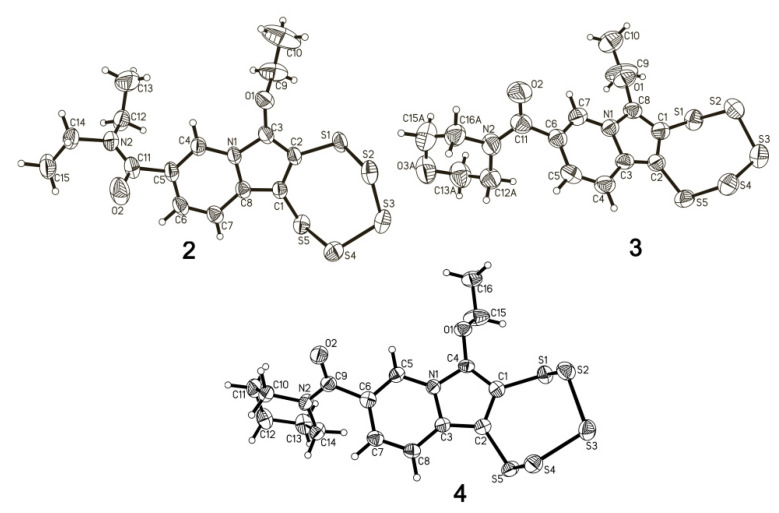
The crystal structures of compounds **2**–**4** shown with ellipsoids at the 50% level. Minor occupancy disorder atoms of the morpholine ring in **3** were omitted for clarity reasons.

The respective nicotinamide derivatives were synthesized on the basis of a procedure described in the literature [31]. Piperazine was protected either with benzoyl or sulfonyl chloride, corresponding to secondary amine precursors “a’” and “b’”, which were used in the following reactions (Scheme 2). Subsequently, 6-bromo nicotinic acid was allowed to react with secondary amines piperidine, morpholine, *N,N’*-diethylamine, and protected piperazines (a’ and b’) in the presence of 3-[bis(dimethylamino)methyliumyl]-3*H*-benzotriazole-1-oxide hexafluorophosphate (HBTU) and the base diisopropylethylamine (DIPEA) at low temperatures to yield the respective amide derivatives **Xa**–**e.** These nicotinamide derivatives were further subjected to sequential reactions of the Pd(II)-catalyzed Sonogashira cross-coupling, giving **Ya**–**e**. The following Mo^IV^-mediated ring-closing steps resulted in the desired nicotinamide-fused pentathiepins **2**–**6** in good yields (Scheme 2). The final structures of the compounds were confirmed by ^1^H, ^13^C, ^19^F-NMR (^19^F where applicable), APCI-MS, and elemental analysis (Supplementary Materials). The molecular structures of compounds **2**, **3**, and **4** were further verified by single-crystal X-ray diffraction analysis (Figure 4).

### 2.2. Biology

#### 2.2.1. Inhibition of Glutathione Peroxidase **1**

**Figure 5:**
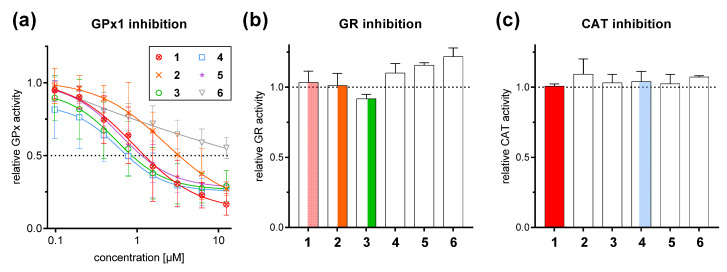
(**a**) Inhibition of bovine erythrocyte GPx1 enzyme activity as a function of pentathiepin concentration at 25 °C, displayed as means with the corresponding 95% confidence intervals (CI 95%). (**b**) Relative activity of glutathione reductase (GR) after treatment with 25 µM of pentathiepins **1**–**6**. (**c**) Relative activity of catalase (CAT) after treatment with 25 µM of pentathiepins **1**–**6**. The data represent the mean and SD or CI 95% of n ≥ 3 independent experiments.

The inhibition of GPx1 by pentathiepins has already been reported by us previously [12]. To investigate whether the new compounds **1**–**6** can also inhibit GPx1, we performed an enzymatic assay with *tert*-butylhydroperoxide (*t*-BHP) as substrate, which is reduced by bovine erythrocyte GPx1 under consumption of glutathione (GSH). The resulting glutathione disulfide (GSSG) was reduced back to GSH by glutathione reductase (GR), thereby converting NADPH to NADP^+^, which can be photometrically monitored at λ = 340 nm. The rate of NADPH consumption was used to quantify GPx1 enzyme activity. Dose-response graphs are presented in Figure 5a, and the corresponding IC_50_ values in Table 1. Relative IC_50_ describing the half-maximal inhibition of the enzyme were derived from the inflection point of the sigmoidal shaped graphs. Absolute IC_50_ was calculated via interpolation of the standard curve resulting in the concentration at which 50% of the GPx1 was inhibited.

Four out of six pentathiepins, namely **1**, **3**, **4**, and **5**, had a strong inhibitory effect on the bovine erythrocyte GPx with IC_50_ values between 0.56 and 0.92 µM (Table 1). However, residual enzyme activities at the highest tested concentration (12.5 µM) ranging from about 16 to roughly 30% were apparent. Compound **2** had an IC_50_ of 2.28 µM and a residual activity similar to that of **3**, **4**, and **5**, while **6** was unable to decrease the activity of the bovine GPx by more than 50% at 12.5 µM. In the GPx1 inhibition assay, five of the six novel pentathiepins had IC_50_ values between 0.6 and 2.3 µM. Hence, compounds **1**–**5** were considerably more potent than the thus far best characterized inhibitor mercaptosuccinic acid (IC_50_ of 5.9 µM) and just as effective as our recently published pentathiepins [12]. A morpholino (**3**) or *N*,*N’*-diethylamine (**4**) moiety fused to a nicotinamide backbone is considerably more potent than a piperidine (**2**). The introduction of a p-fluorobenzoylpiperazine (**5**) to the piperazine–nicotinamide structure instead of a p-tosyl residue (**6**) drastically increased the inhibitory activity, rendering it similarly effective as **3** and **4**. Pentathiepin **1**, originally designed to serve as a trackable compound, inhibited the GPx1 with an IC_50_ of about 0.9 µM but with a much lower residual activity at the highest tested concentration (16% vs. 30% for compounds **2**–**5** at 12.5 µM), demonstrating that a pyrrolo-pyrazine-based pentathiepin can also serve as potent inhibitor of the enzyme.

To assess the specificity of inhibitors toward the GPx1, we performed enzymatic assays with other relevant antioxidant enzymes. These included the glutathione reductase (GR; yeast-derived), which is an auxiliary component of the GPx1 assay, and catalase (CAT; bovine), a key redox enzyme that fulfils a similar enzymatic task as the GPx1 by detoxifying hydrogen peroxide. None of the pentathiepins altered the activity of either enzyme at a concentration of 25 µM (Figure 5b,c).

#### 2.2.2. Cytotoxic and Antiproliferative Activity

Distinct MTT cell viability and crystal violet cell proliferation assays were performed to assess the cytotoxicity of the compounds **1**–**6** on 14 human cancer cell lines. In the viability assay, cells with intact mitochondria and thus active dehydrogenases are competent to convert MTT to the corresponding insoluble formazan. The formazan can be photometrically detected after solubilization and allows for indirect conclusions about the viability of the cells as an IC_50_ value [32,33]. In the crystal violet assay, cells are stained by the binding of the dye to the chromatin, thus allowing for a quantification of biomass that remains adherent after a treatment in comparison with a control after a specific time [34]. Potency is defined as the growth inhibitory concentration 50% (G_I50_).

**Figure 6:**
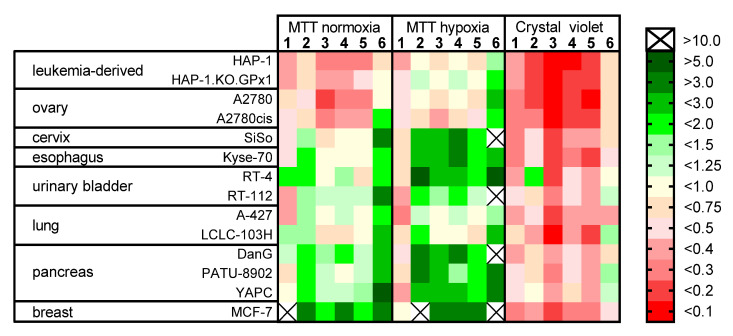
Heatmap summarizing the mean IC_50_ and GI_50_ values in micromolars after treatment of a panel of 14 human cancer cell lines with pentathiepins **1**–**6** determined with MTT assay under normoxic and hypoxic conditions, and with the crystal violet assay under normoxia. The corresponding values with SD are available in Table S3. n ≥ 3 independent experiments.

With the pentathiepins **1**–**6** IC_50_ (cell viability) and GI_50_ (cell growth) values in the low- or even sub-micromolar range were observed (Figure 6, Table S3). Notably, compound **6** was least cytotoxic with a mean of 2.36 µM throughout the panel, while **1** displayed a mean IC_50_ of 0.72 µM, representing the most potent compound. Most sensitive were cell lines derived from leukemia and ovary with mean IC_50_ values between 0.42 and 0.58 µM, while those from breast and pancreatic cancer were less susceptible (1.39 to 2.34 µM) to the treatment with the compounds.

In the crystal violet assay, the pentathiepins **1**–**6** gave sub-micromolar GI_50_ values over all cell lines, between 0.17 µM (**3**) and 0.57 µM (**6**). Cell lines that were most resistant to the compounds were the urinary bladder and the pancreatic cancer cell lines with mean GI_50_ values between 0.49 and 0.62 µM. Again, the weakest effect was observed for **6**. Interestingly, pentathiepin **1**, which did not affect the cellular viability of the MCF-7 breast cancer cell line up to 10 µM, strongly inhibited proliferation with a GI_50_ of 0.21 µM.

In addition to performing the cell viability assay under normoxia (19% atmospheric oxygen), incubations were also conducted under hypoxic conditions, i.e., atmospheric oxygen levels of 1% during the treatment for 48 h. Hypoxia is a favorable condition of the tumor microenvironment that is connected with poor outcome [35]. Therefore, the low oxygen condition mimics better the tumor environment. The biological effect of the pentathiepins under this condition provides insight into their dependency for oxygen, which has been postulated a key component in the proposed mechanism of action (Figure 3). Consistent with the proposed mechanism of action for five of the compounds, potency decreased under hypoxic conditions; in fact for **6**, the half-maximal inhibitory concentration exceeded the highest tested concentration of 10 µM in four cell lines (Figure 6, Table S3). As the exception, pentathiepin **1** showed no or potentiating effects regarding the cytotoxicity under hypoxia. In contrast to the other compounds, in some cell lines, the cytotoxic effects of **1** were more pronounced under oxygen-reduced incubation conditions (Figure 6), indicating a tangential role of oxygen availability for this pentathiepin. Especially for the breast cancer cell line MCF-7, the cytotoxicity increased substantially, indicated by a decrease of the IC_50_ from >10.0 µM to about 1.0 µM. Furthermore, the availability of atmospheric oxygen during treatment did not alter the effect on cell viability in three of the cell lines, namely, the cisplatin-resistant ovarian carcinoma cell line A2780cis and the lung carcinoma cell lines A427 and LCLC-103H.

To explore whether these observations were due to altered cell doubling rates under normoxic and hypoxic conditions, we determined the growth rates and subsequently the division times (Figure S49a). With respect to this parameter, there was no difference between the culturing under normoxic and hypoxic conditions. Hence, the altered sensitivity toward the treatment with pentathiepins was independent of changes in cell doubling rates.

Another question that could be answered was whether the growth rates of the cell lines are determining factors for the cytotoxicity of the pentathiepins. Rapidly dividing cells are generally more susceptible to chemotherapeutic agents. Thus, a Pearson correlation analysis based on the doubling times and IC_50_ or GI_50_ values, respectively, was performed (Figure S49b–d) and interpreted following statistics guidelines [36]. With regards to the inhibition of proliferation, a statistically significant moderate correlation with the doubling times was found for pentathiepins **4** (*r* = 0.65, *p* = 0.029) and **5** (*r* = 0.70, *p* = 0.017). The same analysis revealed a statistically significant correlation of the IC_50_ values with the cell division time, namely, for compounds **3** (*r* = 0.70, *p* = 0.017), **4** (*r* = 0.72, *p* = 0.012), **5** (*r* = 0.73, *p* = 0.010), and **6** (*r* = 0.70, *p* = 0.016). These findings indicate that rapidly dividing cells are more sensitive toward the treatment with these pentathiepins. The fraction of shared variance between the two variables (*R²*) ranged from 43 to 54%, meaning that about half of the effect can be related to the division time.

Consistent with previous publications, the high cytotoxicity of the pentathiepins was confirmed across the 14-cell panel of different tissue origins. In summary, pentathiepins **1**–**6** had IC_50_ values in the low- or even sub-micromolar range, independent from their potential to inhibit the GPx1. This indicates that the inhibition of the GPx1 is not the exclusive reason for the high cytotoxicity. However, compounds with a higher IC_50_ in the GPx1 assay such as **2** and **6** were slightly less cytotoxic in both MTT and crystal violet assays under normoxic incubation conditions.

Generally, the cell lines presented slightly different sensitivities toward the treatment with pentathiepins. Especially those from pancreatic or urinary bladder carcinoma had IC_50_ values that were above the overall cell line average, although viability and proliferation were strongly decreased. This underlines the necessity of testing multiple cell lines from different origin to obtain a better impression of the biological effect caused by a compound. We also found a moderate-to-high correlation between the cytotoxicity of the pentathiepins and the doubling time of the cells. This indicates that, in particular, rapidly dividing cells, such as cancer cells, were targeted.

When comparing the cytotoxic potency of the studied pentathiepins with those of well-known and widely applied anticancer agents tested previously under the same assay conditions [37], we found comparable results; in fact, they even exceeded the effects of clinically used anticancer drugs carboplatin, thiothepa, hydroxyurea, and busulfan in the same cell lines.

#### 2.2.3. GPx1 and Catalase Expression in Selected Cancer Cell Lines

As the pentathiepins targeted the GPx1 in vitro, it was important to assess whether toxicity of the compounds was related to cellular protein levels of GPx1. The selection of the cell lines was based on their expression profile of GPx1. Moreover, HAP-1, HAP-1.KO.GPx1, and A2780 were very sensitive towards treatment with pentathiepins, whereas Siso is highly susceptible to several chemotherapeutic agents [37]. In addition, three pancreatic cancer cell lines were also used in these investigations. The expression of GPx1 and that of catalase as another H_2_O_2_-detoxifying enzyme in the seven cell lines was quantified by Western blotting and normalized to total protein load per lane by using the TGX stain-free gel system from Bio-Rad [38,39,40].

**Figure 7:**
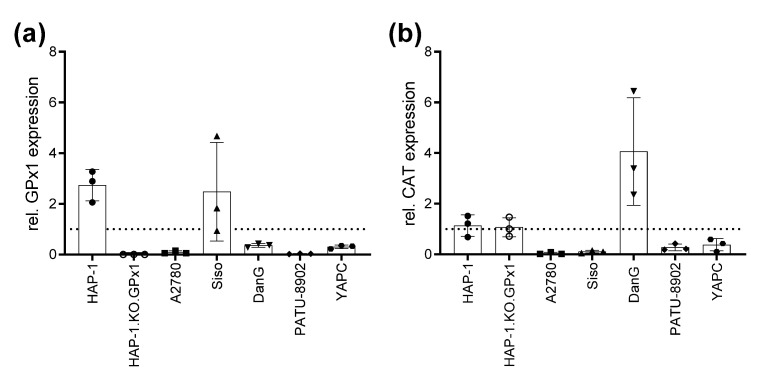
Relative expression of GPx1 (**a**) and catalase (**b**) in the seven cell lines used for selected biological assays to determine the effects of the pentathiepins **1**–**6** displayed as single values with mean and SD. Protein levels were determined by Western blotting and normalized to total protein load per lane (original images Figures S35–S38). For each protein, a mean was calculated throughout all cell lines (set to 1, dashed line) and all single values related to it. n = 3 independent experiments.

Figure 7 shows the semi-quantitative analysis of the Western blots, expressed as the relative protein expression across the seven cell lines. The cell lines with the highest expression of GPx1 were HAP-1 and Siso. As expected, the knockout cell line HAP-1.KO.GPx1 expressed no detectable enzyme, while A2780 had very low levels. The two pancreatic cancer cell lines DanG and YAPC contained equal amounts of the enzyme, while PATU-8902 had an expression as low as A2780. With regards to catalase, the parental HAP-1 and the corresponding GPx1 knockout cell line had similar enzyme levels, while A2780 and Siso had the lowest of the panel. Among the three pancreatic cancer cell lines, DanG had the highest expression of catalase, and PATU-8902 and YAPC had similarly low amounts.

Above-average GPx1 levels were detected for HAP-1 and Siso cells, which did not correlate with their sensitivity toward the pentathiepins. In particular, the fact that both HAP-1 and the GPx1-negative knockout variant responded similarly throughout the biological assays indicated a negligible impact of GPx1 expression on the cytotoxic mechanism of pentathiepins. Previous studies already compared these two cell lines with regards to their metabolism and antioxidant capacity, including several enzymes and intracellular GSH levels [22]. Additionally, we detected that catalase was most abundant in HAP-1, HAP-1.KO.GPx1, and the pancreatic cancer cell line DanG. Hence, catalase expression did not seem to have any impact on the sensitivity toward pentathiepins either.

#### 2.2.4. Generation of Intracellular ROS

**Figure 8:**
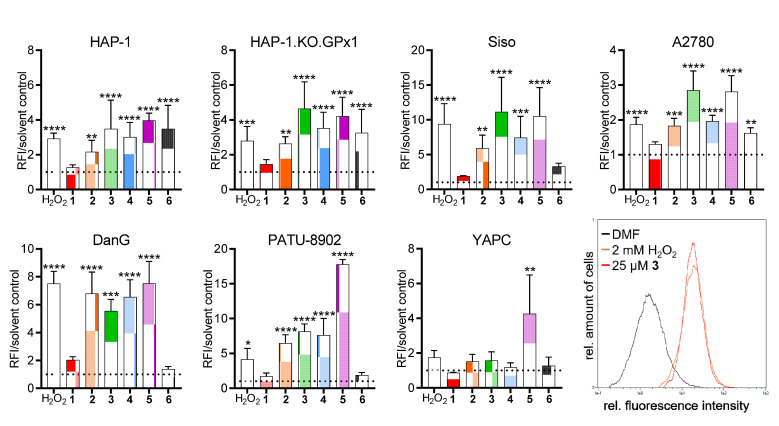
Relative intracellular levels of ROS after incubation for 15 min with 25 µM of pentathiepin **1**–**6** detected via flow cytometric DCFDA-based assay in seven cancer cell lines. Solvent only was used as the negative control, 2.0 mM of H_2_O_2_ was used as the positive control. Data are displayed as mean and SD, and a representative histogram from flow cytometric analysis is included (bottom right). All treatment conditions were related to the negative control sample (set to 1, dashed line) and statistical analysis performed in Prism 7 by one-way ANOVA and Dunnett’s multiple comparisons post hoc test. n ≥ 3 independent experiments, * *p* < 0.05, ** *p* < 0.01, *** *p* < 0.001, **** *p* < 0.0001.

According to the hypothesized mechanism of action (see Figure 3), pentathiepins can create oxidative stress via generation of reactive sulfur precursors. We have previously reported that a representative pentathiepin can increase the intracellular levels of ROS in two cancer cell lines [12]. This effect was thus further evaluated with the new compounds in seven cell lines (Figure 8). The cells were incubated for 15 min with 25.0 µM pentathiepin and subsequently analyzed by using the intracellular ROS sensor 2′,7′-dichlorodihydrofluorescein diacetate (DCFDA) by flow cytometry.

Treatment with pentathiepins **2**, **3**, **4**, and **5** resulted in a burst of ROS in all cell lines, in some cases even exceeding the effect of the positive control with H_2_O_2_ (2 mM) (Figure 8). Interestingly, **1** did not change the intracellular ROS levels in any of the cell lines and **6** only in three of the panel, including HAP-1, the corresponding GPx1-knockout line and A2780. In HAP-1 and HAP-1.KO.GPx1, the increase of ROS after treatment with pentathiepins was about two- to fourfold compared with the solvent control sample. In the Siso line, the rise in ROS levels ranged between 5- and 10-fold, while in A2780, it was roughly doubled or tripled. In the pancreatic cancer cell lines DanG and PATU-8902, high levels of intracellular ROS were detected after treatment with **2**, **3**, **4**, and **5**, while in YAPC, the only increase was monitored after incubation with **5**. In the latter cell line, a very small response was observed in general, as there was no significant reaction toward the H_2_O_2_ treatment either.

**Figure 9:**
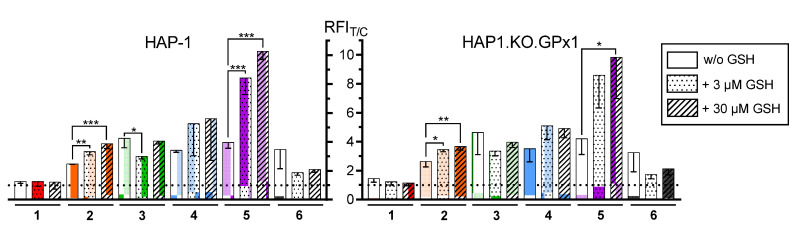
Relative intracellular levels of reactive oxygen species after an incubation for 15 min with pentathiepins (25 µM) omitting or adding GSH to the treatment medium detected via flow cytometric DCFDA-based assay in HAP-1 and HAP-1.KO.GPx1. Solvent only was used as negative control (set to 1, dashed line) and treatment conditions with either no, 3, or 30 µM of GSH were related to it. Data are displayed as mean and SD, and statistical analysis was performed in Prism 7 by one-way ANOVA and Dunnett’s multiple comparisons post hoc test. n ≥ 3 independent experiments, * *p* < 0.05, ** *p* < 0.01, *** *p* < 0.001.

As thiols are supposedly needed for the activation of the pentathiepins [24], the influence of additional glutathione added to the culture medium during treatment on the intracellular ROS levels was assessed. In these experiments, 3 or 30 µM of GSH were added to the medium containing 25 µM of pentathiepin or solvent, respectively (Figure 9).

A significant increase of intracellular ROS caused by pentathiepins **2** and **5** occurred after supplementing culture medium with glutathione, roughly doubling the effect of the compound alone. For **4**, an ascending trend was observed but not determined as statistically significant. On the contrary, compounds **3** and **6** showed a tendency toward decreased oxidative stress when GSH was added. Although not statistically significant, additional GSH halved the ROS-stimulating effect of the compound only. No change was observed for pentathiepin **1**, neither for treatment with omitted GSH (compare Figure 8) nor in a co-incubation.

**Figure 10:**
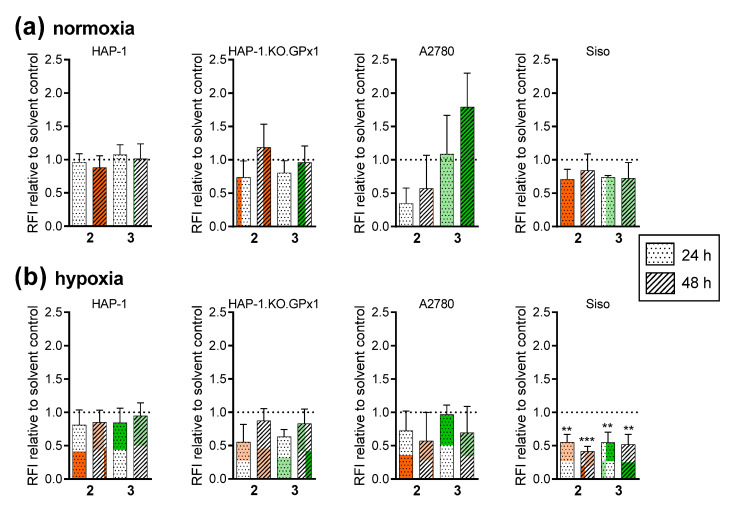
Relative intracellular levels of ROS after an incubation for 24 or 48 h with the respective pentathiepin **2** or **3** (IC_90_) under normoxic (**a**) or hypoxic (**b**) conditions detected via flow cytometric DCFDA-based assay in four cell lines. All treatment conditions were related to the negative control sample (set to 1; dashed line), displayed as mean with SD and statistical analysis was performed in Prism 7 by one-way ANOVA and Dunnett’s multiple comparisons post hoc test. n ≥ 3 independent experiments, ** *p* < 0.01, *** *p* < 0.001.

To investigate the long-term influence of treatment with pentathiepins on the ROS levels, we incubated the cells with the compounds **2** and **3** for 24 and 48 h (Figure 10a,b). Pentathiepin **2** was selected due to its probable potentiation by GSH and **3** because of its high potential to quickly induce ROS (see Figure 8 and Figure 9). In addition, this incubation was performed under both normoxic (19%) and hypoxic (1%) conditions to assess the impact of available oxygen (Figure 10a,b).

Under normoxic conditions, this long-term treatment resulted in no significant differences of cells incubated with pentathiepins relative to the solvent control cells. In A2780, trends were observed for both **2** and **3** that ROS levels increased with incubation time. In contrast, under hypoxic conditions, significant differences were detected in the Siso cell line, wherein the amount of ROS decreased by half for both representative pentathiepins on either time point.

The finding that pentathiepins increased intracellular ROS levels confirmed the results of previous studies, where it was postulated that reactive sulfur intermediates are released in the presence of a thiol [12,24]. The fluorescent pyrrolo-pyrazine containing compound **1** did not result in a boost of ROS in any of the tested cell lines, while all others did. The p-fluorobenzyolpiperazine (**5**) had the strongest effect, whereas the attachment of a p-tosyl in **6** led to a reduction. This might in part explain the lower cytotoxicity of **6**, but it contradicts the effects mediated by **1**. All three nicotinamide derivatives (**2**, **3**, **4**) gave similar results, irrespective of their terminal functional group. This taken together suggests that not only is the pentathiepin ring responsible for the induction of oxidative stress in the cells, but that the nature of the N-heterocycle also plays a critical role.

The induction of intracellular ROS is a common response to anticancer treatment, eventually resulting in cellular death due to oxidative stress. A predominant target for ROS-mediated damage is cellular DNA.

#### 2.2.5. Induction of DNA Strand Breaks

**Figure 11:**
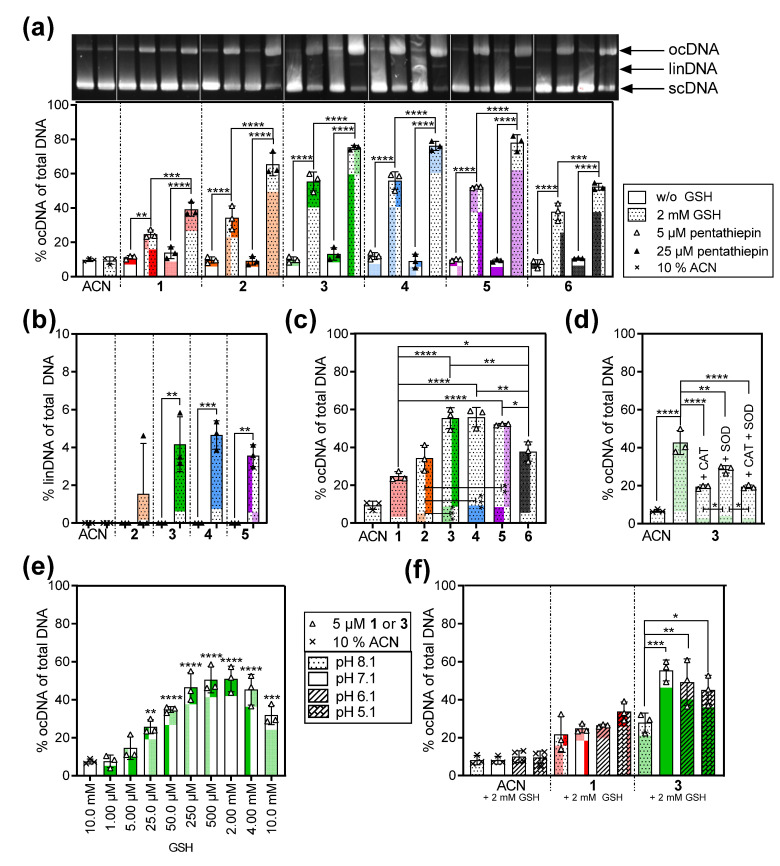
Cleavage of the plasmid DNA after treatment with pentathiepins for 20 h at 37 °C under different conditions. (a) Percentage of open circle (oc) DNA after incubation of pBR322 with 5 or 25 µM of pentathiepins 1–6 with or without 2 mM glutathione at pH 7.1. (b) Percentage of linearized (lin) DNA after incubation of pBR322 with 25 µM of pentathiepins 2–5 with 2 mM of GSH at pH 7.1. (c) Percentage of ocDNA displayed as inter-compound comparison. (d) Percentage of ocDNA after incubation of pBR322 with 5 µM of pentathiepin 3 and 2 mM of GSH in the absence and presence of 100 µg/mL of CAT or SOD or both at pH 7.1. (e) Percentage of ocDNA after incubation of pBR322 with 5 µM of pentathiepin 3 with GSH concentrations from 1.00 µM to 10.0 mM at pH 7.1, with asterisks indicating significant differences from solvent control with 10 mM of GSH. (f) Percentage of ocDNA after incubation of pBR322 with 5 µM of pentathiepin 1 or 3 with 2.00 mM of GSH and different buffer pH ranging from 5.1 to 8.1. Background cleavage was assessed by incubation with the solvent acetonitrile (ACN). Quantification was performed with Image Lab and data are displayed as mean, with single values and SD. Original images are provided in the Supplementary Materials (Figures S39–S45). Statistical analysis was performed with Prism 7 by one-way ANOVA and Dunnett’s or Tukey’s multiple comparisons post hoc test. n = 3 independent experiments. * *p* < 0.05, ** *p* < 0.01, *** *p* < 0.001, **** *p* < 0.0001.

We pursued two different strategies to assess the potential of the pentathiepins to damage DNA: a simple plasmid cleavage assay and the cellular Comet assay. The former is a straightforward method to investigate whether a test compound can cleave DNA in vitro, and if so, whether single and/or double strand breaks are created, while the latter is an ex vivo assay that measures DNA fragmentation in single cells. In the plasmid cleavage assay, a single strand break would relax the supercoiled plasmid (scDNA) and lead to the detection of open circular DNA (ocDNA), whereas a double-strand break would linearize the plasmid (linDNA). An agarose gel electrophoresis enables the discrimination of the distinct plasmid conformations due to their specific electrophoretic mobilities (Figure 11a).

It was found that the pentathiepins **1**–**6** caused plasmid DNA cleavage with statistically significantly different extents (Figure 11a,c). Compounds **3**, **4**, and **5** induced similar levels of single-strand breaks with roughly 55% at 5 µM and about 75% at 25 µM, while **2**, **6**, and **1** showed decreasing potential to damage DNA. The magnitude of cleavage is not only dependent on the presence of a thiol, in our case GSH, but also on the concentration of the pentathiepin (Figure 11a). A concentration of 25 µM resulted in a significantly higher percentage of open circular plasmid DNA than 5 µM, with a difference of about 15–30%.

This is the first time that the formation of double strand breaks by pentathiepins has been described (Figure 11b). This effect was detected for pentathiepins **2**, **3**, **4**, and **5** after incubation of the plasmid DNA with 25 µM compound, which resulted in about 4% linearized plasmid, corresponding to DNA with double-strand breaks.

Further experiments were conducted with selected pentathiepins to establish some of the conditions that may promote the cleavage effect. The investigations covered different GSH concentrations (Figure 11e) and pH of the buffer system (Figure 11f), which have both been described as modifiers of pentathiepin-mediated damage of plasmid DNA [6,41].

First, we investigated whether there is an optimal pentathiepin–thiol ratio by incubating plasmid DNA with 5 µM of the most active compound **3** and GSH concentrations between 1.0 µM and 10.0 mM (Figure 11e). The highest amount of ocDNA was detected in a GSH concentration range between 250 µM and 4.0 mM, corresponding to 50 to 800 equivalents of GSH per pentathiepin. The minimum amount of GSH that resulted in statistically significant plasmid cleavage was 25.0 µM, equal to a 1:5 ratio of pentathiepin to GSH.

In addition, the pH of the reaction buffer was modified to investigate the influence of this factor on the DNA cleavage potential of two different pentathiepins (Figure 11f). Pentathiepin **1** was selected as the least, and **3** as the most active plasmid damaging compound and the assay performed at pH 5.1, 6.1, 7.1, and 8.1 with 5 µM of each compound. For **1**, a trend favoring lower pH for higher cleavage effects was observed but was not proven to be statistically significant. However, pentathiepin **3** led to different relative amounts of ocDNA, with the highest observed at pH 7.1 (55%), 6.1 (49%), and 5.1 (45%). A remarkable decrease in cleaving was measured for pH 8.1 (28%) with about half of the amount of ocDNA detected than at pH 7.1.

To further investigate the plasmid cleaving mechanism, we repeated this experiment with pentathiepin **3**, and CAT or SOD were added (Figure 11d). Both antioxidative enzymes play a role in detoxifying DNA-damaging radicals such as H_2_O_2_ and superoxide, respectively [41]. As shown in Figure 11d, pentathiepin **3** significantly increased the fraction of damaged DNA to about 43% in comparison with the solvent control (7%). Supplementation with the anti-oxidative enzymes CAT or SOD reduced the amount of broken plasmid to 19% and 29%, respectively, while a combination of both enzymes did not exceed the effect of CAT alone (19%). Thus, both enzymes exerted a protective role but could not fully compensate for the damaging effect of the pentathiepin at the catalytic concentrations used.

The current findings that pentathiepins **1**–**6** cause cleavage of plasmid DNA by inducing both single and double strand breaks is consistent with our previous findings for another pentathiepin [12] and supports the theoretical postulated mechanism of action [24]. The reaction is dependent on a certain ratio of pentathiepin and GSH with a minimum ratio of 1:5. We can also associate the potential to induce oxidative stress with the ability to damage DNA, as the ROS boosting compounds **2**, **3**, **4**, and **5** were also the best DNA cleaving agents.

While previous publications mentioned an acidic environment as beneficial for cleavage effectiveness of varacin or the trithiol compound varacin C [41,42], we could not confirm this for pentathiepins **1**–**6**.

#### 2.2.6. Damage of Genomic DNA

**Figure 12:**
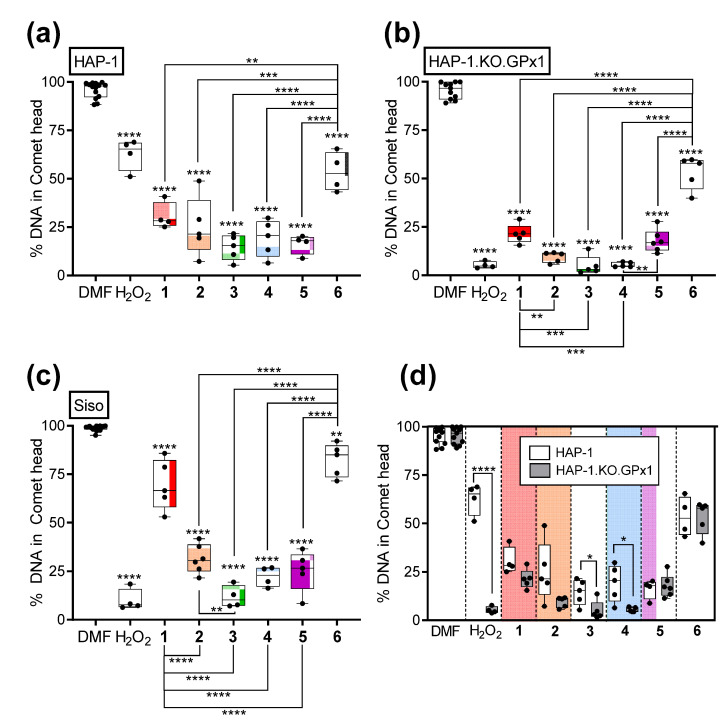
Percentage of intact DNA in the Comet head after treatment of HAP-1 (**a**), HAP-1.KO.GPx1 (**b**), and Siso (**c**) with 25 µM of pentathiepin **1**–**6** or 20 µM of H_2_O_2_ for 15 min at 0 °C as assessed with the Comet assay. (**d**) A direct comparison of HAP-1 and HAP-1.KO.GPx1 is displayed. Data are presented as box plots with single values, mean and minimum to maximum. Asterisks on boxes indicate difference from solvent control, asterisks on brackets denote inter-compound or inter-cell line variation. Statistical analysis was performed with Prism 7 by one-way ANOVA and Dunnett’s or Tukey’s multiple comparisons post hoc test. n ≥ 4 independent experiments. * *p* < 0.05, ** *p* < 0.01, *** *p* < 0.001, **** *p* < 0.0001.

Next, the potential of the pentathiepins **1**–**6** to damage nuclear DNA was investigated in the cell lines HAP-1, HAP-1.KO.GPx1, and Siso, with different expression levels of GPx1 and CAT (see Figure 7), by Comet Assay (Figure 12) with representative images in the Supplementary Materials (Figure S48).

All tested compounds damaged the DNA in each of the investigated cell lines at 0 °C but to different extents, while more than 95% of DNA remained intact in the negative controls. In HAP-1 and the corresponding GPx1-knockout cell line (Figure 12a,b), the pentathiepins had similar effects, with **6** being the least DNA-damaging agent. Interestingly, the effect of **1** was comparable to the other pentathiepins, causing damage levels of 70–80%. After treatment with **2**, **3**, **4**, or **5**, only 5 to 25% of genomic DNA remained intact. In Siso cells (Figure 12c), the most active agents were **3**, **4**, and **5**, followed by **2**, decreasing the amount of intact DNA to 12–25% and 30%, respectively. Pentathiepins **1** and **6** resulted in similar damage, leaving about 70–80% of DNA unbroken. This was a significant difference when compared with the more active pentathiepins **2**, **3**, **4**, and **5**.

As expected, the positive control with 20 µM H_2_O_2_ resulted in higher comet formation in GPx1-knockout cells, with 5% intact nuclear DNA compared to the parental cell line HAP-1 with 63% undamaged nuclear DNA (Figure 12d). This is consistent with the idea that GPx1 protects cells from DNA damage caused by H_2_O_2_. Surprisingly, for Siso, the cell line with the highest GPx1 expression, nuclear DNA damage was just as high as in the knockout cell line, with about 10% intact DNA remaining in the nucleus.

The findings of the Comet assay are to the most part consistent with the results obtained in the plasmid cleavage experiments. Pentathiepin **6** was the least damaging agent, and the level of DNA damage was similar for compounds **2**, **3**, **4**, and **5** in both assays. An exception was found for **1**, which was the least damaging compound in the plasmid assay but resulted in relatively high damage of genomic DNA in HAP-1 and HAP-1.KO.GPx1. Together with the fact that **1** did not result in oxidative stress in any cell line, this indicates a different mechanism of action.

With regards to the role of GPx1, it can be stated that the presence of this enzyme plays only a minor role if any in the cytotoxic activity of pentathiepins, considering the fact that both HAP-1 cell lines react similarly to the treatment with these compounds. The observed strong response of the Siso cell line in the positive control setting can be explained by the comparably low expression of CAT, which is more effective in decomposing high concentrations of H_2_O_2_ than GPx1.

#### 2.2.7. Intracellular Distribution of Pentathiepin **1**

**Figure 13:**
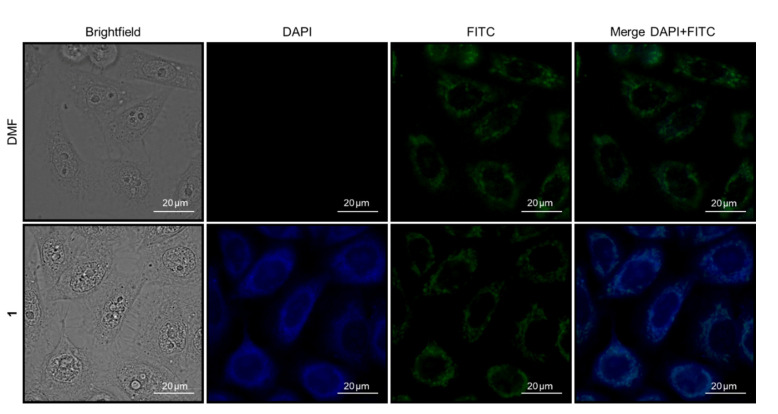
Representative images of Siso cells treated with either DMF or pentathiepin **1** (IC_90_) for 24 h prior to imaging with a Leica fluorescence microscope with a 63× oil immersion objective. The distribution of **1** was visualized in the DAPI channel (λ_ex_ = 325–407 nm, λ_em_ = 461 nm), and the autofluorescence of the cells in the FITC channel (λ_ex_ = 488 nm, λ_em_ = 525 nm). Cell morphology was imaged in bright field settings.

One of the investigated pentathiepins was specifically designed to allow intracellular tracking via fluorescence microscopy, namely, the aryl-conjugated pyrrolo-pyrazine-fused compound **1**. As we detected a high potential to damage DNA, we wanted to assess whether pentathiepins accumulate in the nucleus. Exemplarily, Siso cells were incubated with pentathiepin **1** and subsequently monitored under the fluorescent microscope (Figure 13).

The DAPI (4′,6-diamidino-2-phenylindole) channel (λ_ex_ = 325–407 nm, λ_em_ = 461 nm) was used to visualize the pentathiepin, while in the FITC (fluorescein isothiocyanate) channel (λ_ex_ = 488 nm, λ_em_ = 525 nm), the autofluorescence of the cells was detected.

The pentathiepin **1** exhibited fluorescent properties when excited; hence, it was feasible to examine the distribution in cells. As apparent in Figure 13, the compound was dispersed within the cytoplasm and did not accumulate in any cellular compartment. Importantly, no accumulation in the nucleus took place, suggesting that nuclear DNA may not be the target for this pentathiepin. This is in accordance with the inferior nuclear DNA cleavage capacity of this compound in contrast to the pentathiepins **2**–**5** in the Siso cell line (Figure 12c).

#### 2.2.8. Induction of Apoptosis

**Figure 14:**
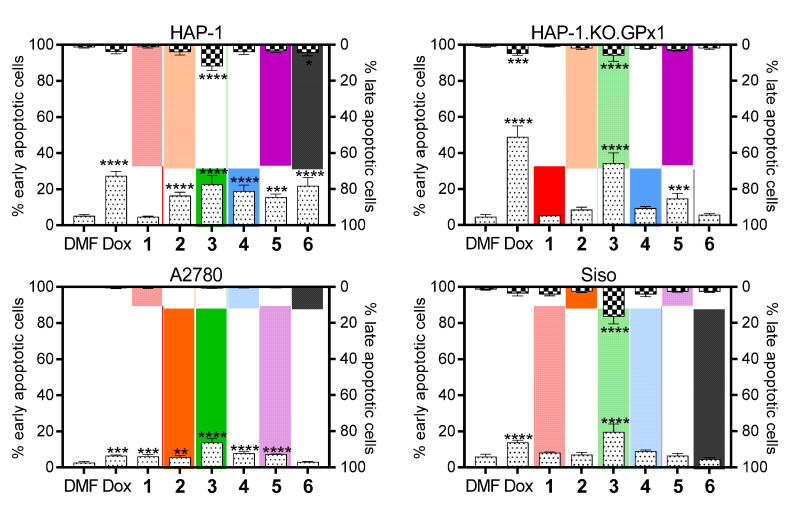
Induction of apoptosis assessed via annexin V/PI flow cytometric assay after treatment for 24 h with the respective pentathiepin **1**–**6** (IC_90_) or 0.5 µM of doxorubicin (Dox) as positive control. Negative control cells were incubated with the solvent DMF. Representative dot plots are provided in Figure S47. The fractions of early apoptotic cells (left *y*-axis, dotted bars) and late apoptotic cells (right *y*-axis, checkered bars) are displayed as mean with SD. Resulting gaps between the bars correspond to viable cells. Statistical analysis was performed with Prism 7 by one-way ANOVA and Dunnett’s multiple comparisons post hoc test. n = 3 independent experiments. * *p* < 0.05, ** *p* < 0.01, *** *p* < 0.001, **** *p* < 0.0001.

As reported in our earlier publication, a representative pentathiepin was capable of inducing apoptosis in cancer cell lines [12]. Here, we tested compounds **1**–**6** for their ability to trigger this particular modality of cell death in a subset of four cell lines: HAP-1, HAP-1.KO.GPx1, A2780, and Siso. A flow cytometric assay was performed, allowing us to discriminate between viable, early, and late apoptotic cells (representative dot plots can be found in Figure S47). FITC-labeled annexin V was used to assess the externalization of phosphatidyl serine as a hallmark of early apoptosis induction [43]. The addition of propidium iodide (PI) enabled the detection of late apoptotic cells as it only permeates porous cell membranes and exerts fluorescence once bound to double-stranded DNA. The cells were treated with the pentathiepins (IC_90_) for 24 h and were subsequently analyzed (Figure 14).

All new pentathiepins induced apoptosis, although to different extents and in a cell line-dependent manner. In HAP-1 cells, all pentathiepins except for **1** resulted in increased fractions of early apoptotic cells up to 23%, which was comparable to the doxorubicin-treated positive control. Compound **3** additionally increased the percentage of late apoptotic cells up to 12%. In the corresponding GPx1-knockout cell line, the effect of doxorubicin was higher (about 50%), whereas only two of the pentathiepins induced apoptosis, namely, **3** and **5**. The fractions of early apoptotic cells were 34 and 15%, respectively, and the population of late apoptotic cells 6% for **3**. In the ovarian carcinoma cell line A2780 the overall effects were marginal, but all compounds except **6** increased the percentage of early apoptotic cells to 6–14%, with **3** resulting in the highest amount. In the Siso cell line, pentathiepin **3** increased both the number of early and late apoptotic cells to about 20%, which exceeded the effects of doxorubicine (0.5 µM). These findings were further corroborated by the observation of morphological features such as membrane blebbing and cell shrinkage as particular hallmarks of apoptosis (Figure S51).

**Figure 15:**
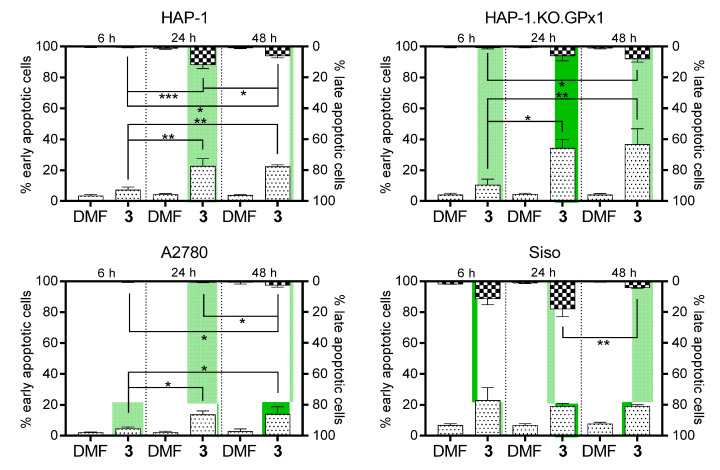
Induction of apoptosis in a time-dependent manner as assessed via annexin V/PI flow cytometric assay after treatment with the representative pentathiepin **3** (IC_90_) for 6, 24, or 48 h. Negative control cells were incubated with the solvent DMF. The fractions of early apoptotic cells (left *y*-axis, dotted bars) and late apoptotic cells (right *y*-axis, checkered bars) are displayed as mean and SD. Resulting gaps correspond to viable cells. The asterisks on brackets denote time-dependent differences. Statistical analysis was performed in Prism 7 by one-way ANOVA and Dunnett’s multiple comparisons post hoc test. n = 3 independent experiments. * *p* < 0.05, ** *p* < 0.01, *** *p* < 0.001.

For representative pentathiepin **3** as the most effective with regards to the induction of apoptosis, the effect was examined in a time-dependent manner at 6, 24, and 48 h (Figure 15).

In Siso cells, apoptosis was visible already after 6 h, while in HAP-1, HAP-1.KO.GPx1, and A2780, first effects were apparent after 24 h. Moreover, there was no increase of apoptotic cells detected between the incubation periods of 24 to 48 h. Hence, for further investigations, we focused on the time frame of 24 h.

The underlying mechanism of cell death for the pentathiepins was analyzed in order to explain their high cytotoxicity that was determined in the MTT and crystal violet assay. The induction of apoptosis was already described in our previous publication [12] and is confirmed herein for the new set of pentathiepins.

**Figure 16:**
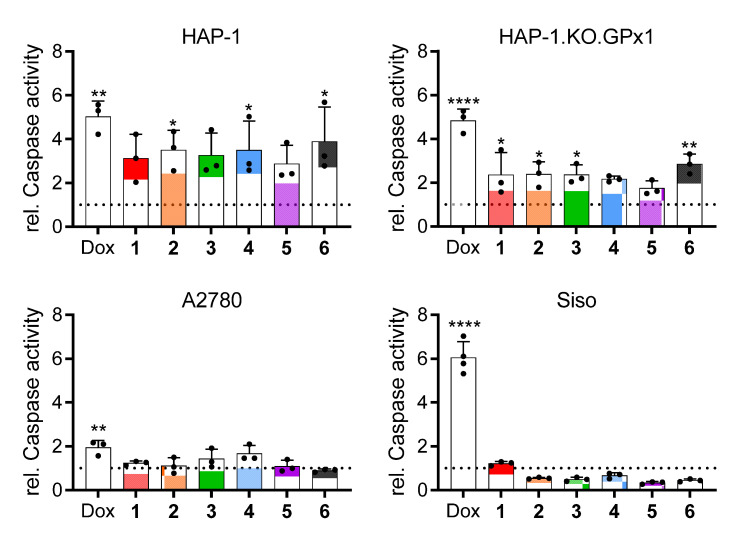
Activation of caspase-3 and -7 in HAP-1, HAP-1.KO.GPx1, A2780, and Siso after treatment for 24 h with the respective pentathiepins **1**–**6** (IC_90_) or 0.5 µM doxorubicin (Dox) as positive control in relation to an incubation with the solvent DMF. Statistical analysis was performed with Prism 7 by one-way ANOVA and Dunnett’s multiple comparisons post hoc test. n = 3 independent experiments. * *p* < 0.05, ** *p* < 0.01, **** *p* < 0.0001.

To further corroborate this mode of cell death, we performed a luminescent caspase assay by measuring caspase-3 (cas-3) and caspase-7 (cas-7) activities (Figure 16). Both are executioner caspases that are present as zymogens (35 kDa) and become active after being cleaved (fragment sizes 17 and 19 kDa) by initiator caspases. Activated cas-3 and -7 in turn have several other substrates, including PARP1. In this assay, a precursor with a caspase-specific target sequence was converted into its luminescent form by active caspase-3 or -7, thereby emitting light that can be quantified.

An activation of the caspase-3 and -7 was confirmed for the positive control with doxorubicin (0.5 µM) in all four cell lines (Figure 16). In HAP-1, the treatment with pentathiepins resulted in elevated caspase activity, but statistically significant results were only obtained for **2**, **4**, and **6**. Similarly, in the corresponding knockout cell line, all compounds increased the activity of caspases in relation to the solvent control. However, these findings were only statistically significant for the pentathiepins **1**, **2**, **3**, and **6**. In the cell lines A2780 and Siso, none of the compounds provoked the activation of caspase-3 or -7. Unexpectedly, for the Siso line, a decrease trend was detected instead.

**Figure 17:**
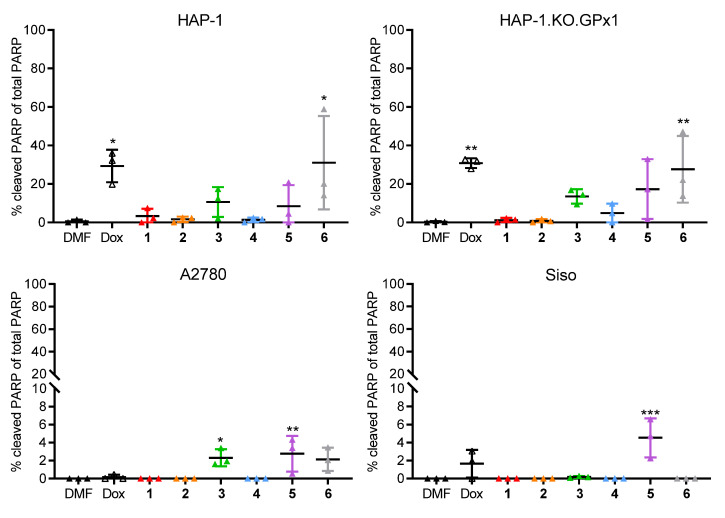
Detection of cleaved PARP1 via Western blotting after the treatment of HAP-1, HAP-1.KO.GPx1, A2780, and Siso for 24 h with the respective pentathiepins **1**–**6** (IC_90_) or the solvent DMF as negative control. A positive control was performed by incubating the cells with 0.5 µM of doxorubicin (Dox). Displayed is the percentage of cleaved PARP1 as single values with mean and SD. PARP1 levels were determined and subsequently normalized using the total protein load per lane (see original images Figures S29–S34). Statistical analysis was performed with Prism 7 by one-way ANOVA and Dunnett’s multiple comparisons post hoc test. n = 3 independent experiments. * *p* < 0.05, ** *p* < 0.01, *** *p* < 0.001.

In addition to general flow cytometric and microscopic analyses, Western blotting was performed to detect the apoptosis-relevant protein PARP1 (poly(ADP-ribose) polymerase 1) and its cleavage fragment (Figure 17 and Figures S29–S34). Full-length PARP1 (116 kDa) is involved in DNA repair, and a cleavage by caspases (fragment sizes 24 and 89 kDa) caused by caspase-3 and -7 is a hallmark of late apoptosis.

Here, the cells were treated for 24 h with the respective pentathiepins (IC_90_), and protein lysates were prepared and subsequently subjected to SDS-PAGE and Western blotting to quantify the cleavage of PARP1. All detected specific bands were normalized to the total protein load per lane by the TGX stain-free gel system from Bio-Rad [38,39,40].

Cleaved PARP1 was detected to statistically significant extents in both HAP-1 and HAP-1.KO.GPx1 after the treatment with the positive control doxorubicin. Incubation with **3** or **5** resulted in trends for increased levels. However, only the treatment with **6** caused relevant and significant quantities of cleaved PARP1. In A2780 and Siso cells, the fraction of 89 kDa PARP1 was very small and only confirmed after treatment with pentathiepins **3** or **5**. These results appear consistent with the previous caspase-3/-7 studies.

#### 2.2.9. No Evidence for Ferroptosis

Not only apoptosis may contribute to the mainly ROS-based cytotoxicity of the pentathiepins but also ferroptosis, which derives from iron-dependent oxidative damage of the cell membrane [15]. This form of programmed cell death is initiated via lipid peroxidation, which is normally held in check by the membrane-bound GPx4, an isoform of GPx1. As pentathiepins are potent GPx1 inhibitors, we indirectly investigated the possible role of GPx4 inhibition by precluding ferroptosis. Here, cells were incubated with a serial dilution of the respective pentathiepin, and either ferrostatin-1 (Fer-1) or vehicle control (DMSO) was added; Fer-1 is an established inhibitor of ferroptosis [15,44]. The cell viability was measured after 48 h, and the activity of the compounds after adding or omitting Fer-1 was compared (Figure S50).

If the compounds induced ferroptosis, the addition of Fer-1 would decrease the cytotoxicity compared to the control population, i.e., the resulting IC_50_ would be higher than that of pentathiepin-treated cells without the additive [45]. However, no effect on the IC_50_ of the pentathiepins was observed when a co-incubation with Fer-1 was performed in the four cell lines, indicating that the test compounds do not induce ferroptosis.

#### 2.2.10. Influence on Cell Cycle Progression

**Figure 18:**
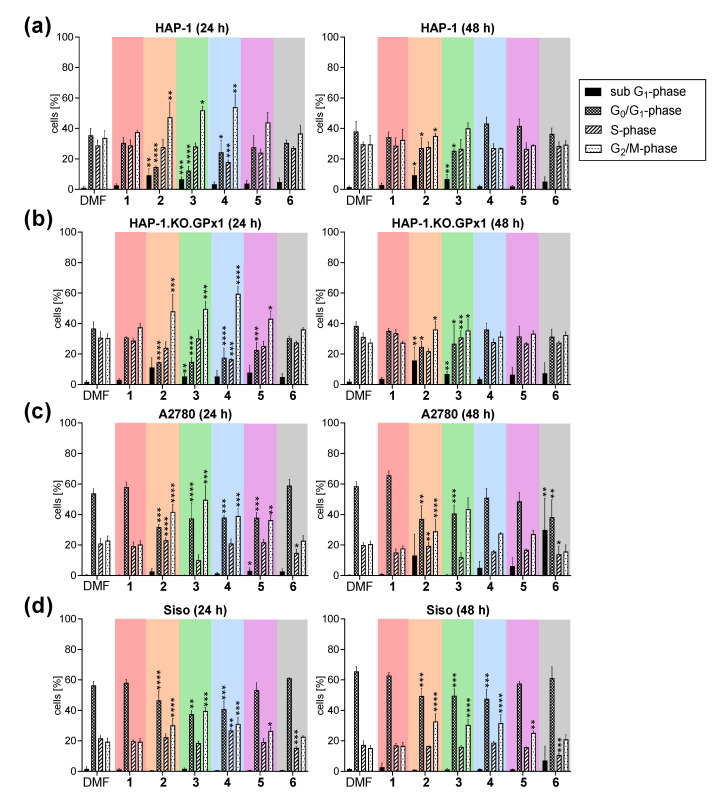
Distribution of cell cycle phases in HAP-1 (**a**), HAP-1.KO.GPx1 (**b**), A2780 (**c**), and Siso (**d**) after a treatment for 24 (left) and 48 h (right) with the respective pentathiepin (IC_90_) or the solvent DMF as negative control. Representative histograms are provided in Figure S46. The fractions of cells in either sub G_1_-, G_0_/G_1_-, S-, or G_2_/M-phase are displayed as mean and SD. Asterisks indicate differences between the solvent control and the treatment condition. Statistical analysis was performed in Prism 7 by one-way ANOVA and Dunnett’s multiple comparisons post hoc test. n = 3 independent experiments. * *p* < 0.05, ** *p* < 0.01, *** *p* < 0.001, **** *p* < 0.0001.

**Figure 19:**
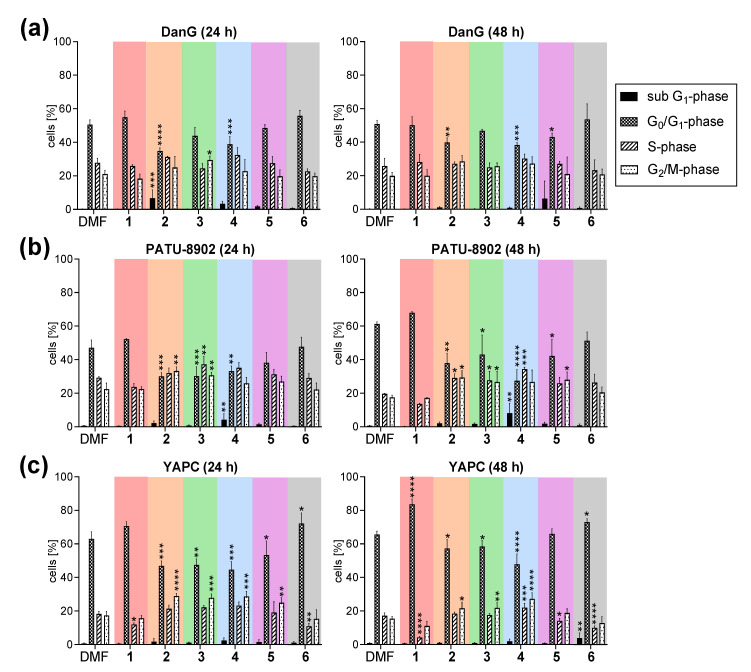
Distribution of cell cycle phases in DanG (**a**), PATU-8902 (**b**), and YAPC (**c**) after a treatment for 24 (left) and 48 h (right) with the respective pentathiepin (IC_90_) or the solvent DMF as negative control. Representative histograms are provided in Figure S46. The fractions of cells in either sub G_1_-, G_0_/G_1_-, S-, or G_2_/M-phase are displayed as mean and SD. Asterisks indicate differences between the solvent control and the treatment condition. Statistical analysis was performed in Prism 7 by one-way ANOVA and Dunnett’s multiple comparisons post hoc test. *n* = 3 independent experiments. * *p* < 0.05, ** *p* < 0.01, *** *p* < 0.001, **** *p* < 0.0001.

The potent cytotoxicity and the potential to inhibit the proliferation of cancer cells accompanied by DNA damage effects raised the question as to whether cell cycle progression might be affected as a result of treatment with pentathiepins. To answer this, the four cell lines HAP-1, HAP-1.KO.GPx1, A2780, and Siso (Figure 18), as well as the three pancreatic cancer cell lines DanG, PATU-8902, and YAPC (Figure 19), were treated with the test compounds at the IC_90_ concentrations and incubated for 24 and 48 h, followed by quantifying the relative numbers of cells in the different phases of the cell cycle (i.e., G_0_/G_1_, S and G_2_/M) as well as cell debris (i.e., sub G_1_) by flow cytometry.

For both HAP-1 and HAP-1.KO.GPx1 (Figure 18a,b), the most pronounced effects were measured after 24 h as a result of treatment with **2**, **3**, **4**, and **5**; mainly with increased amounts of cells in the sub G_1_-phase and G_2_/M-phase by up to 10 and 30%, respectively, while less cells were in the G_0_/G_1_-phase. No such effects were detected after treatment with either **1** or **6**. After 48 h, changes from the cell cycle phase distribution of treated cells compared with negative control cells were observed for **2** and **3**, displaying the same trend as after 24 h, but to a lesser extent regarding the G_2_/M-arrest.

In A2780 and Siso cells (Figure 18c,d), differences from a negative control were detected after treatment with all pentathiepins except for **1**. In most cases, changes appeared after 24 h, including decreased fractions of G_0_/G_1_-phase cells by up to 22% and enhanced amounts of cells in the G_2_/M-phase up to 27%. An exception was after incubation with **6**, where after 48 h, a very high relative amount of sub G_1_-phase cells (30%) was measured in A2780 at the expense of the G_0_/G_1_-phase. In the Siso line, the effects were similar after 48 h when compared to the treatment for 24 h.

We also investigated the influence of the pentathiepins on the cell cycle of the three pancreatic cancer cell lines DanG, PATU-8902, and YAPC, which turned out to be less sensitive than the other cell lines investigated (Figure 19). Still, with values in the low micromolar range (Table S3), pentathiepins potently inhibited growth of these cancer cell lines. For DanG cells (Figure 19a), slight changes were observed for **2**, **3**, **4**, and **5**, which included an increase of cells in the G_2_/M- and sub G_1_-phase by 6 and 8%, respectively, with a simultaneous decrease of G_0_/G_1_-phase cells by roughly 15%. Pentathiepins **1** and **6** caused no differences from a normal cell cycle. In PATU-8902 cells (Figure 19b), a significant increase of S-phase cells up to 14% was additionally observed, especially when treated with compounds **3** and **4**. Interestingly, in YAPC cells (Figure 19c) all six pentathiepins changed the distribution of the cell cycle phases. For **2**, **3**, **4**, and **5**, similar results were obtained compared with DanG and PATU-8902; however, remarkably, a very different cell cycle emerged after treatment with **1** and **6**. Here, a significant increase of G_0_/G_1_-phase cells by up to 18% occurred with a concurrent reduction of cells in the S-phase by roughly 7–13%, indicating a G_0_/G_1_-arrest. In short, incubations with pentathiepin resulted in cell cycle aberrations with either a G_2_/M-arrest mediated by **2**, **3**, **4**, and **5** or a G_0_/G_1_-arrest as result of **1** and **6**, with the latter only occurring in the YAPC cell line.

The progression of the cell cycle is connected with the integrity of genomic DNA, which is monitored at two different checkpoints. Damage to DNA leads to arrests at the respective phase of the cell cycle, which either result in repair via the intracellular machinery or in cell death. The latter is a mechanism to prevent the propagation of cells with a defective genetic constitution. Therefore, the cell cycle aberrations caused by the pentathiepins may be due to their DNA-damaging potential. The damage of DNA is most probably induced by oxidative stress, with the exception of **1,** which was the only pentathiepin that did not lead to a boost in ROS. For the anticancer agent bleomycin, which also causes single- and double-strand breaks in DNA through oxidative processes, similar G_2_/M arrests have been reported in cancer cell lines [46]. 

To elucidate the cell cycle-aberrating effect of **1**, we considered its chemical structure more closely. Here, we recognized a structural similarity of the pyrrolo-pyrazine scaffold with those of the so called aloisines (6-phenyl[5*H*]pyrrolo[2,3-b]pyrazines). These compounds have an impact on the cell cycle, specifically G_1_ and G_2_ arrests, by inhibiting cyclin-dependent kinases (CDKs) [47]. This suggests that the scaffold structure rather than the pentathiepin ring system of **1** may induce the dominating effects on the cell cycle.

#### 2.2.11. Structure–Activity Relationships

One aim of our study was to derive structure–activity relationships (SAR) from the biological evaluation, which may enable structural optimization of pentathiepins in the future. The six pentathiepins studied possess two distinct substructures: either a pyrrolo-pyrazine (**1**) or a nicotinamide scaffold (**2**–**6**) (Figure 2). Table 2 summarizes the SAR for the six new pentathiepins for 12 biological effects and compares them with those previously observed with compound (**c**). 

Apart from its fluorescent properties and an average potential to inhibit the GPx1, the backbone of pentathiepin **1** was accompanied by inferior biological activity. This especially entailed the induction of ROS and apoptosis (Figure 8 and Figure 14), and to a lesser extent the DNA cleaving capability (Figure 11 and Figure 12). However, the terminal methoxy group might act as hydrogen bond acceptor, which may explain the observed effects of this compound such as the inhibition of the GPx1 and the cytotoxic effects.

Taking into consideration the three nicotinamide-based pentathiepins **2**, **3**, and **4**, we found that the terminal morpholine (**3**) and diethylamine (**4**) moieties caused superior biological effects compared to the piperidine (**2**). These observations comprise the inhibition of the GPx1, the cytotoxic and antiproliferative activity, the induction of ROS, apoptosis, and DNA strand breaks (Figure 5a, Figure 6, Figure 8, Figure 11, Figure 12 and Figure 14). The diethylamine groups of **4** are likely to be more flexible than the closed piperidine of **2**. The derivative **3**, bearing another ring structure (morpholine), exhibited enhanced activity; the increased effects of this compound could be related to the morpholine as the oxygen offers free electron pairs for the interaction with putative targets by hydrogen bonding.

Pentathiepin **5**, with the p-fluorophenone scaffold, showed biological activity comparable with **3**, especially in the GPx1 assay and the induction of ROS or apoptosis (Figure 5a, Figure 8 and Figure 14). Again, the hydrogen bond accepting ability of the fluoro group may be responsible for this increase in activity.

The compound with the least biological activity was pentathiepin **6**, containing a p-tosylpiperazine. This is surprising because the sulfonamide group is bioisosteric to the amide group, which is present in compound **5**. Noticeably, this compound possesses an electron-rich aromatic system due to the electron-donating feature (+I effect) of the methyl substituent, but no ability to enter into hydrogen bonding. Moreover, the amide bond has a trigonal configuration, while the sulfonamide bond is tetrahedral. One or more of these structural properties could have resulted in the poor inhibitory potential towards the GPx1 and the lower induction of ROS and DNA damage (Figure 5a, Figure 8, Figure 11 and Figure 12).

Compound (**c**) in Figure 1 from the first generation of pentathiepins of our previous study [12] showed biological activities similar to **6**, especially regarding the cytotoxicity and the induction of ROS. Both compounds have terminal alkyl substituents causing a +I-effect. However, (**c**) inhibited the GPx1 in contrast to **6**. This activity of (**c**) could be related to the additional cyclic nitrogen in the pyrrolo-quinoxaline backbone, which is not present in **6**. This is supported by the fact that **1** is a pyrrolo-pyrazine derivative and also shows inhibitory activity towards GPx1. The investigated pentathiepins, in particular **3**, **4**, and **5**, had a stronger biological activity than (**c**) with respect to all assays that were performed.

For future optimization, the piperazine scaffold is preferred over the pyrrolo-pyrazine backbone. Moreover, substituents with electronegative properties as well as providing a free electron pair, e.g., a fluorine or morpholine, may impart superior biological activity of the pentathiepins.

## 3. Materials and Methods

### 3.1. Chemistry

#### 3.1.1. General

All reactions were performed under a nitrogen atmosphere by using oven-dried standard Schlenk glassware. The completely dried *N*,*N*’-dimethylformamide (DMF, 99.8%, extra dry, stored over molecular sieves), acetonitrile (ACN, 99%, extra dry, stored over molecular sieves), and diisopropylethylamine (DIPEA) were purchased from Acros organics (Fair Lawn, NJ, USA) and used as received for all air and/or moisture-sensitive reactions. The 6-bromo nicotinic acid, 1-ethynyl-4-methoxybenzene, 3-[bis(dimethylamino)methyliumyl]-3*H*-benzotriazole-1-oxide hexafluorophosphate (HBTU), 3,3′-diethoxy propyne, p-fluoro benzoyl chloride, and p-toluene sulfonyl chloride (PTSC) were purchased from TCS Germany (Duisburg, Germany) and employed without further purification.

^1^H NMR (300 MHz) and ^13^C NMR (75 MHz) spectra were recorded on a Bruker Avance II-300 spectrometer (Karlsruhe, Germany). Chemical shifts δ are given in parts per million (ppm), and the solvent residual peak (CDCl_3_: ^1^H, δ = 7.27; ^13^C, δ = 77.00) was used as an internal standard. Peak multiplicities are specified as follows: s, singlet; d, doublet; t, triplet; q, quartet; m, multiplet; br, broad. APCI-MS (*m/z*) spectra were recorded on an Advion MS, Ithaca, US. Macheray-Nagel silica gel 60 F254 plates(Düren, Germany) were used for thin-layer chromatography (TLC), and detection was achieved by UV light. Column chromatography was performed on Acros organics silica gel 60 (35–70 μm). The single X-ray crystal structure experiments were conducted by using a STOE IPDS2T and a diffraction source with a fine-focused sealed molybdenum tube; respective experimental details are given in the Supplementary Information (SI). An Elementar Vario MICRO cube(Langenselbold, Germany) was used for the experimental determination of elemental configurations of final pure products.

#### 3.1.2. General Procedure for Sonogashira Cross-Coupling Reaction

A 25 mL oven-dried Schlenk tube was charged with 2 mol % of palladium (II) acetate (Pd(OAc)_2_), 5 mol % of phosphine ligand (PPh_3_) or PdCl_2_(PPh_3_)_2_ (2–3 mol %), 3 mol % copper(I) iodide (CuI), and 1–2 mmol of chloro- or bromo-heterocyclic derivative under nitrogen (N_2_) atmosphere, and the resultant mixture was dissolved in 5 mL of dry ACN or DMF. The reaction mixture was stirred for 5 min and supplied with 2 equiv. of 3,3′-diethoxy propyne and 5 equiv. of TEA or DIPEA. This was followed by stirring at 60 °C for 3–4 h. After consumption of the starting materials (monitored by TLC/TLC-MS), the solvent was removed under vacuum, and the residue was purified by column chromatography in hexane/EtOAc (10% to 30%) solvent system to afford the desired product.

##### **1**: (**D**) 2-(3,3-Diethoxypropynyl)-6-(4-methoxyphenyl)-5*H*-pyrrolo[2,3-*b*]pyrazine

The general procedure for Sonogashira coupling was applied using **C** (0.50 g, 1.64 mmol) yielding **D** as red oil in 62% (0.357 g, 1.017 mmol) yield. ^1^H NMR (300 MHz, DMSO-d6) δ: 1.20 (t, J = 7.18 Hz, 6 H), 3.56–3.66 (m, 2 H), 3.66–3.79 (m, 2 H), 3.84 (s, 3 H), 5.60 (s, 1 H), 7.00–7.13 (m, 3 H), 7.99 (d, J = 8.69 Hz, 2 H), 8.33 (s, 1 H), 12.63 (br s, 1 H).

##### **2**: (**Y a**) (6-(3,3-Diethoxypropynyl)pyridin-3-yl)(piperidinyl)methanone

The general procedure for the Sonogashira reaction was applied using “**X a**” (1.94 g, 7.16 mmol), yielding the product as reddish-brown oil. The formation of “**Y a”** was confirmed by (+ve) APCI-MS *m/z* = 316.18 [M] calcd. for C_18_H_24_N_2_O_3_ [M], found: 317.22 *m/z* [M+H]. Without further purification, compound “**Y a”** was used in the next reaction.

##### **3**: (**Y b**) (6-(3,3-Diethoxypropynyl)pyridin-3-yl)(morpholino)methanone

Spectral data of the compound are in agreement with previous reports [48]. The general procedure for the Sonogashira reaction was applied using “**X b**” (1.00 g, 3.68 mmol) yielding the product as dark red liquid in 73% (0.85 g, 2.68 mmol) yield. ^1^H NMR (300 MHz, CDCl_3_) δ: 0.97–1.29 (m, 6 H), 2.67–2.71 (m, 2 H), 3.51–3.77 (m, 10 H), 5.40 (s, 1 H), 7.44 (d, J = 8.31 Hz, 1 H), 7.65 (dd, J = 7.74 Hz, 2.08 Hz, 1 H), 7.89 (s, 1 H); ^13^C NMR (75 MHz, CDCl3) δ: 14.4 (s, 1 C), 20.3 (s, 1 C), 30.7 (s, 1 C), 35.7 (s, 1 C), 37.9 (s, 1 C), 60.6 (s, 1 C), 66 (s, 1 C), 82.8 (s, 1 C), 85.1 (s, 1 C), 90.8 (s, 1 C), 126.4 (s, 1 C), 129.8 (s, 1 C), 134.7 (s, 1 C), 142.7 (s, 1 C), 147.6 (s, 1 C), 161.8 (s, 1 C), 166.4 (s, 1 C); (+ve) APCI-MS *m/z* = 318.16 [M] calcd. for C_17_H_22_N_2_O_4_ [M], found: 319.16 *m/z* [M+H].

##### **4**: (**Y c**) 6-(3,3-Diethoxypropynyl)-*N,N’*-diethylnicotinamide

The spectral data of compound “**Y c**” are in agreement with the literature [48]. The general procedure for the Sonogashira reaction was applied using “**X c**” (1.00 g, 3.89 mmol) yielding the product as reddish oil in 78% (0.923 g, 3.03 mmol) yield. ^1^H NMR (300 MHz, CDCl_3_) δ: 1.14 (t, J = 6.99 Hz, 12 H), 3.13 (br s, 2 H), 3.43 (br s, 2 H), 3.49–3.61 (m, 2 H), 3.64–3.76 (m, 2 H), 5.38 (s, 1 H), 7.38–7.43 (m, 1 H), 7.58 (dd, J = 8.12 Hz, 2.08 Hz, 1 H), 8.47 (s, 1 H); ^13^C NMR (75 MHz, CDCl3) δ: 14.7 (s, 1 C), 20.6 (s, 1 C), 31 (s, 1 C), 36.1 (s, 1 C), 39.3 (s, 1 C), 43.1 (s, 1 C), 60.9 (s, 1 C), 83.3 (s, 1 C), 85 (s, 1 C), 91.2 (s, 1 C), 126.7 (s, 1 C), 132 (s, 1 C), 134.2 (s, 1 C), 142.4 (s, 1 C), 147.2 (s, 1 C), 162.1 (s, 1 C), 167.5 (s, 1 C); (+ve) APCI-MS *m/z* = 304.18 [M] calcd. for C_17_H_24_N_2_O_3_ [M], found: 305 *m/z* [M+H].

##### **5**: (**Y d**) (4-(6-(3,3-Diethoxypropynyl)nicotinoyl)piperazinyl)(4-fluorophenyl)methanone

The general procedure for the Sonogashira coupling was applied using “**X d**” (0.39 g, 1.00 mmol), yielding the product as a red oil. The formation of “**Y d**” was confirmed by (+ve) APCI-MS *m/z* = 439.48 *m/z* [M] calcd. for C_24_H_26_FN_3_O_4_ [M], found: 440.1 *m/z* [M+H]. The product was used further without additional purification.

##### **6**: (**Y e**) (6-(3,3-Diethoxypropynyl)pyridin-3-yl)(4-tosylpiperazinyl)methanone

The general procedure for the Sonogashira reaction was applied using “**X e**” (0.425 g, 1.00 mmol), yielding the product as a red liquid. The formation of “**Y e**” was confirmed by (+ve) APCI-MS *m/z* = 471.18 *m/z* [M] calcd. for C_24_H_29_N_3_O_5_S [M], found: 472.1 *m/z* [M+H]. The product was used further without additional purification.

#### 3.1.3. Synthesis of Pentathiepins

General procedure: An oven-dried 25 mL Schlenk flask was charged with the alkyne precursor, 0.5 equiv. of (Et_4_N)_2_[MoO(S_4_)_2_] and 1 equiv. of elemental sulfur under inert gas atmosphere (N_2_). The mixture was dissolved in a dry polar non-protonating organic solvent (DMF or ACN) and allowed to react while stirring at 50 °C. The progress of the reaction was monitored with TLC. After the reaction was completed, the crude product mixture was concentrated under reduced pressure and purified through silica gel column chromatography with hexane/EtOAc (5 to 20%) as the mobile phase. The crystal and refinement data for the X-ray structural analyses of pentathiepins **2**, **3**, and **4** are reported in Table S1.

##### **1**: 11-Ethoxy-2-(4-methoxyphenyl)-3*H*-[1,2,3,4,5]pentathiepino[6′,7′:3,4]pyrrolo[1,2-*a*]pyrrolo[2,3-*e*]pyrazine

The general procedure for pentathiepins was applied using **IV** (0.355 g, 1.01 mmol) yielding the desired pentathiepin **1** (0.0845 g, 0.181 mmol, 18%) as red amorphous solid; ^1^H NMR (300 MHz, DMSO-d_6_) δ: 1.44–1.57 (m, 3 H), 1.99 (s, 3 H), 4.56 (q, J = 6.92 Hz, 2 H), 6.98–7.09 (m, 4 H), 7.87 (d, J = 8.69 Hz, 1 H), 8.48 (s, 1 H), 12.59 (s, 1 H); (-ve) APCI-MS = 464.98 *m/z* calcd. for C_18_H_15_N_3_O_2_S_5_ [M], found: 464.1 *m/z* [M-H]; elemental analysis calcd. for C_18_H_15_N_3_O_2_S_5_: C 46.43, H 3.25, N 9.02, S 34.43; found: C 46.55, H 3.19, N 8.98, S 35.36. HPLC: t_R_ = 5.67 min, purity (@ λ = 250 nm) = 97.8%

##### **2**: (6-Ethoxy-[1,2,3,4,5]pentathiepino[6,7-*a*]indolizin-9-yl)(piperidinyl)methanone

The general procedure for pentathiepins was applied using compound “**Y a**” (0.5 g, 1.58 mmol) yielding the product **2** (0.421 g, 0.97 mmol, 62%) as a yellow amorphous solid. ^1^H NMR (300 MHz, CDCl3) δ: 0.87 (d, J = 6.80 Hz, 2 H), 1.27 (br s, 2 H), 1.48 (t, J = 6.99 Hz, 3 H), 1.69 (br s, 2 H), 3.56 (br s, 4 H), 4.45–4.60 (m, 2 H), 6.80–6.87 (m, 1 H), 7.50 (dd, J = 9.25 Hz, 0.94 Hz, 1 H), 7.95 (s, 1 H); ^13^C NMR (75 MHz, CDCl3) δ: 15.6 (s, 1 C), 24.4 (s, 2 C), 72.3 (s, 2 C), 74.7 (s, 1 C), 92.3 (s, 1 C),110.9 (s, 1 C), 113.8 (s, 1 C), 118 (s, 1 C), 119.8 (s, 1 C), 121.2 (s, 1 C), 121.7 (s, 1 C), 129.6 (s, 1 C), 141.1 (s, 1 C), 167.1 (s, 1 C); (+ve) APCI-MS *m/z* = 430 calcd. for C_16_H_18_N_2_O_2_S_5_ [M], found: 431.1 *m/z* [M+H]; elemental analysis calcd. for C_16_H_18_N_2_O_2_S_5_: C 44.63; H 4.21; N 6.51; S 37.22; found: C 44.55, H 4.09, N 6.97, S 37.66. HPLC: t_R_ = 2.13 min, purity (@ λ = 250 nm) = 99.8%

##### **3**: (6-Ethoxy-[1,2,3,4,5]pentathiepino[6,7-*a*]indolizin-9-yl)(morpholino)methanone

The general procedure for pentathiepins was applied using compound “**Y b**” (0.5 g, 1.57 mmol) yielding the desired pentathiepin **3** (0.373 g, 0.8635 mmol, 55%) as an orange-red crystalline solid. ^1^H NMR (300 MHz, CDCl3) δ: 1.49 (t, J = 6.99 Hz, 3 H), 3.50–3.84 (m, 8 H), 4.47–4.62 (m, 2 H), 6.81 (dd, J = 9.25 Hz, 1.32 Hz, 1 H), 7.51 (dd, J = 9.25 Hz, 0.94 Hz, 1 H), 7.98 (t, J = 1.32 Hz, 1 H); ^13^C NMR (75 MHz, CDCl3) δ: 15.5 (s, 1 C), 66.8 (s, 2 C), 72.3 (s, 2 C), 110.9 (s, 1 C), 113.8 (s, 1 C), 118.2 (s, 1 C), 119.3 (s, 1 C), 120.6 (s, 2 C), 121.9 (s, 1 C), 129.5 (s, 1 C), 141.1 (s, 1 C), 167.3 (s, 1 C); (+ve) APCI-MS *m/z* = 431.98 calcd. for C_15_H_16_N_2_O_3_S_5_ [M], found: 433.1 *m/z* [M+H]; elemental analysis calcd. for C_15_H_16_N_2_O_3_S_5_: C 41.65, H 3.73, N 6.48, S 37.05; found: C 41.55, H 3.19, N 6.17, S 37.66. HPLC: t_R_ = 1.41 min, purity (@ λ = 250 nm) = 99.6%

##### **4**: 6-Ethoxy-*N*,*N’*-diethyl-[1,2,3,4,5]pentathiepino[6,7-*a*]indolizine-9-carboxamide

The general procedure for pentathiepins was applied using compound “**Y c**” (0.5 g, 1.643 mmol) yielding the desired product **4** (0.316 g, 0.755 mmol, 46%) as bright yellow crystalline solid. ^1^H NMR (300 MHz, CDCl3) δ: 1.22 (t, J = 7.18 Hz, 6 H), 1.48 (t, J = 6.99 Hz, 3 H), 3.45 (br s, 4 H), 4.52 (dd, J = 7.18 Hz, 2.27 Hz, 2 H), 6.83 (dd, J = 9.06 Hz, 1.51 Hz, 1 H), 7.51 (dd, J = 9.06 Hz, 1.13 Hz, 1 H), 7.86–7.92 (m, 1 H); ^13^C NMR (75 MHz, CDCl3) δ: 15.1 (s, 1 C), 71.9 (s, 1 C), 110 (s, 1 C), 113.2 (s, 1 C), 117.8 (s, 1 C), 119.1 (s, 1 C), 119.7 (s, 1 C), 122 (s, 1 C), 125.5 (s, 1 C), 129.2 (s, 1 C), 135 (s, 1 C), 135.9 (s, 1 C), 140.6 (s, 1 C), 145 (s, 1 C), 167.5 (s, 1 C); (+ve) APCI-MS *m/z* = 418.00 calcd. for C_15_H_18_N_2_O_2_S_5_ [M], found: 419.1 *m/z* [M+H]; elemental analysis calcd. for C_15_H_18_N_2_O_2_S_5_: C 43.04, H 4.33, N 6.69, S 38.29; found: C 43.15, H 4.19, N 6.28, S 38.66. HPLC: t_R_ = 1.89 min, purity (@ λ = 250 nm) = 99.3%

##### **5**: (6-Ethoxy-[1,2,3,4,5]pentathiepino[6,7-*a*]indolizin-9-yl)(4-(4-fluorobenzoyl)piperazinyl)methanone

The general procedure for pentathiepins was applied using compound “**Y d**” (0.410 g, 0.932 mmol) yielding the desired pentathiepin **5** (0.298 g, 0.540 mmol, 57%) as yellow amorphous solid. ^1^H NMR (300 MHz, CDCl3) δ: 1.49 (t, J = 6.99 Hz, 3 H), 3.67 (br s, 8 H), 4.55 (qd, J = 7.05 Hz, 2.27 Hz, 2 H), 6.78–6.84 (m, 1 H), 7.09–7.18 (m, 2 H), 7.41–7.48 (m, 2 H), 7.51 (dd, J = 9.25 Hz, 0.94 Hz, 1 H), 8.00 (t, J = 1.32 Hz, 1 H); ^13^C NMR (75 MHz, CDCl3) δ: 16 (s, 1 C), 72.7 (s, 5 C), 111.6 (s, 1 C), 114.3 (s, 1 C), 116.1 (s, 1 C), 116.4 (s, 1 C), 118.8 (s, 1 C), 119.5 (s, 1 C), 120.8 (s, 1 C), 122.5 (s, 1 C), 129.8 (s, 1 C), 129.9 (s, 1 C), 131.3 (s, 1 C), 141.6 (s, 1 C), 165.7 (s, 1 C), 167.6 (s, 1 C), 168.1 (s, 1 C), 170.2 (s, 1 C); (+ve) APCI-MS = 553.01 *m/z* calcd. for C_22_H_20_FN_3_O_3_S_5_ [M], found: 554 *m/z* [M+H]; elemental analysis calcd. for C_22_H_20_FN_3_O_3_S_5_: C 47.72, H 3.64, N 7.59, S 28.95; found: C 47.55, H 3.49, N 7.28, S 27.66. HPLC: t_R_ = 1.49 min, purity (@ λ = 250 nm) = 100.0%

##### **6**: (6-Ethoxy-[1,2,3,4,5]pentathiepino[6,7-*a*]indolizin-9-yl)(4-tosylpiperazinyl)methanone

The general procedure for pentathiepins was applied using compound “**Y e**” (0.440 g, 0.933 mmol) yielding the desired pentathiepin **6** (0.393 g, 0.671 mmol, 72%) as fine yellow amorphous solid. ^1^H NMR (300 MHz, CDCl_3_) δ: 1.46 (t, J = 6.99 Hz, 3 H) 2.46 (s, 3 H) 3.04 (br s, 4 H) 3.72 (br s, 4 H) 4.44–4.59 (m, 2 H) 6.71 (dd, J = 9.44 Hz, 1.51 Hz, 1 H) 7.36 (m, J = 7.93 Hz, 2 H) 7.47 (dd, J = 9.25 Hz, 0.94 Hz, 1 H) 7.60–7.67 (m, 2 H) 7.91 (t, J = 1.13 Hz, 1 H); ^13^C NMR (75 MHz, CDCl_3_) δ: 15.5 (s, 1 C), 21.5 (s, 1 C), 45.9 (s, 4 C), 72.2 (s, 1 C), 111.1 (s, 1 C), 113.8 (s, 1 C), 118.2 (s, 1 C), 119.1 (s, 1 C), 120.1 (s, 1 C), 122.2 (s, 1 C), 127.7 (s, 1 C), 129.4 (s, 2 C), 129.9 (s, 2 C), 132.2 (s, 1 C), 141.1 (s, 1 C), 144.2 (s, 1 C), 167.4 (s, 1 C); (+ve) APCI-MS = 585 *m/z* calcd. for C_22_H_23_N_3_O_4_S_6_ [M], found: 586.1 *m/z* [M+H]; elemental analysis calcd. for C_22_H_23_N_3_O_4_S_6_: C 45.11, H 3.96, N 7.17, S 32.84; found: C 45.55, H 3.39, N 7.68, S 31.56. HPLC: t_R_ = 1.58 min, purity (@ λ = 250 nm) = 100.0%

### 3.2. Biology

#### 3.2.1. Materials

Cell culture media RPMI1640 and IMDM, phosphate buffered saline (PBS), and stable glutamine and penicillin/streptomycin were obtained from PAN Biotech (Aidenbach, Germany). From Sigma-Aldrich (Taufkirchen, Germany), were purchased 2′,7′-dichlorofluorescein diacetate (DCFDA), propidium iodide (PI), hydrogen peroxide (H_2_O_2_, 30% *w/v* in water), *tert*-butylhydroperoxide (*t*-BHP, 70% *w/v* in water), fetal calf serum (FCS), glutathione (GSH), glutathione disulfide (GSSG), baker’s yeast glutathione reductase (GR), bovine erythrocyte glutathione peroxidase 1 (GPx1), bovine liver catalase (CAT), secondary antibody anti-rabbit-HRP conjugate, Mueller Hinton Broth 2 (MH2, cation-adjusted) medium, protease inhibitor cocktail (PIC, containing protease and peptidase inhibitors), bovine serum albumin (BSA), ferrostatin-1 (Fer-1), and Fluoromount^TM^ mounting medium for microscopy. Dimethylsulfoxide (DMSO) and DMF for cell culture, glutaraldehyde, reduced nicotinamide adenine dinucleotide phosphate (NADPH), lysogeny broth (LB) medium, ROTI^®^nanoquant, ribonuclease A (RNase A), Tween-20, skim milk powder, and low melting point agarose were purchased from Carl Roth (Karlsruhe, Germany). The primary antibodies for poly(ADP-ribose) polymerase 1 (PARP1), caspase-3 and -7, and GPx1 were purchased from Cell Signalling Technology (Leiden, the Netherlands), and the antibody for CAT from Calbiochem Merck (Darmstadt, Germany). From Alfa Aesar (Haverhill, MA, USA), we obtained 3-(4,5-dimethylthiazol-2-yl)-2,5-diphenyltetrazolium bromide (MTT). Doxorubicin was a gift of Pharmacia and Upjohn (Stockholm, Sweden), and propidiumiodide (PI) was from Applichem (Darmstadt, Germany). All reagents, equipment, and software for Western blotting were from Bio-Rad (Feldkirchen, Germany), and the annexin V-FITC/PI apoptosis detection kit was from Miltenyi Biotec (Teterow, Germany). The pBR322 plasmid DNA, microscopic glass slides, and protein ladder were purchased from Thermo Fisher (Waltham, MA, USA); agarose from GeneOn (Ludwigshafen, Germany); GelRed^®^ from Biotium (Hayward, CA, USA); and *N*-lauryl-sarcosinate from Merck (Darmstadt, Germany). For measuring microtiter plates, we used a Spectramax 384 Plus or i3x plate reader from Molecular Devices (Sunnyvale, CA, USA), and for Western blot imaging, we used the Advanced Fluorescence Imager from INTAS Science Imaging (Göttingen, Germany). Flow cytometric analyses were performed with a MACSquant Analyzer 10 and the corresponding software from Miltenyi Biotec (Teterow, Germany). For microscopy, we used a Leica DMi8 fluorescent microscope, immersion liquid (type F), and the LASX software from Leica (Wetzlar, Germany) and the comet analysis software CometScore2.0 (Rex A. Hoover, www.rexhoover.com). For determining the activity of caspase-3 or -7 activity, we applied the Caspase-Glo^®^ 3/7 assay system from Promega (Madison, WI, USA). For statistical analysis and display of data Prism 7 and Prism 9 (GraphPad, San Diego, CA, USA), for chemical structures, BioviaDraw (Dassault Systemes, Paris, France) was used, and for the design of figures, Inkscape (Free Software Foundation Inc., Boston, MA, USA) was used.

#### 3.2.2. Cell Lines and Culturing

The cell lines used for the investigations in this present study originate from lung carcinoma (A-427, LCLC-103H), pancreas carcinoma (DanG, PATU-8902, YAPC), esophageal carcinoma (Kyse-70), breast carcinoma (MCF-7), cervix adenocarcinoma (Siso), or urinary bladder carcinoma (RT-4, RT-112) and were obtained from Deutsche Sammlung von Mikroorganismen und Zellkultur (DSMZ; Braunschweig, Germany). The ovarian adenocarcinoma (A2780, A2780 cisplatin-resistant) cell lines were a gift of Dr. Julie A. Woods (Ninewells Hospital, University of Aberdeen, Aberdeen, UK). Additionally, two cell lines developed from the chronic myelogenious leukemia cell line KBM-7, namely, HAP-1 and the corresponding GPx1-knockout cell line HAP-1.KO.GPx1, were purchased from Horizon Discovery (Cambridge, UK). Unless otherwise stated, all cells were cultured under standard conditions at 37 °C and 5% CO_2_ in a humidified atmosphere and grown in RPMI 1640 medium supplemented with 10% FCS and 1% penicillin/streptomycin, except for HAP-1 cells, both native and knockout, that were cultivated with IMDM medium containing 10% FCS, 1% penicillin/streptomycin, and 1% stable glutamine. As all cell lines were adherent, a trypsin/EDTA solution was used for detachment and harvesting. Cell lines were controlled every 6 months for mycoplasma by using the Mycoplasma Detection Kit from Lonza (Basel, Switzerland). Under standard culturing conditions, levels of atmospheric oxygen were about 19%, while for hypoxia experiments oxygen was reduced to 1% by gassing with nitrogen in a HeraCell 150i incubator (ThermoFisher Scientific, Waltham, MA, USA). The levels of dissolved oxygen in cell culture media were measured with Seven2Go^TM^pro from Mettler-Toledo (Greifensee, Switzerland).

#### 3.2.3. GPx1 Enzyme Activity Assay

The potential of the pentathiepins to inhibit the GPx1 was assessed with an enzymatic assay as described previously [49]. It is based on the catalytic cycle of the enzyme coupled to an NADPH detection reaction. Here, a bovine erythrocyte GPx1 with a sequence similarity of 87% to the human homolog [50] was used for reasons of affordability. The GPx1 was treated with either vehicle (solvent DMF) or the respective pentathiepin, and the reaction started by addition of *tert*-butylhydroperoxide (*t*-BHP). For the assay, all solutions were prepared with potassium phosphate buffer (50 mM, pH 7.4, EDTA 1.1 mM, Triton-X 0.01%), except for the inhibitors and *t*-BHP, which were diluted in DMF or water, respectively. First, 180 µL of a 0.125 U/mL GPx1 solution and 30 µL of a serial dilution of the putative inhibitor dissolved in DMF were added in triplicate to the wells of a UV-transparent 96-well plate. As a negative control, only DMF was appliedat the same dilution as the inhibitor solution.. All reagents including the GPx1 solution were pipetted. Then, 30 µL of a GSH solution (2.5 mM) and 30 µL of a GR/NADPH solution (2 U/mL; 2.0 mM) were added per well. The reaction was started by adding 30 µL of a *t*-BHP solution (5.0 mM). Final concentrations were 0.075 U/mL bovine erythrocyte GPx1, 0.2 U/mL of GR, 0.25 mM of GSH, 0.2 mM of NADPH, 0.5 mM of *t*-BHP, and inhibitor concentrations between 0.05 and 12.5 μM. GPx1 activity was indirectly measured by monitoring the decrease of NADPH at λ = 340 nm for 30 min every 15 s at room temperature. The GPx1 activity of the treated samples was related to the solvent treated control and the relative activities analyzed in Prism 7 (GraphPad) via dose–response graphs and the calculation of the inflection point corresponding to the relative IC_50_ (half-maximal inhibitory concentration). The relative IC_50_ corresponds to the concentration at which half of the effect of the test compound is achieved. An absolute IC_50_, where 50% of the enzyme is inhibited, was calculated via interpolation of the sigmoidal graph.

#### 3.2.4. Enzymatic Assays for the Activity of Glutathione Reductase and Catalase

Off-target assays were conducted to assess the specificity of the pentathiepins for inhibiting the GPx1; these were the activities of GR and CAT (from bovine liver) that have previously been described [49,51]. As the baker’s yeast GR is an important component in the GPx assay, its inhibition by pentathiepins had to be excluded. A fixed pentathiepin concentration of 25 µM was tested in triplicate, following the same principle as mentioned before, by monitoring the consumption of NADPH by the GR at λ = 340 nm while GSSG is reduced back to GSH. All assay components were prepared in potassium phosphate buffer (50 mM, pH 7.4, 1.1 mM EDTA, 0.01% Triton-X solution), except for the inhibitor that was diluted in DMF. Subsequently, 180 µL of buffer was added per well followed by 30 µL of a 2.0 U/mL GR solution, 30 µL of a 2.0 mM NADPH solution, and 30 µL of a 250 µM pentathiepin solution or 30 µL of DMF as negative control. Finally, to start the reaction, 30 µL of a 2.5 mM GSSG solution was added, resulting in final concentrations of 0.2 U/mL of GR, 0.2 mM of NADPH, 25 µM of pentathiepin, and 0.25 mM of GSSG. After measuring the decrease of NADPH every 15 s for 30 min at room temperature, the relative inhibition of the GR was calculated by relating the activity of the pentathiepin-treated samples to the negative control.

The CAT activity assay is based on the principle that in the presence of H_2_O_2_ and heat, and dichromate in acetic acid is reduced to chromic acetate via perchromic acid as unstable intermediate. Here, the amount of chromic acetate is measured twice, once directly after adding H_2_O_2_ (t_0min_) and once again after the CAT (bovine origin) split the H_2_O_2_ for a period of 10 min (t_10min_) in the presence or absence of a putative inhibitor. Samples in this assay included a blank (+CAT/+DMF/−H_2_O_2_), a H_2_O_2_ standard (−CAT/+DMF/+H_2_O_2_), a negative control (+CAT/+DMF/+H_2_O_2_), and the test conditions (+CAT/+25 µM pentathiepin/+H_2_O_2_). For each condition, 3.0 mL of 0.002 mg/mL CAT in potassium phosphate buffer (50 mM, pH 7.4, EDTA 1.1 mM, Triton-X 0.01%) were prepared in a glass tube. Additionally, two tubes were prepared per condition each containing 2 mL of a reagent containing 1 part of an aqueous solution of K_2_Cr_2_O_7_ (5%) and 2 parts glacial acetic acid. Subsequently, the solvent or inhibitors were added, and the tubes placed on a shaker for 5 min. To start the reaction, 61.8 µL of a 30% H_2_O_2_ solution was added and then mixed, and immediately 1 mL was transferred to one tube containing the K_2_Cr_2_O_7_/acetic acid solution (t_0min_). The remaining 2 mL was incubated on a shaker for 10 min, and then the previous step was repeated (t_10min_). Finally, all tubes were boiled in a water bath and cooled down to room temperature, and then absorbance was measured at λ = 570 nm and the instrument was blanked with the blank solution. The difference in absorbance between t_0min_ and t_10min_ was calculated, and all samples were related to the negative control.

#### 3.2.5. MTT Assay

The effect of the pentathiepins on the viability of the cell lines was assessed via an MTT assay, performed as previously described [37]. Healthy and viable cells convert the yellow soluble 3-(4,5-dimethylthiazol-2-yl)-2,5-diphenyltetrazolium bromide to the insoluble purple corresponding formazan, which can be spectrophotometrically measured [32]. For this assay, 5000 cells (1 250 for LCLC-103H) in 0.1 mL of medium were seeded per well of a 96-well plate and incubated at standard conditions for 24 h prior to treatment. A serial dilution of the test compounds was prepared in medium, covering final concentrations from 10.0 to 0.04 µM, and 0.1 mL of the working solution was added to each well in triplicate. After 48 h, 20 µL of a 2.5 mg/mL MTT in PBS solution was pipetted into each well and further incubated for 4 h at standard conditions. Thereafter, medium was replaced with 50 µL DMSO, and the plates were put on a shaker for 5 min and subsequently read at λ = 570 nm, with a SpectraMax 384plus (Molecular Devices, Sunnyvale, CA, USA). The cell viabilities of the treatment conditions were calculated in relation to a solvent control sample. The absolute IC_50_ was calculated via nonlinear regression followed by interpolation of the dose–response graphs in Prism 7 (GraphPad, San Diego, CA, USA). The IC_90_ was derived from the IC_50_ by using the slope factor of the dose–response graph with the tool QuickCalcs from GraphPad (GraphPad Software, San Diego, CA, USA). All determined IC_50_ and all applied IC_90_ values are listed in Tables S3 and S4.

#### 3.2.6. Adapted MTT Assay to Assess the Influence of Ferroptosis

This experiment was performed similarly to that described in the previous section. To assess the importance of ferroptosis for cell death, a co-incubation of the cells with a serial dilution of the pentathiepins and ferrostatin-1 (Fer-1) as a known inhibitor of ferroptosis was performed [15,44,45]. As negative control, the cells were incubated with the serial dilution of the test compounds and an amount of DMSO corresponding to the Fer-1 treatment solution. If ferroptosis were to be involved in the cytotoxicity of the compounds, the addition of Fer-1 should protect cells from the treatment with pentathiepin. The cells were seeded in 100 µL medium per well but then treated with 50 µL of pentathiepin dilution and 50 µL of a Fer-1 or corresponding DMF working solution, which resulted in final concentrations of pentathiepins ranging from 10 to 0.04 µM and Fer-1 of either 1.5 or 6.0 µM or DMF only, respectively. The IC_50_ values were calculated as described above, and Fer-1-treated cells were set in relation to those without the additive.

#### 3.2.7. Crystal Violet Proliferation Assay

The antiproliferative effect of the pentathiepins on human cancer cell lines was analyzed by crystal violet assay based on the protocol of a previous publication [37]. The dye binds unspecifically to DNA and proteins, hence staining cells that remain after treatment. After dissolving in ethanol, the optical density was measured. Here, 1000 cells (250 for LCLC-103H) in 0.1 mL of medium were seeded per well of a 96 well plate and incubated at standard conditions for 24 h prior to treatment to allow for adhesion of the cells. A serial dilution of the test compounds was prepared in medium, covering final concentrations from 10.0 to 0.04 µM and 0.1 mL of the working solution added to each well in triplicate. Additionally, samples for each cell line were seeded and directly fixed after 24 h to measure the cell density at the beginning of treatment (t_0_). Fixation was performed by replacing medium with 0.1 mL of a solution containing 1% glutaraldehyde in Dulbecco’s buffer (0.2 g/L KCl, 0.1 g/L MgSO_4_·7 H_2_O, 1.55 g/L Na_2_HPO_4_·7 H_2_O, 0.2 g/L KH_2_HPO_4_, and 8 g/L NaCl in water) for 20 min. Plates were stored in 0.1 mL of Dulbecco’s buffer until further processing. After 96 h of treatment, the same fixation process was applied to the plates containing cells and test compounds. To stain the cells, 0.1 mL of an aqueous solution of crystal violet (0.02%) was added per well for 20 min, then discarded and the plates afterwards rinsed in clear water for 30 min. The dye was extracted in 50 µL of ethanol per well during 2 h of shaking. The plates were read at λ = 570 nm using a SpectraMax 384plus (Molecular Devices, Sunnyvale, CA, USA), and the proliferation of the treatment conditions was calculated in relation to a solvent control sample after subtracting the background value of the T_0_-plates. The GI_50_ (half-maximal growth inhibitory concentration) was calculated via nonlinear regression followed by interpolation of the dose–response graphs in Prism 7 (GraphPad, San Diego, CA, USA).

#### 3.2.8. Determination of Growth Rates and Doubling Times

The crystal violet assay was also used to determine the doubling times of the cell lines. For this purpose, 1000 cells were seeded in 0.1 mL per well of a 96-well plate and incubated for 24, 48, 72, and 96 h (ln linear growth phase) under normoxic (19% atmospheric oxygen) and hypoxic (1% atmospheric oxygen) conditions. Fixation, staining, and measurement were conducted as described in the previous paragraph. The growth rates and doubling times were calculated via the following equations:

growth rate (gr) = (ln(N_(t)_/N_(0)_))/t (1)

doubling time = (ln(2))/gr(2)

(N_(t)_ = OD at time t; N_(0)_ = OD at time 0; t = time in h).

#### 3.2.9. DCFDA-Based Flow Cytometric Assay for Detection of Reactive Oxygen Species

In order to quantify intracellular ROS, we performed a flow cytometric assay based on the ROS-sensor 2′,7′-dichlorofluorescein diacetate (DCFDA) [52,53]. For acute effects of pentathiepins on intracellular ROS levels, the cells were stained with DCFDA first and subsequently incubated with 25 µM of compound for 10 min. For monitoring a treatment of 24 or 48 h, the cells were incubated with the respective pentathiepin (IC_90_, see Table S4) and loaded with the DCFDA dye prior to analysis. Per condition, 250,000 cells were seeded in 2 mL of medium per well of a 6-well plate and allowed to adhere overnight. For measurement of acute ROS, the cells were stained with 1 mL of a 20 µM DCFDA-PBS solution for 30 min under standard incubation conditions. Afterwards, the solution was discarded, and the monolayer washed twice with 1 mL of PBS before 3 mL of medium containing 25 µM of pentathiepin was added for 10 min at 37 °C. After removing the solution and washing again with PBS, the cells were detached by using 0.5 mL of a diluted trypsin/EDTA-PBS solution (25%) and harvested with 1 mL of culture medium by carefully rinsing the wells. The cell suspension was transferred to 1.5 mL tubes, centrifuged for 5 min at 500× *g*, and washed with 1 mL of PBS. After removing the supernatant, each pellet was resuspended in 0.5 mL of PBS for measurement via the MACSquant Analyzer 10 at λ_Ex_/_Em_ = 488/530 nm. Relative ROS levels resulting from treatment with pentathiepin were calculated by relating the fluorescence intensity of treated samples to the negative control. For the experiments covering periods of 24 and 48 h, the cells were incubated with the compounds first and stained with DCFDA afterwards. To assess the influence of additional GSH, we included 3 or 30 µM GSH to the medium containing 25 µM of pentathiepin, and the protocol was followed as described.

#### 3.2.10. Apoptosis Assay Based on Annexin V-FITC and PI

The annexin V-FITC/PI assay, which discriminates between viable, early, and late apoptotic cells within a sample population, was performed according to the manufacturer’s instructions (Miltenyi Biotec, Teterow, Germany). Briefly, 125,000 cells were seeded in 2 mL per well of a 6-well plate, allowed to adhere overnight, and subsequently treated with the respective pentathiepin (IC_90_, see Table S4) diluted in 3 mL of medium. After incubation periods of 6, 24, or 48 h, the cells were harvested, washed with the provided binding buffer, and stained with the annexin V-FITC reagent for 20 min at room temperature (RT) in the dark. After another washing step, the pellet was resuspended, and PI was added immediately before flow cytometric measurement at λ_Ex_/_Em_ = 488/525 ± 50 nm for FITC and λ_Ex_/_Em_ = 488/655–730 nm for PI with the MACSquant Analyzer 10 (Miltenyi Biotec, Teterow, Germany).

#### 3.2.11. Cell Cycle Analysis Based on PI Staining

For this assay, cells were seeded and treated as for the apoptosis assay, i.e., 125,000 cells per well were incubated with the pentathiepins (IC_90_, see Table S4) for 24 or 48 h. Cells were harvested by trypsinization, collected in 1.5 mL tubes, and washed twice with PBS by centrifuging for 5 min at 500× *g* and discarding the supernatant after every step. For fixation, 500 µL of ice-cold ethanol (70%) was added dropwise under vortexing with a subsequent incubation on ice for 30 min. Then, the cells were centrifuged for 10 min at 4000 rpm and 4 °C, the supernatant was removed, and the cells were resuspended in 500 µL of PBS containing 25 µg/mL of PI and 100 µg/mL of RNase. The staining solution was incubated for 30 min, and the cell suspension was measured at λ_Ex_/_Em_ = 488/655–730 nm using a MACSquant Analyzer 10 (Miltenyi Biotec, Teterow, Germany).

#### 3.2.12. Western Blot Analysis

All Western blot analyses were performed with reagents, material, and devices purchased from Bio-Rad (Feldkirchen, Germany) if not stated otherwise. To obtain protein lysates, we seeded cells and treated them as described for the other methods. Briefly, 125,000 cells were seeded per well of a 6-well plate, incubated to adhere overnight, and then treated with pentathiepins (IC_90_, see Table S4) for 24 h. Cells were harvested by trypsinization and centrifugation for 5 min at 500× *g*. After a washing step with PBS, the cell pellet was resuspended in lysis buffer (Tris 50 mM (pH 7.4), 100 mM NaCl, 100 mM NaF, 5 mM EDTA, 0.2 mM Na_3_VO_4_, 0.1% Triton-X, and 1% protease inhibitor cocktail added immediately before use). Lysis was supported by incubating the samples for 10 min in an ultrasonic bath before centrifuging for 10 min at 18,000 × *g* and 4 °C. Protein quantification was performed via the Bradford method using ROTI^®^nanoquant reagent (Carl Roth GmbH, Karlsruhe, Germany) and bovine serum albumin as calibration standard. For electrophoretic separation, 15–40 µg of protein was loaded per well of a polyacrylamide gel (Mini- or Midi-Protean^®^ TGX stain-free gel, Bio-Rad, Feldkirchen, Germany), and subsequent blotting was performed with Trans-Blot^®^Turbo™TransferPack Mini or Midi PVDF membranes from Bio-Rad (Feldkirchen, Germany). The membranes were blocked with a 10% non-fat milk powder solution in Tris-buffered saline (TBS; 2.42 g/L Tris, 8.48 g/L NaCl in water) plus 0.5% Tween-20 (TBST) for 1 h at RT. Incubation with primary antibodies took place over night at 4 °C in a 1:1000 dilution in TBST containing BSA (1%). The secondary antibodies conjugated to horse-radish peroxidase were diluted 1:10,000 in TBST and 1% BSA, and incubated for 4 h at RT. A detection step followed, applying Clarity™Western ECL substrate with subsequent imaging via the INTAS Advanced Fluorescence Imager. Between all incubation steps, the blots were washed three times for 10 min with TBST. The bands were quantified with the ImageLab software and normalized using the total protein quantification of the TGX stain-free gel system from Bio-Rad [38,39,40].

#### 3.2.13. Plasmid Cleavage Assay

This assay was used to assess the cleaving ability of the pentathiepins in a cell-free setting and on the basis of procedures described in [54]. Plasmids occur in three different conformations with distinct electrophoretic mobilities that can finally be separated visualized via agarose gel electrophoresis. For each condition, 0.3 µg of pBR322 plasmid DNA was incubated with either the pentathiepin or a corresponding amount of the solvent acetonitrile. Incubations were performed in sodium phosphate buffer (50 mM) with or without GSH for 20 h at 37 °C in a water bath. Afterwards, the samples were separated by using an agarose gel (1%) run at 80 V for 2 h. Resulting bands were stained with GelRed^®^ (Sigma-Aldrich, Taufkirchen, Germany),) and imaged and quantified via Gel-Doc EZ Imager and Image Lab (Bio-Rad, Feldkirchen, Germany), respectively. To assess the cleavage ability of the pentathiepins, we incubated the plasmid with either 5 or 25 µM of compound at a fixed concentration of GSH (2 mM). In another experiment, the amount of GSH varied between 1 µM and 10 mM, while the pentathiepin concentration was 5 µM. In contrast to the aforementioned settings where a buffer pH of 7.1 was applied, in a third attempt, the influence of different pH on the cleavage outcome was analyzed by adjusting the buffer pH to 5.1, 6.1, or 8.1 while using fixed concentrations of both test compound and GSH. Another attempt to gain insights into the cleaving mechanism was made by adding either catalase, superoxide dismutase (100 µg/mL each), or both to 5 µM pentathiepin with 2 mM GSH.

#### 3.2.14. Alkaline Comet Assay

With this assay, the intensity of DNA damage can be assessed on the level of a single cell, in case of the alkaline version detecting both single and double strand breaks as well as alkali-labile sites. The method presented here is an adaptation of the protocol published by Olive and Banath [55] and based on the distinct electrophoretic mobility of damaged and intact genomic DNA. The latter has a low mobility; does thus not migrate in the electric field; and remains in a nucleoid shape after lysis, referred to as the comet head. In contrast, when strand breaks are created, free charged ends emerge and increase the electrophoretic mobility, prompting DNA segments to drift away from the comet head, thereby forming the so-called comet tail. As descriptor of still intact genomic DNA, the percentage of DNA in the comet head was selected. Per condition, 50,000 cells of either Siso, HAP-1, or HAP-1.KO.GPx1 were diluted in 1 mL of PBS and subsequently treated with either a 1% DMF aqueous solution as negative control, 20 µM of H_2_O_2_ as positive control, or 25 µM of pentathiepin for 15 min on ice to impair intracellular DNA repair mechanisms. Afterwards, 400 µL of the cell suspension was blended with 1.2 mL of a 1% low melting point agarose solution (40 °C) and smoothly distributed on agarose-precoated glass slides. Polymerization was finished after 3 min at RT, and the slides were horizontally submerged in alkaline lysis buffer (1.2 M NaCl, 0.1% *N*-lauryl-sarcosinate, 0.1 M Na-EDTA, 0.26 M NaOH) before storing them at 4 °C overnight. The slides were then rinsed three times in rinse/electrophoresis buffer (0.002 M Na-EDTA, 0.03 M NaOH) before electrophoretic separation was performed for 25 min at 0.6 V/cm. A neutralization step by adding distilled water was followed by staining the slides with 250 µL of an aqueous 10 µg/mL PI solution for 20 min. For visualization and image capturing, a DMi8 fluorescent microscope and LASX software (Leica Microsystems, Wetzlar, Germany) were used, and the comets scored with the help of CometScore2.0 (rexhoover.com). Per condition and replicate, at least 100 comets were analyzed to calculate a mean level of damage.

#### 3.2.15. Fluorescence Microscopy

For visualization of the putative trackable pentathiepin **1**, a DMi8 (Leica Microsystems, Wetzlar, Germany) and the corresponding LASX software were used. Siso cells were seeded on cover slips at a density of 125,000 cells in 2 mL per well of a 6-well plate, incubated overnight for adherence, and then treated with compound **1** (IC_90_, see Table S4) or solvent. After 24 h, the medium was removed, and the cells were washed once with PBS and placed on microscopic glass slides with mounting medium. The cover slips were fixed with sealant, immersion oil was applied, and the cells were subsequently visualized. The pentathiepin distribution was imaged with the 63× magnification objective in the DAPI channel (λ_ex_ = 325–407 nm, λ_em_ = 461 nm) and the autofluorescence of the cells in the FITC channel (λ_ex_ = 488 nm, λ_em_ = 525 nm).

#### 3.2.16. Luminescent Caspase Activity Assay

To study the activation of the effector caspases -3 and -7, we performed a luminescence-based assay according to the manufacturer’s instructions (Caspase-Glo, Promega, Walldorf, Germany). Briefly, 5000 cells were seeded in 0.1 mL per well of a 96-well plate and allowed to adhere overnight. The cells were treated with either 0.2% DMF, 0.5 µM of doxorubicin, or the respective pentathiepin (IC_90_, see Table S4) and incubated for 24 h at standard conditions. Afterwards, the standard protocol was followed, including a 30 min incubation time before measurement of luminescent signals by using the Spectramax i3x (Molecular Devices, Sunnyvale, CA, USA). After subtraction of the background luminescence the positive control and treatment conditions were related to the negative control.

#### 3.2.17. Statistic Evaluation and Correlation Analysis

Data and diagrams were prepared with Prism 7 (Version 7.0) and 9 (Version 9.1) from GraphPad Software Inc. (San Diego, CA, USA) and presented as means with standard deviations (SD) of at least three independent experiments if not stated otherwise. For the display of dose–response graphs in the GPx1 assay, the 95% confidence interval was included instead of the SD. Assessment of statistical significance was performed via ANOVA (analysis of variation) coupled with a Dunnett’s or Tukey’s multiple comparisons test and significance levels expressed as * *p* < 0.05, ** *p* < 0.01, *** *p* < 0.001, or **** *p* < 0.0001.

To analyze a putative correlation between potency and cell doubling times, we used the Pearson correlation calculation of Prism 7 or 9 (GraphPad Software, San Diego, CA, USA). It was assessed to what extent two variables vary together, in this case X was either the IC_50_ from MTT or GI_50_ from crystal violet assay and Y was the doubling time of the respective cell line. Results are output as correlation efficient r with the corresponding *p*-value as level of significance. For interpretation of the *r*-values, we applied a common scale: 0.0–0.1 negligible, >0.1–<0.4 weak, 0.4–<0.7 moderate, 0.7–<0.9 moderate, >0.9 strong [36].

## 4. Conclusions

Pentathiepins represent a class of sulfur-containing small molecules that induce a broad range of biological effects. Recently, potent and specific inhibition of the glutathione peroxidase 1, induction of reactive oxygen species, apoptosis, and the depolarization of the mitochondrial membrane have been found to be induced by such compounds. Such biological features render pentathiepins a promising class of drugs for the application as anticancer agents. However, a comprehensive study with a range of biological assays in different human cancer cell lines is lacking. Hence, the aim of the present work was to provide a comprehensive insight into the in vitro effects of pentathiepins in various cancer cell lines, as well as to identify possible structure–activity relationships.

Six new pentathiepins were synthesized that possess strong cytotoxicity in a panel of 14 human cancer cell lines with IC_50_ values in the low micromolar range. Hypoxia decreased these effects, except for pentathiepin **1**, indicating that the availability of oxygen is necessary for pentathiepins **2**–**6** to unfold their activity.

The cytotoxicity of the compounds is believed to result chiefly from ROS-mediated cleavage of DNA in the presence of GSH. Here, a connection between the induction of ROS and the ability to cleave DNA was found. Pentathiepins **2**, **3**, **4**, and **5** caused intracellular oxidative stress and at the same time the highest rates of DNA damage. Cell cycle arrest in the G_2_/M phase for these four compounds mirrors what is known for other DNA damaging anticancer agents, such as bleomycin [46]. On the other hand, for pentathiepin **1**, we detected a lower potential to cleave DNA but also observed by fluorescence microscopy that this compound enters cells but not their nuclei.

With regards to the mode of cell death, strong evidence for the induction of apoptosis was found, but not for all endpoints in all cell lines. The externalization of phosphatidylserine was accompanied by the detection of cleaved PARP1 and the activation of caspase-3 and -7 in the two HAP-1 leukemia cell lines, but these attributes were less apparent in the A2780 and Siso solid tumor cell lines.

With regards to the previously discovered ability of pentathiepins to inhibit the GPx1, we confirmed this for the isolated enzyme from bovine erythrocytes with the new compounds **1**–**5**. However, evidence to date indicates that GPx1 inhibition is not required for the cytotoxic effects of pentathiepins. We base this conclusion on the observation that compound **6** is cytotoxic without being a potent GPx1 inhibitor. Moreover, no significant differences were detected between the GPx1 knockout variant and the parental cell line HAP-1 throughout the majority of biological assays performed herein.

While it is too early to state that pentathiepins have therapeutic potential as anticancer drugs, progress has been made to chemically optimize their structures towards biological activity. Finally, the finding that the six pentathiepins show structure-dependent biological activity offers the possibility to modulate biological effects by fusion of specific scaffolds to the pentathiepin ring.

## Figures and Tables

**Table 1:** Relative (Rel.) and absolute (Abs.) IC_50_ values of pentathiepins **1**–**6** in µM with corresponding CI 95%, SD, and residual bovine erythrocyte GPx1 enzyme activity at the highest tested concentration of 12.5 µM of n ≥ 3 independent experiments.

Compound	Rel. IC_50_ (CI 95%)	Abs. IC_50_ (SD)	Residual Activity (%)
**1**	0.92 (n.d.–2.10)	1.49 (0.76)	16.4
**2**	2.28 (1.52–13.74)	3.25 (0.95)	27.5
**3**	0.56 (0.24–0.80)	0.91 (0.33)	29.0
**4**	0.60 (0.32–0.84)	0.75 (0.27)	27.9
**5**	0.63 (0.50–0.75)	1.17 (0.20)	28.7
**6**	>12.5	>12.5	53.3

**Table 2:** Summary of the biological effects of pentathiepins **1**–**6** from the present study and (**c**) from the first generation of compounds [12] ^1^.

Investigation	1	2	3	4	5	6	(c)
GPx1 inhibition	++	+	++	++	++	0	+
GR/CAT inhibition	0	0	0	0	0	0	0
Cytotoxicity	++	+	++	++	++	+	+
Antiproliferative effect	++	+	++	++	++	+	+
ROS induction	0	++	++	++	++	+	+
DNA cleavage	+	++	++	++	++	+	+
Comet formation	+	+	++	++	++	+	+
Apoptosis	+	+	++	+	++	+	+
Ferroptosis	0	0	0	0	0	0	0
G_0_/G_1_ arrest	+	0	0	0	0	+	n.d.
G_2_/M arrest	0	+	+	+	+	0	n.d.
Morphological changes	0	+	++	++	++	+	+

^1^ no impact (0), weak impact (+), strong impact (++), no data (n.d.).

## Data Availability

Primary data can be found in the Supplementary Materials.

## Supplementary Materials

The following are available online at https://www.mdpi.com/article/10.3390/ijms22147631/s1.

## References

[1] Konstantinova L.S., Rakitin O.A., Rees C.W. (2004). Pentathiepins. Chem. Rev..

[2] Feher F., Langer M. (1971). Contribution to chemistry of sulfur. 104: Synthesis of pentathiepin and benzopentathiepin. Tetrahedron Lett..

[3] Davidson B.S., Molinski T.F., Barrows L.R., Ireland C.M. (1991). Varacin—A novel benzopentathiepin from Lissoclinum-Vareau that is cytotoxic toward a human colon-tumor. J. Am. Chem. Soc..

[4] Asquith C.R.M., Laitinen T., Konstantinova L.S., Tizzard G., Poso A., Rakitin O.A., Hofmann-Lehmann R., Hilton S.T. (2019). Investigation of the pentathiepin functionality as an inhibitor of feline immunodeficiency virus (FIV) via a potential zinc wjection mechanism, as a model for HIV infection. ChemMedChem.

[5] Litaudon M., Trigalo F., Martin M.T., Frappier F., Guyot M. (1994). Lissoclinotoxins—Antibiotic polysulfur derivatives from the Tunicate Lissoclinum-Perforatum—Revised structure of Lissoclinotoxin-A. Tetrahedron.

[6] Chatterji T., Gates K.S. (1998). DNA cleavage by 7-methylbenzopentathiepin: A simple analog of the antitumor antibiotic varacin. Bioorg. Med. Chem. Lett..

[7] Mahendran A., Ghogare A.A., Bittman R., Arthur G., Greer A. (2016). Synthesis and antiproliferative properties of a new ceramide analog of varacin. Chem. Phys. Lipids.

[8] Compagnone R.S., Faulkner D.J., Carte B.K., Chan G., Freyer A., Hemling M.E., Hofmann G.A., Mattern M.R. (1994). Pentathiepins and trithianes from 2 Lissoclinum Species and a Eudistoma Sp—Inhibitors of protein-kinase-C. Tetrahedron.

[9] Baguley T.D., Nairn A.C., Lombroso P.J., Ellman J.A. (2015). Synthesis of benzopentathiepin analogs and their evaluation as inhibitors of the phosphatase STEP. Bioorg. Med. Chem. Lett..

[10] Xu J., Chatterjee M., Baguley T.D., Brouillette J., Kurup P., Ghosh D., Kanyo J., Zhang Y., Seyb K., Ononenyi C. (2014). Inhibitor of the tyrosine phosphatase STEP reverses cognitive deficits in a mouse model of Alzheimer’s disease. PLoS Biol..

[11] Zakharenko A., Khomenko T., Zhukova S., Koval O., Zakharova O., Anarbaev R., Lebedeva N., Korchagina D., Komarova N., Vasiliev V. (2015). Synthesis and biological evaluation of novel tyrosyl-DNA phosphodiesterase 1 inhibitors with a benzopentathiepine moiety. Bioorg. Med. Chem..

[12] Behnisch-Cornwell S., Bandaru S.S.M., Napierkowski M., Wolff L., Zubair M., Urbainsky C., Lillig C., Schulzke C., Bednarski P.J. (2020). Pentathiepins: A novel class of glutathione peroxidase 1 inhibitors that induce oxidative stress, loss of mitochondrial membrane potential and apoptosis in human cancer cells. ChemMedChem.

[13] Flohe L., Gunzler W.A. (1973). Gsh Peroxidase. H-S Z Physiol. Chem..

[14] Lubos E., Loscalzo J., Handy D.E. (2011). Glutathione peroxidase-1 in health and disease: From molecular mechanisms to therapeutic opportunities. Antioxid. Redox Signal..

[15] Dixon S.J., Lemberg K.M., Lamprecht M.R., Skouta R., Zaitsev E.M., Gleason C.E., Patel D.N., Bauer A.J., Cantley A.M., Yang W.S. (2012). Ferroptosis: An iron-dependent form of nonapoptotic cell death. Cell.

[16] Yang W.S., SriRamaratnam R., Welsch M.E., Shimada K., Skouta R., Viswanathan V.S., Cheah J.H., Clemons P.A., Shamji A.F., Clish C.B. (2014). Regulation of ferroptotic cancer cell death by GPX4. Cell.

[17] Lee J.R., Roh J.-L., Lee S.M., Park Y., Cho K.-J., Choi S.-H., Nam S.Y., Kim S.Y. (2017). Overexpression of glutathione peroxidase 1 predicts poor prognosis in oral squamous cell carcinoma. J. Cancer Res. Clin. Oncol.

[18] Wieczorek E., Jablonowski Z., Tomasik B., Gromadzinska J., Jablonska E., Konecki T., Fendler W., Sosnowski M., Wasowicz W., Reszka E. (2017). Different gene expression and activity pattern of antioxidant enzymes in bladder cancer. Anticancer Res..

[19] Cullen J.J., Mitros F.A., Oberley L.W. (2003). Expression of antioxidant enzymes in diseases of the human pancreas: Another link between chronic pancreatitis and pancreatic cancer. Pancreas.

[20] Gladyshev V.N., Factor V.M., Housseau F., Hatfield D.L. (1998). Contrasting patterns of regulation of the antioxidant selenoproteins, thioredoxin reductase, and glutathione peroxidase, in cancer cells. Biochem. Biophys. Res. Commun..

[21] Schulz R., Emmrich T., Lemmerhirt H., Leffler U., Sydow K., Hirt C., Kiefer T., Link A., Bednarski P.J. (2012). Identification of a glutathione peroxidase inhibitor that reverses resistance to anticancer drugs in human B-cell lymphoma cell lines. Bioorganic Med. Chem. Lett..

[22] Behnisch-Cornwell S., Wolff L., Bednarski P.J. (2020). The effect of Gglutathione peroxidase-1 knockout on anticancer drug sensitivities and reactive oxygen species in haploid HAP-1 cells. Antioxidant.

[23] Chaudiere J., Wilhelmsen E.C., Tappel A.L. (1984). Mechanism of selenium-glutathione peroxidase and its inhibition by mercaptocarboxylic acids and other mercaptans. J. Biol. Chem..

[24] Chatterji T., Gates K.S. (2003). Reaction of thiols with 7-methylbenzopentathiepin. Bioorganic Med. Chem. Lett..

[25] Tatu M.-L., Georgescu E., Boscornea C., Popa M.-M., Ungureanu E.-M. (2017). Synthesis and fluorescence of new 3-biphenylpyrrolo[1,2-c]pyrimidines. Arab. J. Chem..

[26] DiPalma J.R., Thayer W.S. (1991). Use of niacin as a drug. Annu. Rev. Nutr..

[27] Rathi A.K., Syed R., Shin H.-S., Patel R.V. (2016). Piperazine derivatives for therapeutic use: A patent review (2010-present). Expert Opin. Ther. Pat..

[28] Böhm H.-J., Banner D., Bendels S., Kansy M., Kuhn B., Müller K., Obst-Sander U., Stahl M. (2004). Fluorine in medicinal chemistry. ChemBioChem.

[29] Ojima I. (2004). Use of Fluorine in the medicinal chemistry and chemical biology of bioactive compounds—A case study on fluorinated taxane anticancer agents. ChemBioChem.

[30] Kirk K.L., Filler R. (1996). Recent advances in the biomedicinal chemistry of fluorine-containing compounds. Biomedical Frontiers of Fluorine Chemistry.

[31] Lange P.P., Bogdan A.R., James K. (2012). A New flow methodology for the expedient synthesis of drug-like 3-aminoindolizines. Adv. Synth. Catal..

[32] Mosmann T. (1983). Rapid colorimetric assay for cellular growth and survival: Application to proliferation and cytotoxicity assays. J. Immunol. Methods.

[33] Slater T.F., Sawyer B., Straeuli U. (1963). Studies on succinate-tetrazolium reductase systems. III. Points of coupling of four different tetrazolium salts. Biochim. Biophys. Acta.

[34] Gillies R.J., Didier N., Denton M. (1986). Determination of cell number in monolayer cultures. Anal. Biochem..

[35] Swietach P., Vaughan-Jones R.D., Harris A.L., Hulikova A. (2014). The chemistry, physiology and pathology of pH in cancer. Philos. Trans. R. Soc. B.

[36] Schober P., Boer C., Schwarte L.A. (2018). Correlation coefficients: Appropriate use and interpretation. Anesth. Analg..

[37] Bracht K., Boubakari, Grünert R., Bednarski P.J. (2006). Correlations between the activities of 19 anti-tumor agents and the intracellular glutathione concentrations in a panel of 14 human cancer cell lines: Comparisons with the National Cancer Institute data. Anticancer Drugs.

[38] Rivero-Gutierrez B., Anzola A., Martinez-Augustin O., de Medina F.S. (2014). Stain-free detection as loading control alternative to Ponceau and housekeeping protein immunodetection in Western blotting. Anal. Biochem..

[39] Colella A.D., Chegenii N., Tea M.N., Gibbins I.L., Williams K.A., Chataway T.K. (2012). Comparison of Stain-Free gels with traditional immunoblot loading control methodology. Anal. Biochem..

[40] Gurtler A., Kunz N., Gomolka M., Hornhardt S., Friedl A.A., McDonald K., Kohn J.E., Posch A. (2013). Stain-Free technology as a normalization tool in Western blot analysis. Anal. Biochem..

[41] Lee A.H.F., Chan A.S.C., Li T.H. (2002). Acid-accelerated DNA-cleaving activities of antitumor antibiotic varacin. Chem. Commun..

[42] Lee A.H., Chen J., Liu D., Leung T.Y., Chan A.S., Li T. (2002). Acid-promoted DNA-cleaving activities and total synthesis of varacin C. J. Am. Chem. Soc..

[43] Martin S.J., Reutelingsperger C.P., McGahon A.J., Rader J.A., van Schie R.C., LaFace D.M., Green D.R. (1995). Early redistribution of plasma membrane phosphatidylserine is a general feature of apoptosis regardless of the initiating stimulus: Inhibition by overexpression of Bcl-2 and Abl. J. Exp. Med..

[44] Skouta R., Dixon S.J., Wang J., Dunn D.E., Orman M., Shimada K., Rosenberg P.A., Lo D.C., Weinberg J.M., Linkermann A. (2014). Ferrostatins inhibit oxidative lipid damage and cell death in diverse disease models. J. Am. Chem Soc..

[45] Sagasser J., Ma B.N., Baecker D., Salcher S., Hermann M., Lamprecht J., Angerer S., Obexer P., Kircher B., Gust R. (2019). A new approach in cancer treatment: Discovery of chlorido[N,N′-disalicylidene-1,2-phenylenediamine]iron(III) complexes as ferroptosis inducers. J. Med. Chem..

[46] Chabner B., Chabner B., Collins J.M. (1990). Bleomycin. Cancer Chemotherapy: Principles and Practice.

[47] Mettey Y., Gompel M., Thomas V., Garnier M., Leost M., Ceballos-Picot I., Noble M., Endicott J., Vierfond J.-M., Meijer L. (2003). Aloisines, a new family of CDK/GSK-3 inhibitors. SAR study, crystal structure in complex with CDK2, enzyme selectivity, and cellular effects. J. Med. Chem..

[48] Ritzén A., Sindet R., Hentzer M., Svendsen N., Brodbeck R.M., Bundgaard C. (2009). Discovery of a potent and brain penetrant mGluR5 positive allosteric modulator. Bioorganic Med. Chem. Lett..

[49] Lemmerhirt H., Behnisch S., Bodtke A., Lillig C.H., Pazderova L., Kasparkova J., Brabec V., Bednarski P.J. (2018). Effects of cytotoxic cis- and trans-diammine monochlorido platinum(II) complexes on selenium-dependent redox enzymes and DNA. J. Inorg. Biochem..

[50] Wilde F., Chamseddin C., Lemmerhirt H., Bednarski P.J., Jira T., Link A. (2014). Evaluation of (S)- and (R)-misonidazole as GPx inhibitors: Synthesis, characterization including circular dichroism and in vitro testing on bovine GPx-1. Arch. Pharm..

[51] Sinha A.K. (1972). Colorimetric assay of catalase. Anal. Biochem..

[52] Wang H., Joseph J.A. (1999). Quantifying cellular oxidative stress by dichlorofluorescein assay using microplate reader. Free Radical. Biol. Med..

[53] Amer J., Goldfarb A., Fibach E. (2003). Flow cytometric measurement of reactive oxygen species production by normal and thalassaemic red blood cells. Eur. J. Haematol..

[54] Behroozi S.J., Kim W., Dannaldson J., Gates K.S. (1996). 1,2-Dithiolan-3-one 1-oxides: A class of thiol-activated DNA-cleaving agents that are structurally related to the natural product leinamycin. Biochemistry.

[55] Olive P.L., Banath J.P. (2006). The comet assay: A method to measure DNA damage in individual cells. Nat. Protoc..

