# Supplementary material for: Comprehensive Evaluation of Biological Effects of Pentathiepins on Various Human Cancer Cell Lines and Insights into Their Mode of Action
Source: Int J Mol Sci. 2021 Jul 16;22(14):7631. doi: 10.3390/ijms22147631 (PMC8305076; doi:10.3390/ijms22147631)
Supplement: Supplementary file 1 [file ijms-22-07631-s001.zip › ijms-1223985-supplementary.pdf]

Supplemental Materials

Comprehensive evaluation of biological effects of pentathiepins on various human cancer cell lines and insights into their mode of action

Lisa Wolff, Siva Sankar Murthy Bandaru, Elias Eger, Hoai-Nhi Lam, Martin Napierkowski, Daniel Baecker, Carola Schulzke and Patrick J. Bednarski

<u>Supporting Information</u>

Contains: 51 Figures and 4 Tables

Chemistry

HPLC analysis of compounds 1-6

RP-HPLC analyses were performed with a Merck-Hitachi LaChrom 7000 instrument equipped with a Merck Chromolith SpeedROD RP-18e column (4.6 x 50 mm) and maintained at 30 °C. Void time was determined as 0.72 min. For compound **1** 20 µL of a 35 µM solution were injected and eluted with a solvent of 70 % acetonitrile/30 % phosphate buffer (10 mM, pH 2) at a flow rate of 1.25 mL/min. Of compounds **2**, **3**, **4** and **6** samples of 25 µL of a 200 µM solution were injected and eluted with a solvent of 80 % acetonitrile/20 % water at a flow rate of 1.25 mL/min. Pentathiepin **5** was eluted with a solvent of 75 % acetonitrile/25 % water at a flow rate of 1.25 mL/min. Detection was performed between the wavelengths of 210 and 500 nm.

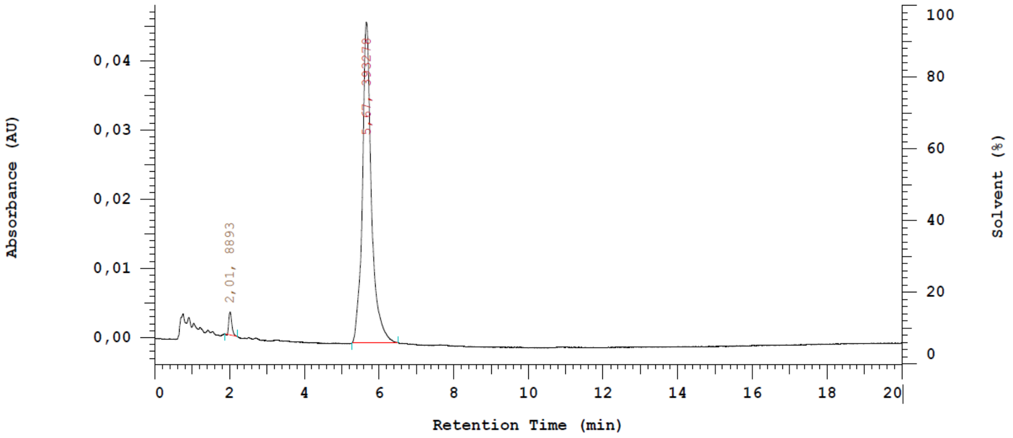

Figure S1: RP-HPLC chromatogram of compound **1**, t_R=5.67 min, detected at λ=250 nm.

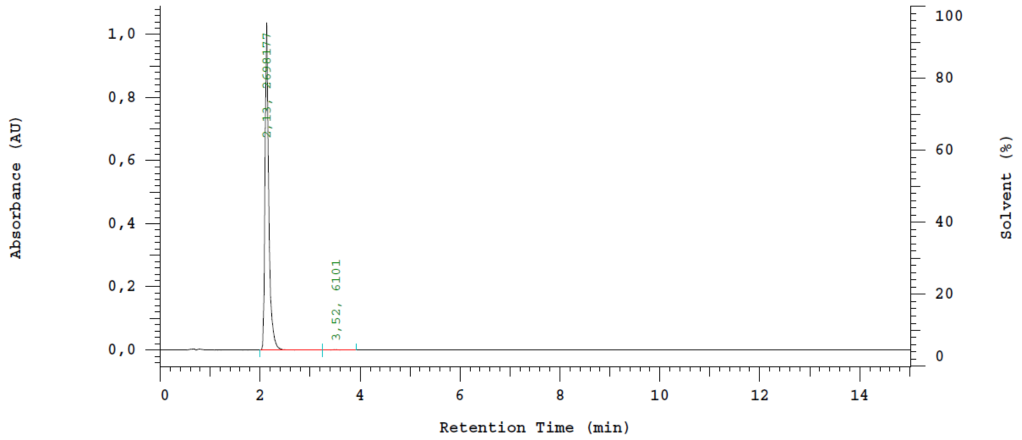

Figure S2: RP-HPLC chromatogram of compound **2**, t_R=2.13 min, detected at λ=250 nm.

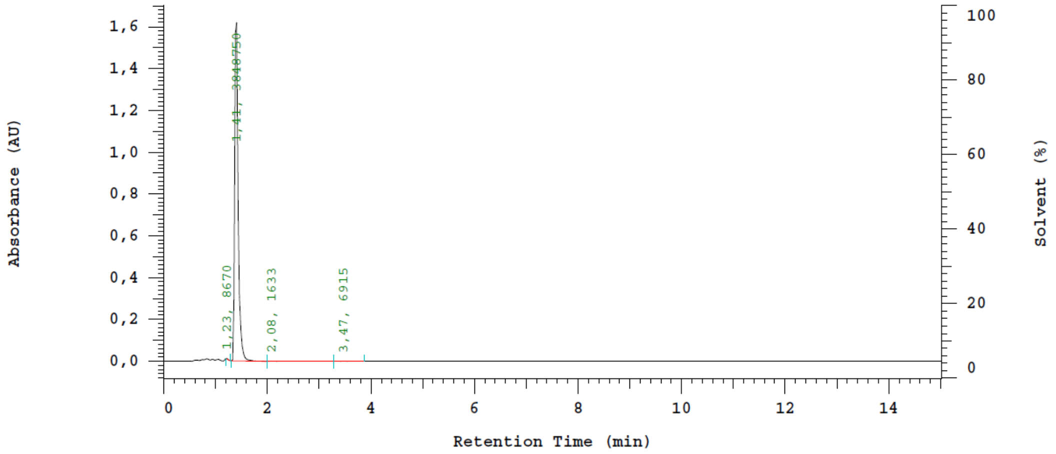

Figure S3: RP-HPLC chromatogram of compound **3**, t_R=1.41 min, detected at λ=250 nm.

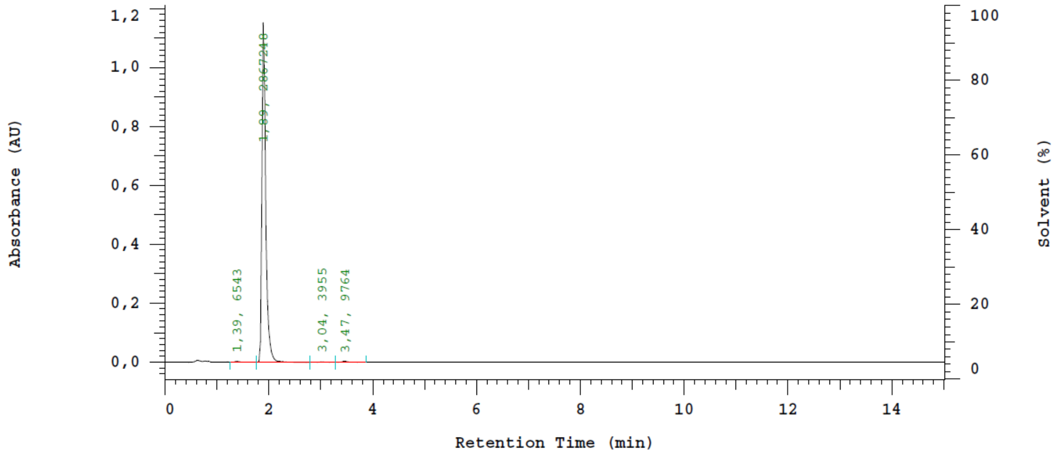

Figure S4: RP-HPLC chromatogram of compound **4**, t_R=1.89 min, detected at λ=250 nm.

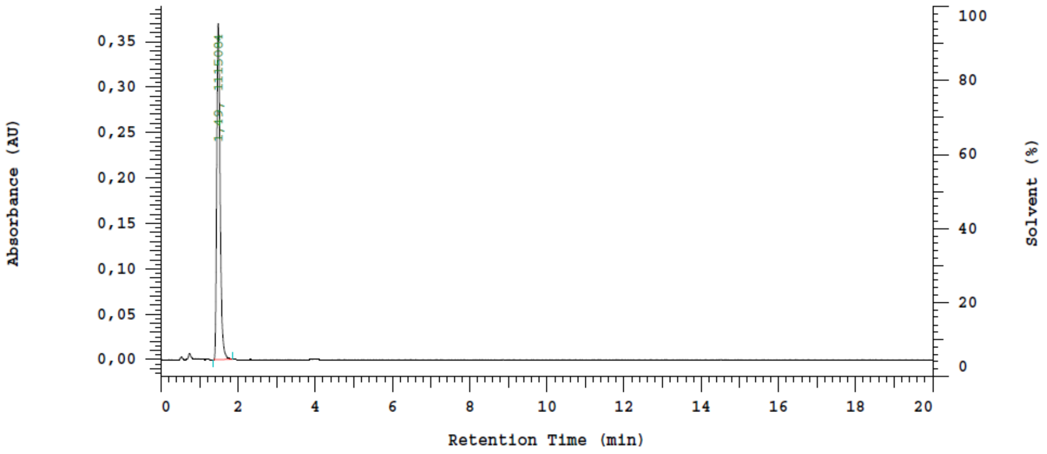

Figure S5: RP-HPLC chromatogram of compound **5**, t_R=1.49 min, detected at λ=250 nm.

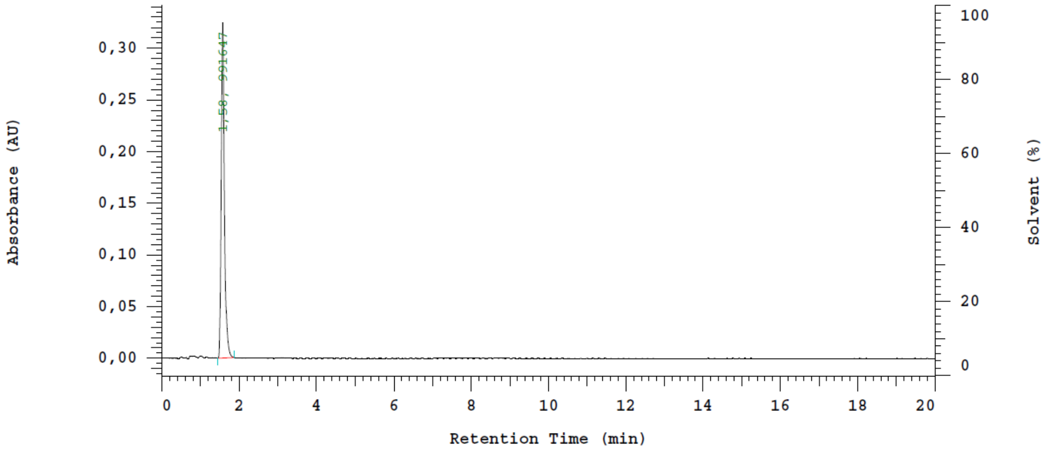

Figure S6: RP-HPLC chromatogram of compound **6**, t_R=1.58 min, detected at λ=250 nm.

^{1}H and ^{13}C NMR spectra

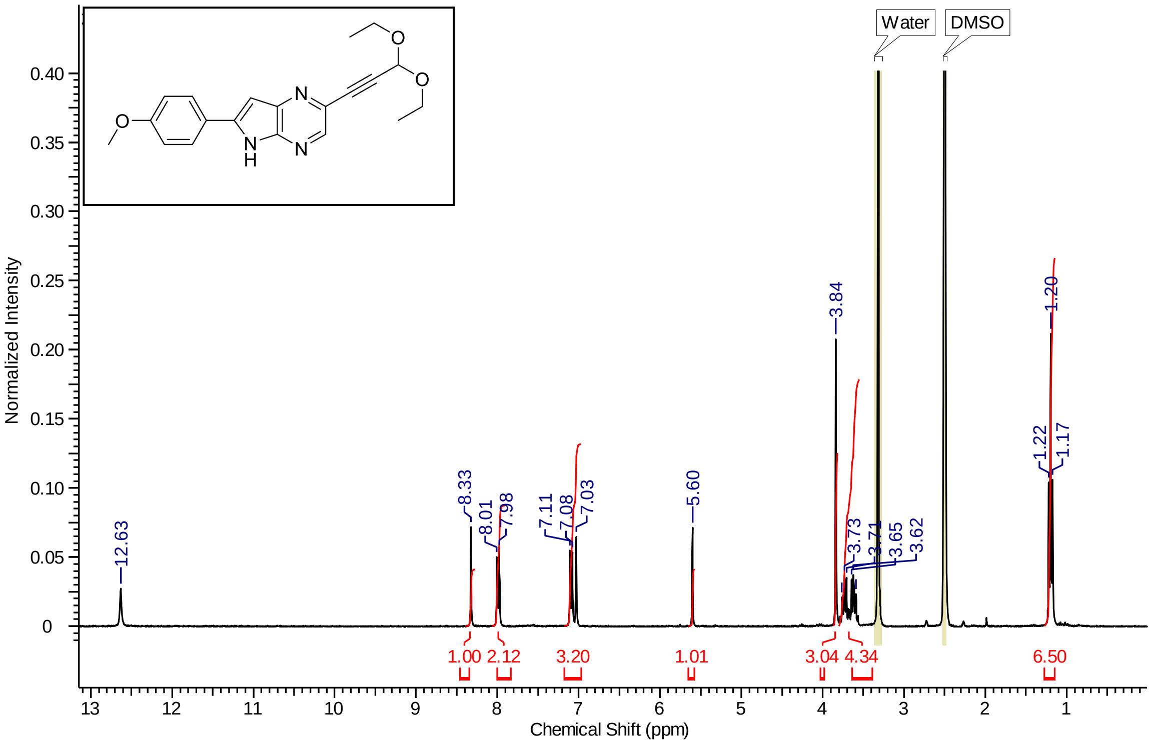

Figure S7: ^{1}H NMR spectrum of IV.

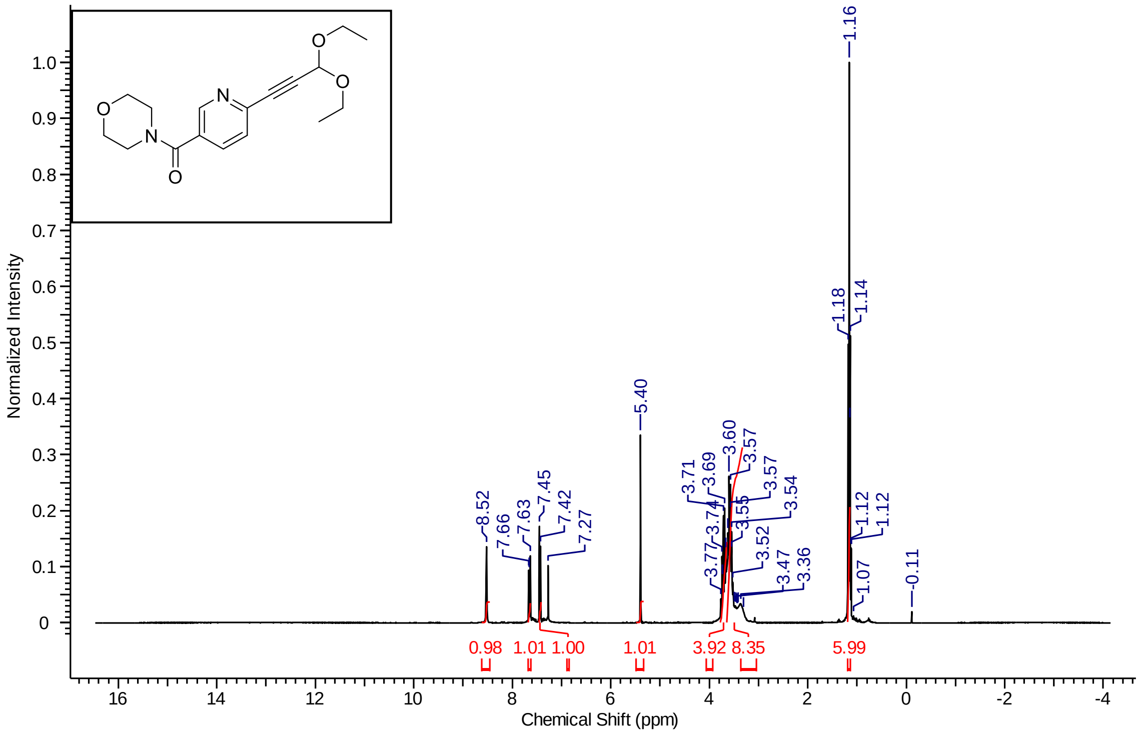

Figure S8: ^{1}H NMR spectrum of Y b.

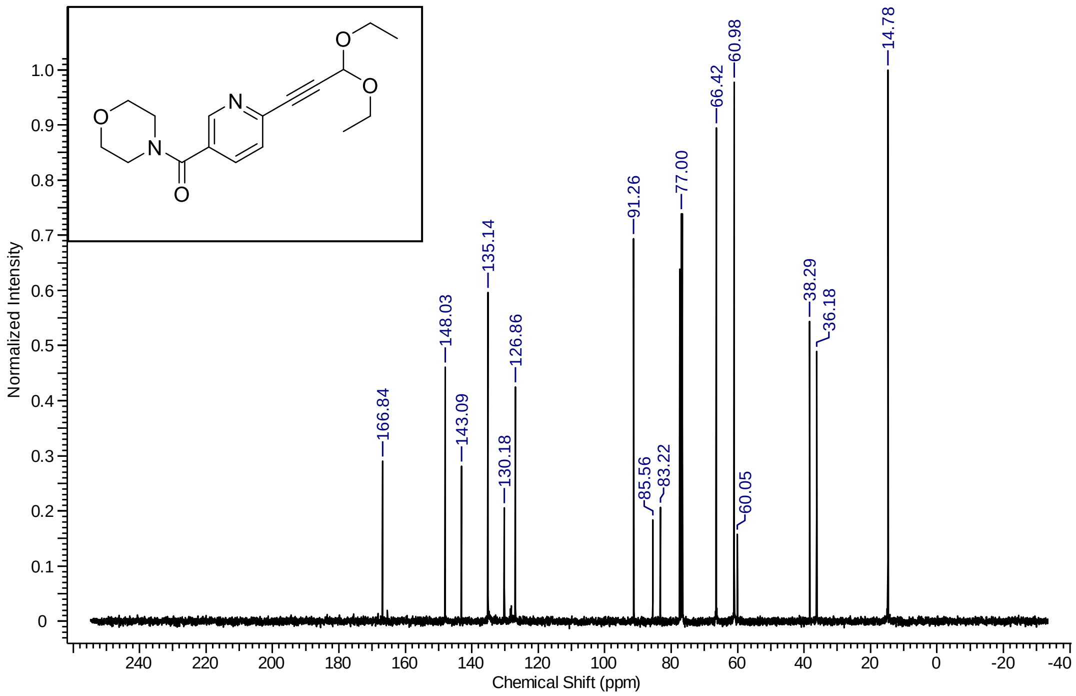

Figure S9: ^{13}C NMR spectrum of Y b.

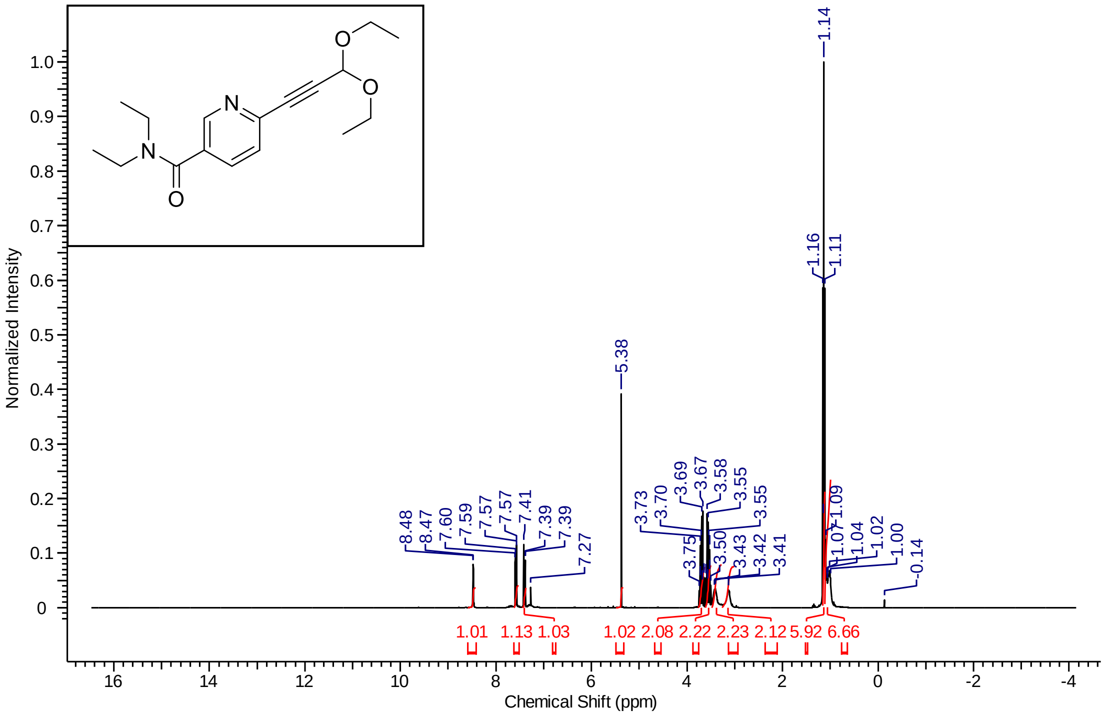

Figure S10: ^{1}H NMR spectrum of Y c.

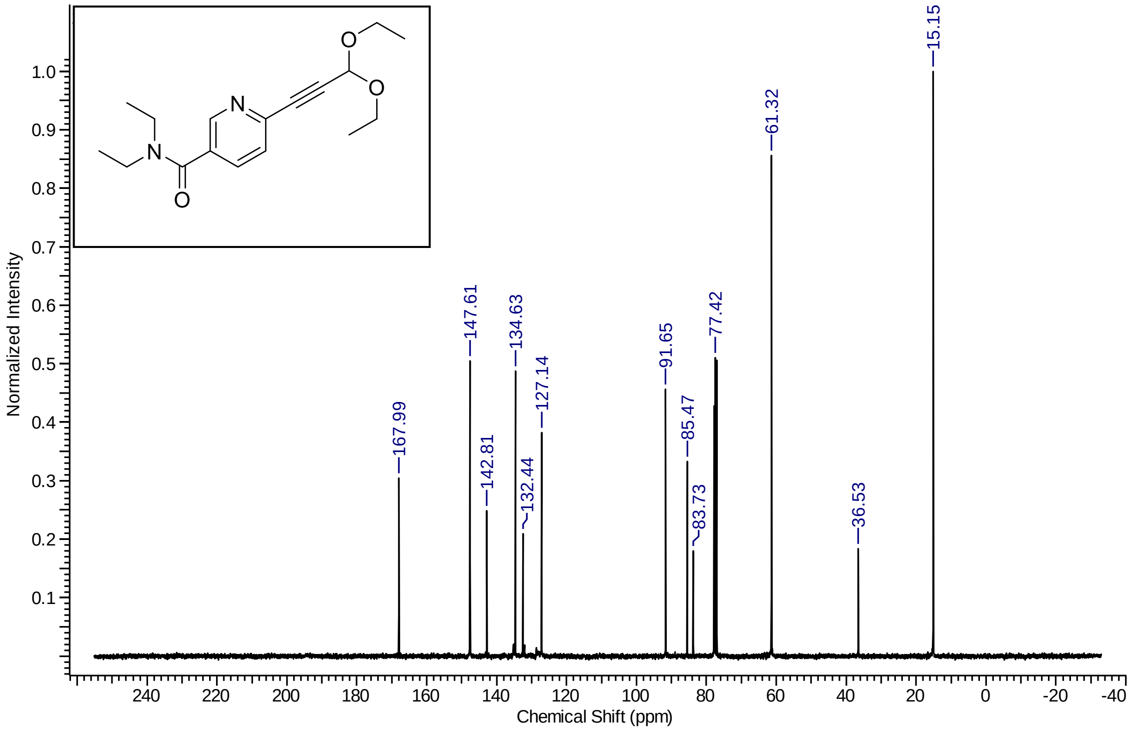

Figure S11: ^{13}C NMR spectrum of Y c.

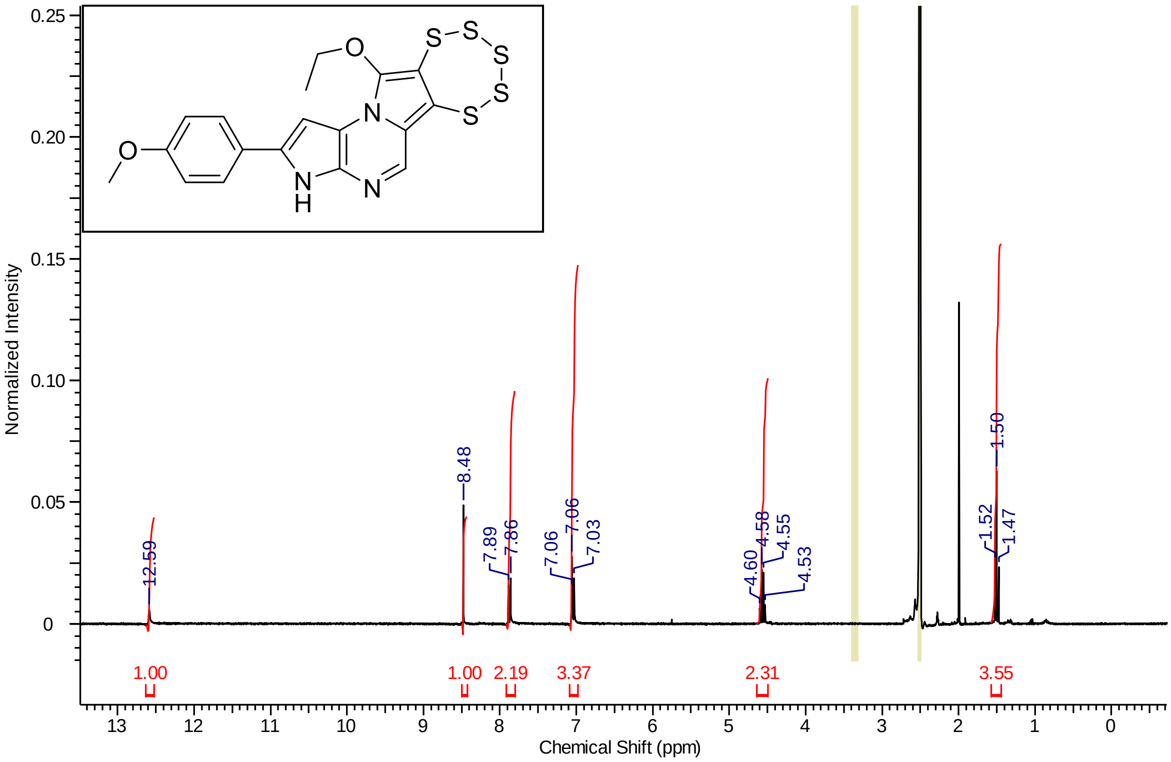

Figure S12: ^{1}H NMR spectrum of **1**.

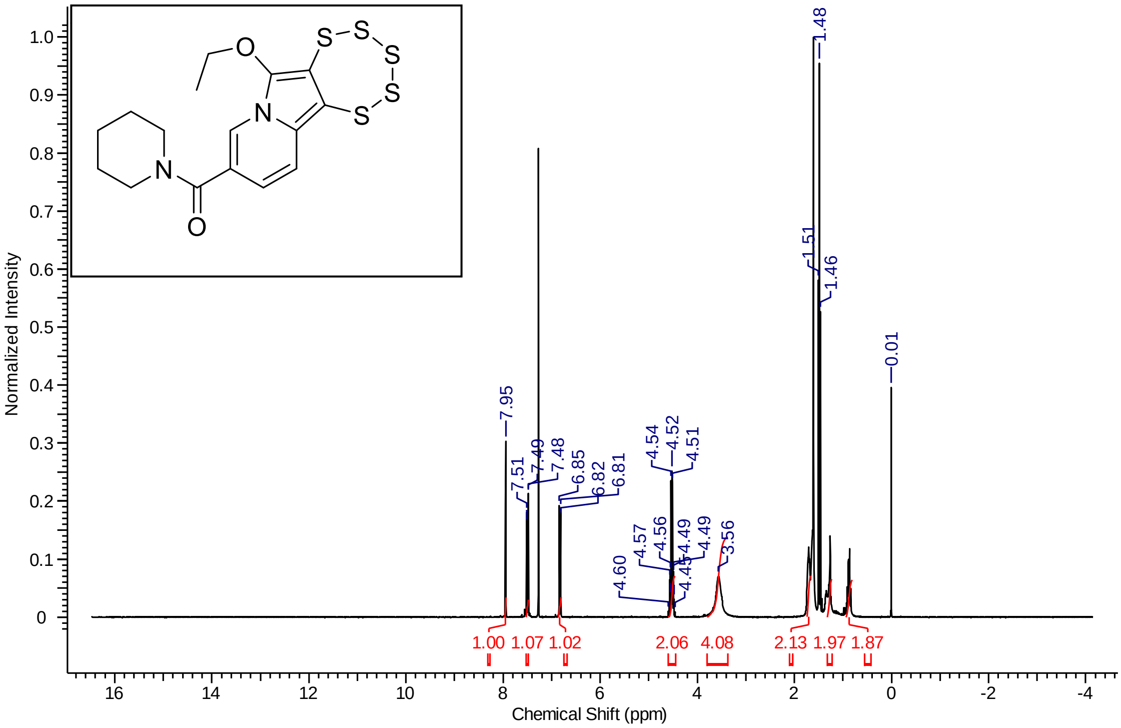

Figure S13: ^{1}H NMR spectrum of **2**.

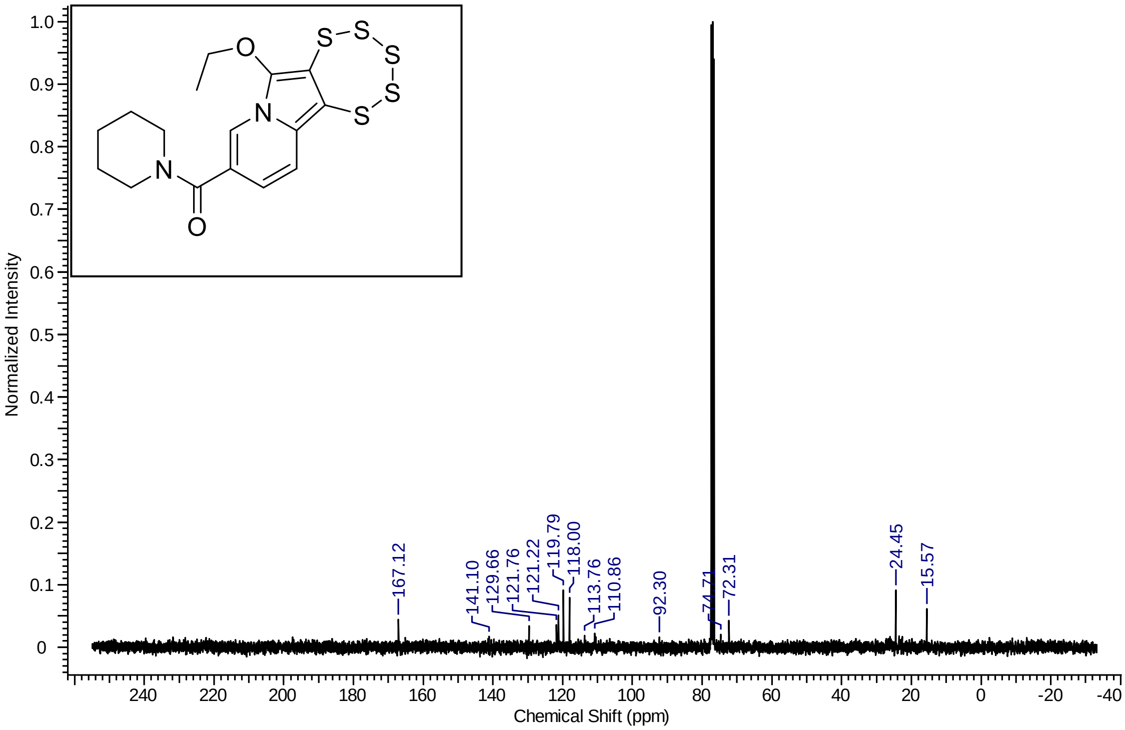

Figure S14: ^{13}C NMR spectrum of **2**.

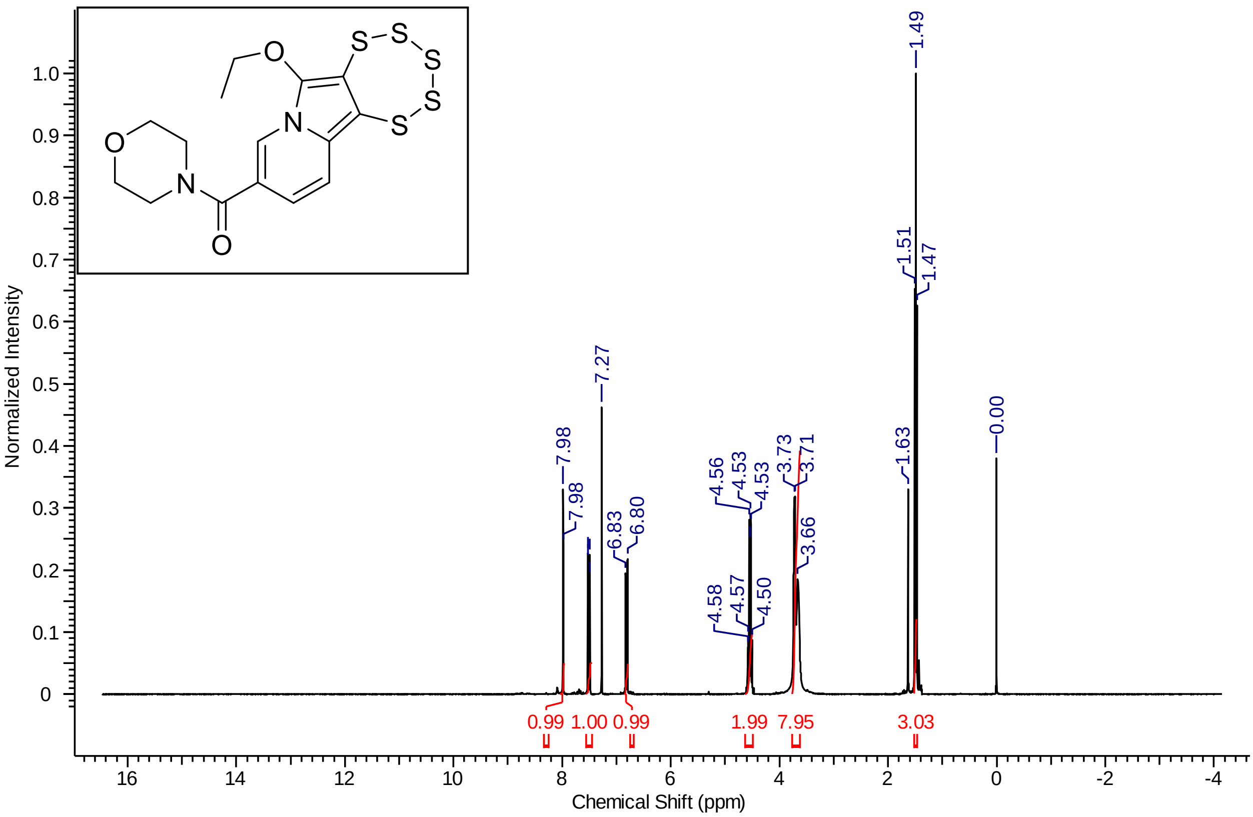

Figure S15: ^{1}H NMR spectrum of **3**.

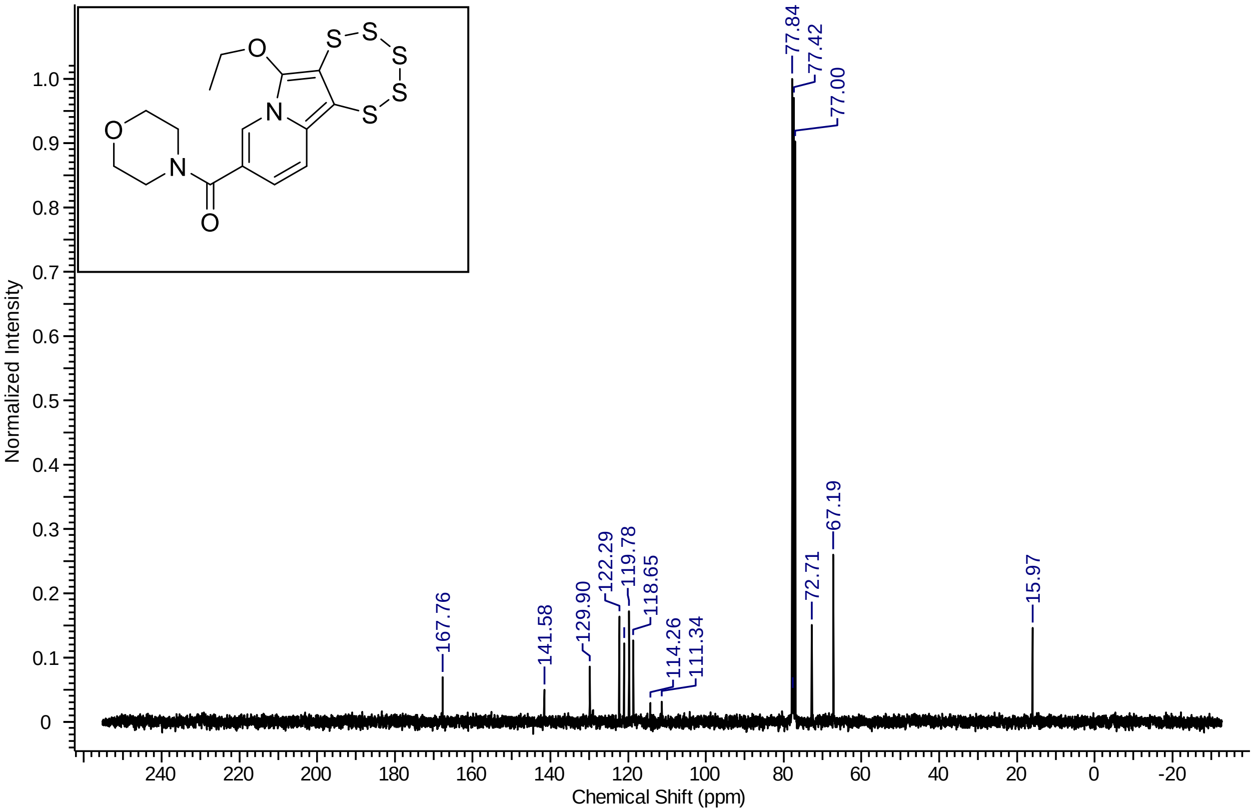

Figure S16: ^{13}C NMR spectrum of **3**.

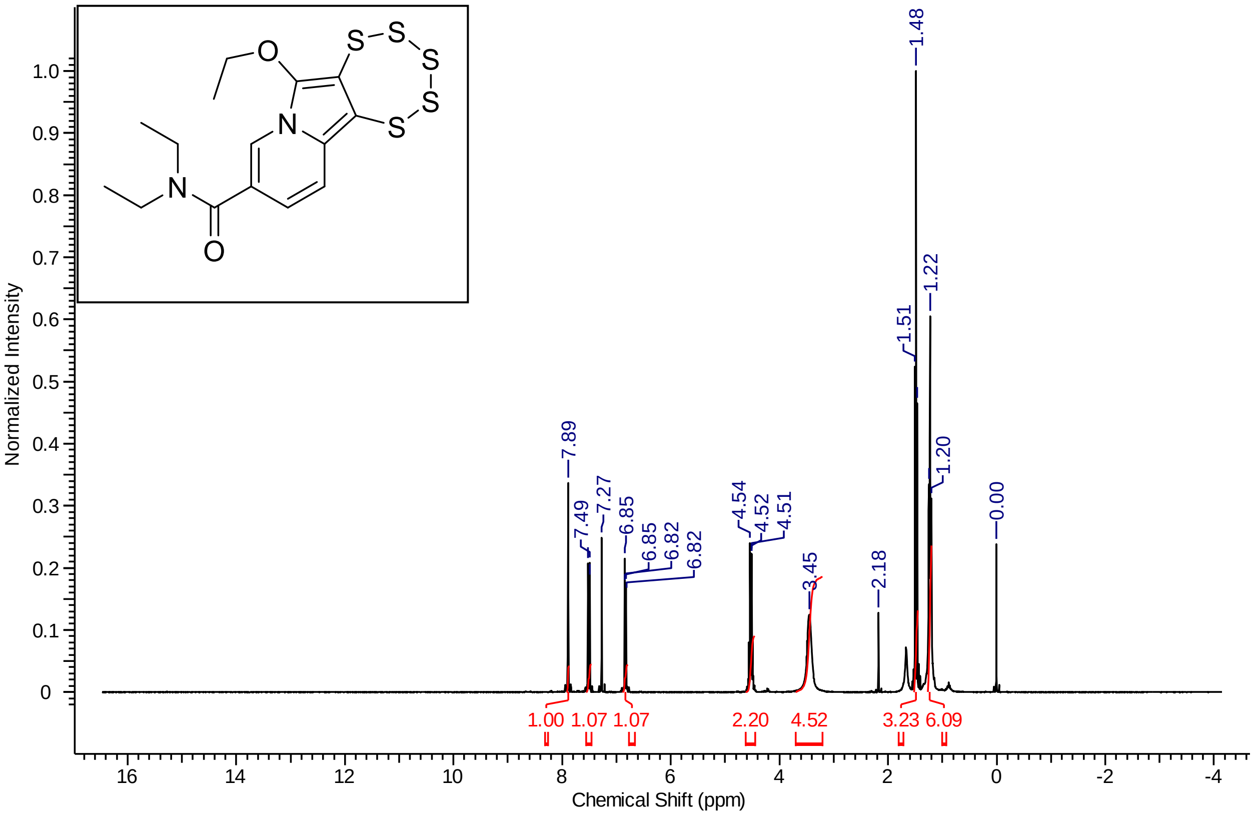

Figure S17: ^{1}H NMR spectrum of **4**.

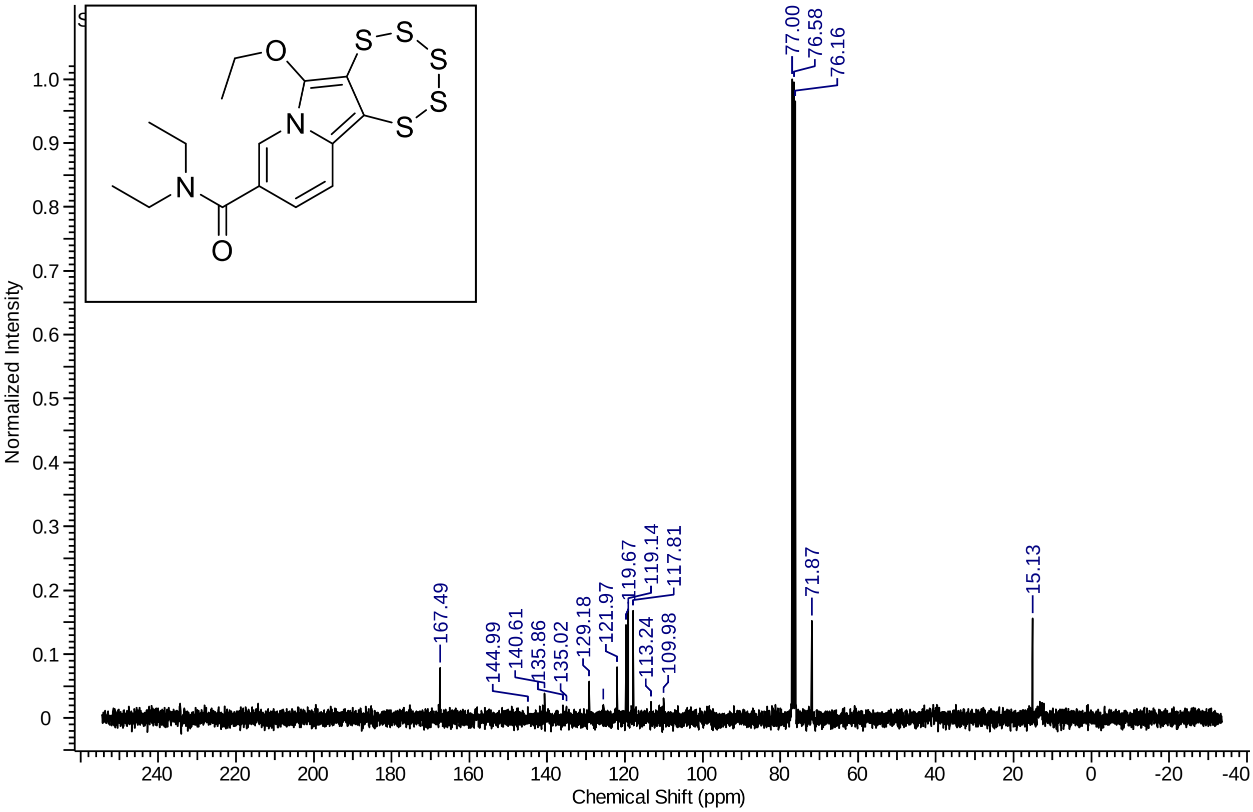

Figure S18: ^{13}C NMR spectrum of **4**.

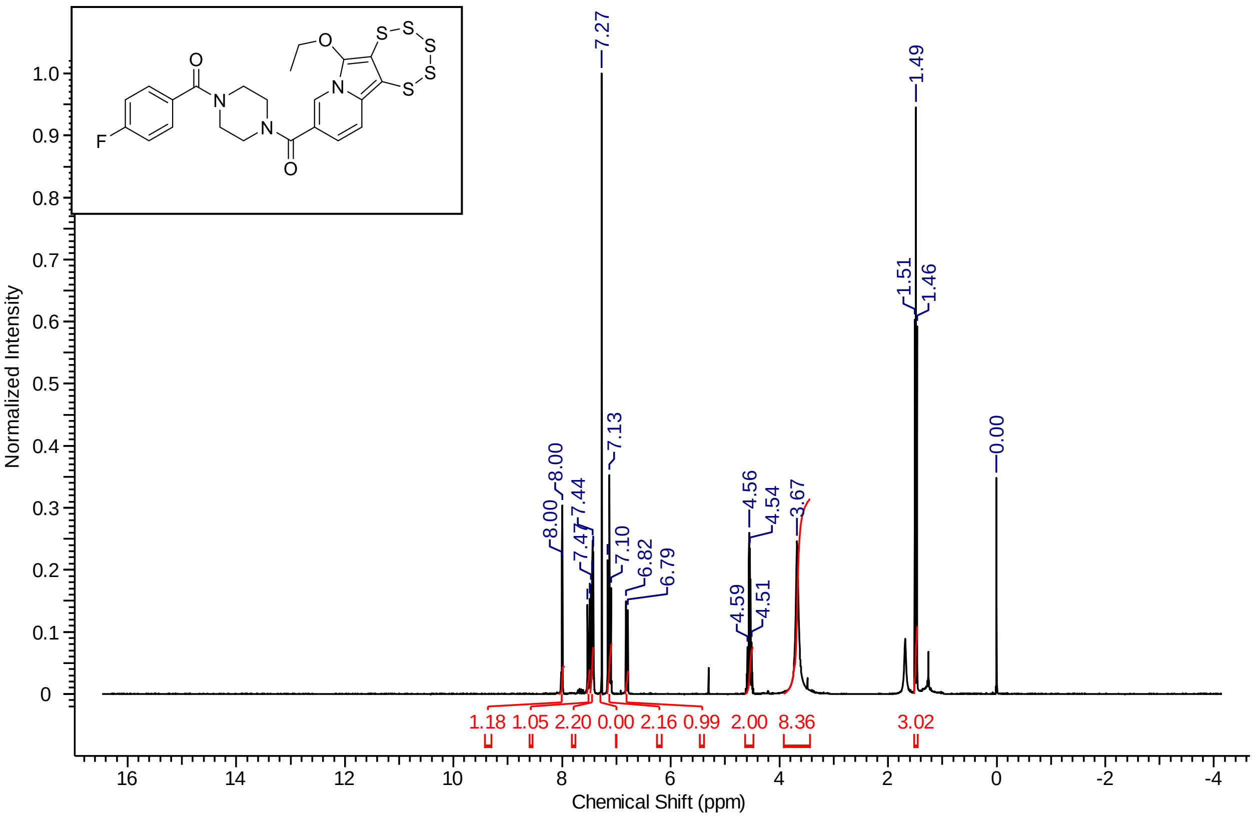

Figure S19: ^{1}H NMR spectrum of **5**.

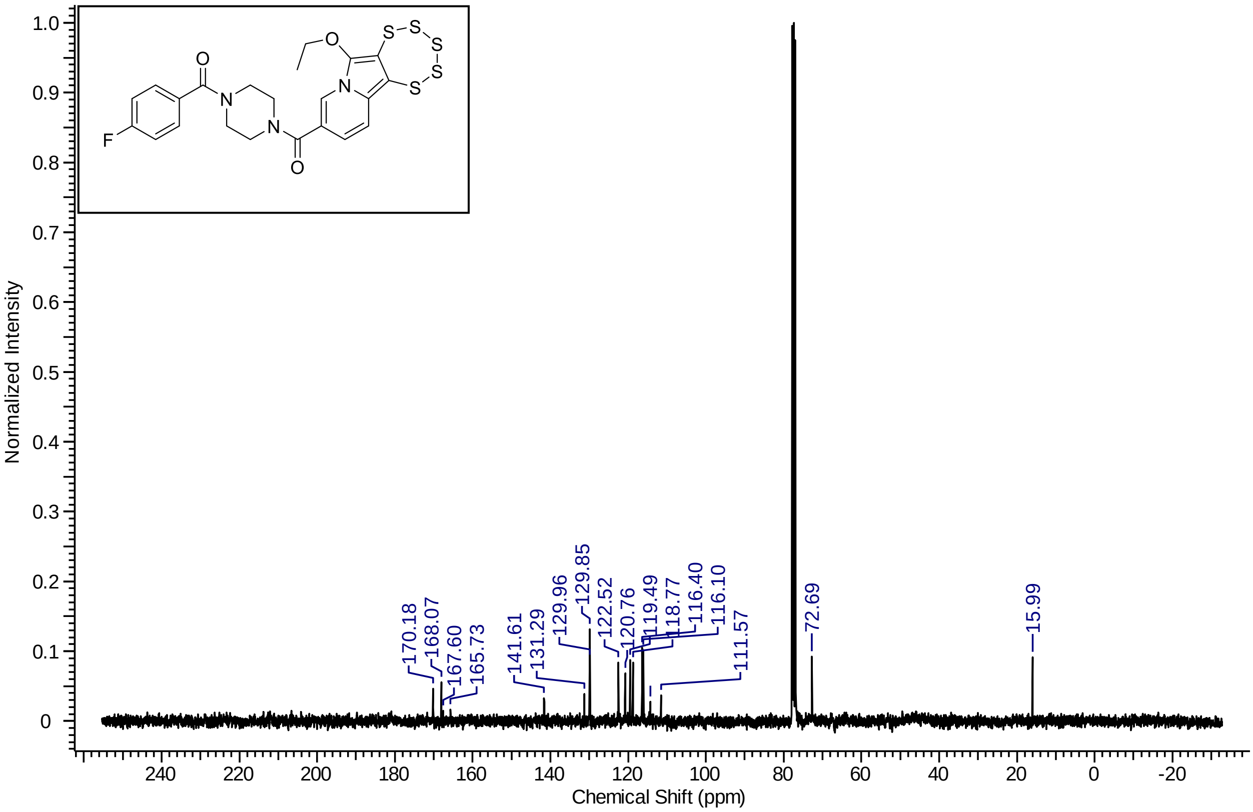

Figure S20: ^{13}C NMR spectrum of **5**.

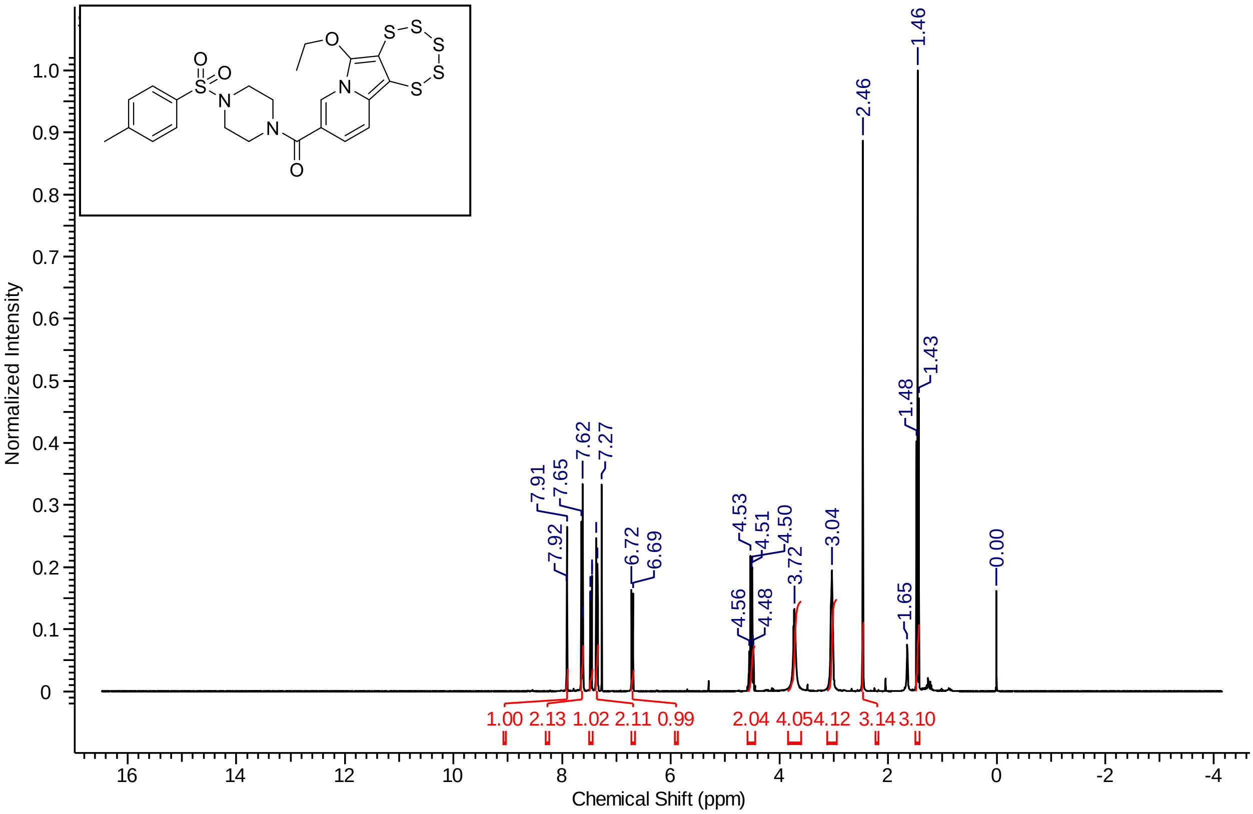

Figure S21: ^{1}H NMR spectrum of **6**.

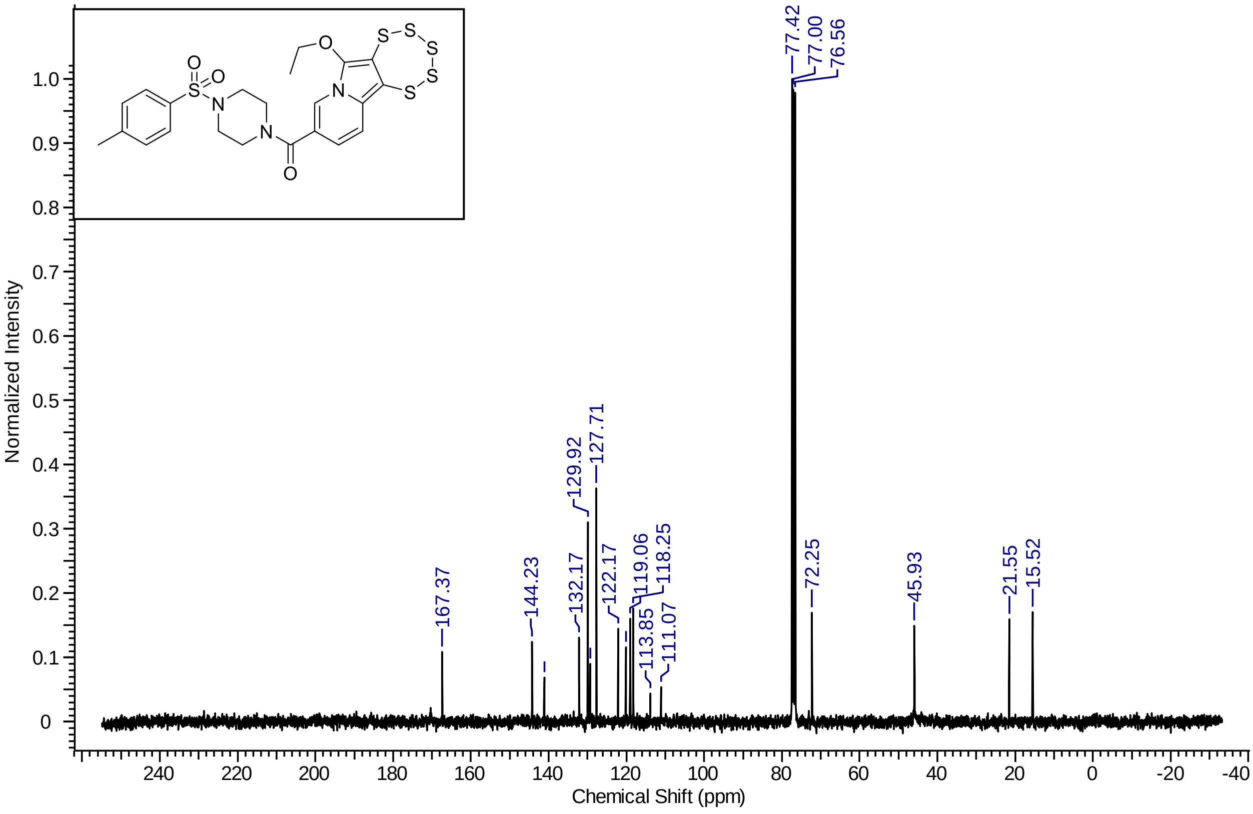

Figure S22: ^{13}C NMR spectrum of **6**.

APCI-MS spectra of 1-6

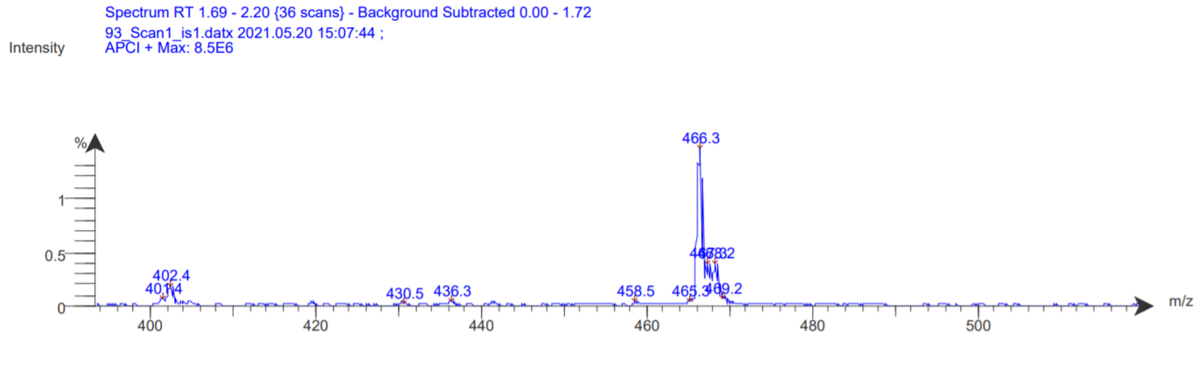

Figure S23: APCI-MS spectrum of **1**.

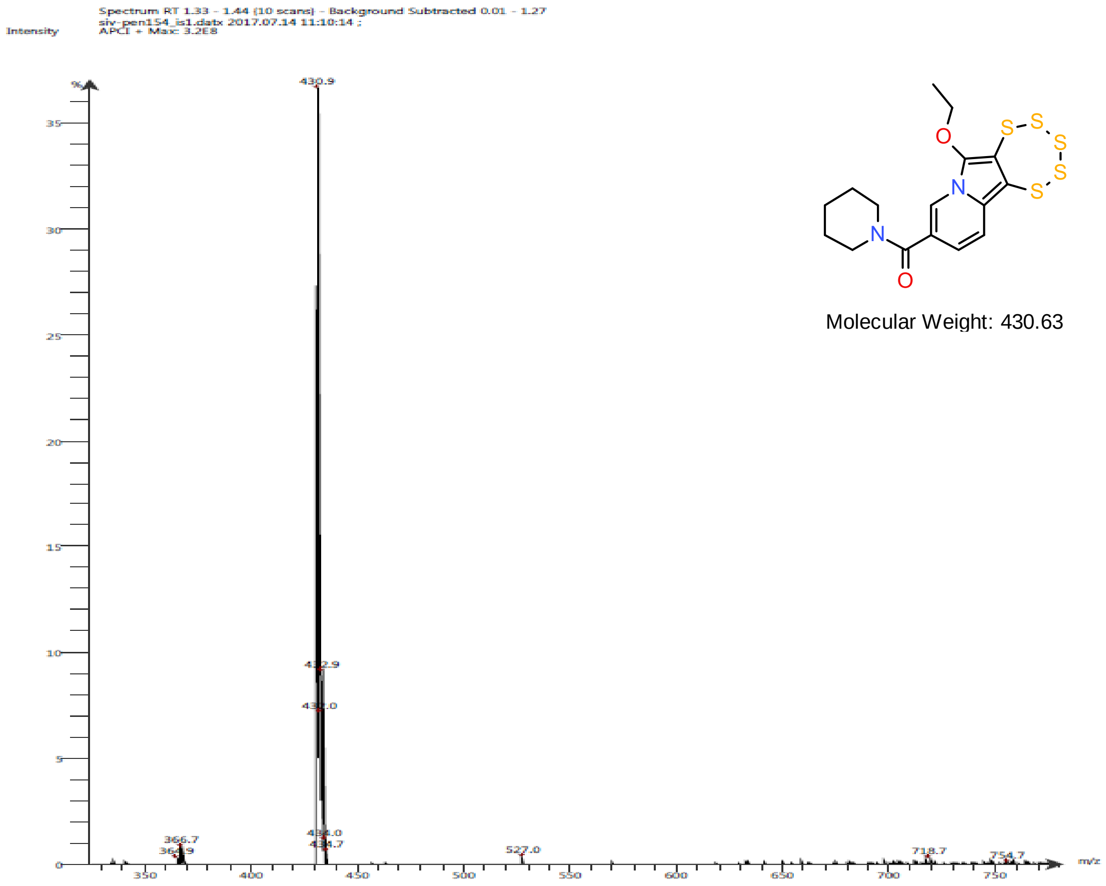

Figure S24: APCI-MS spectrum of **2**.

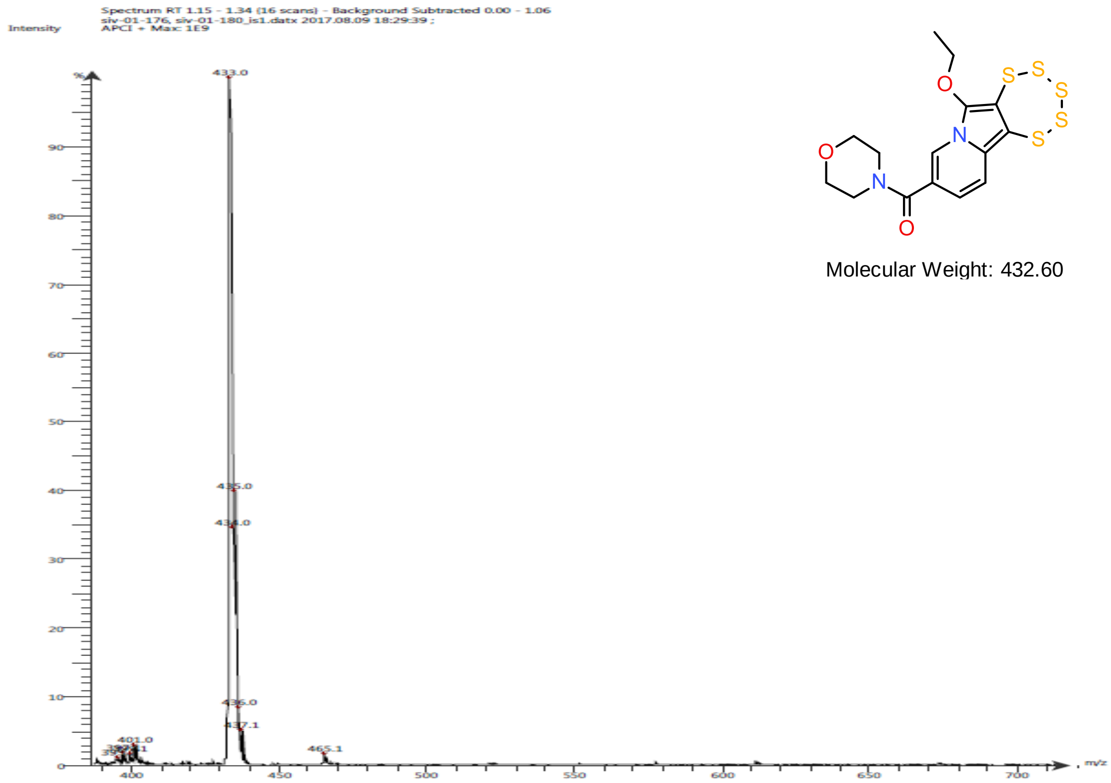

Figure S25: APCI-MS spectrum of **3**.

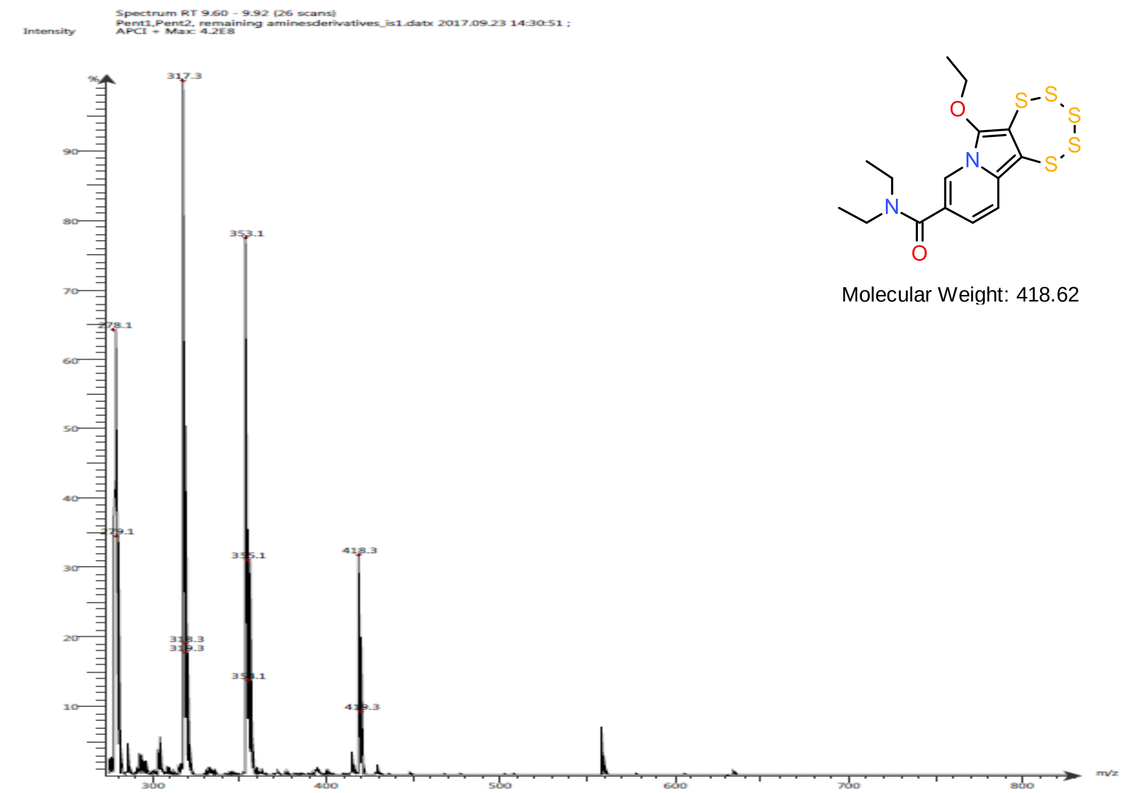

Figure S26: APCI-MS spectrum of **4**.

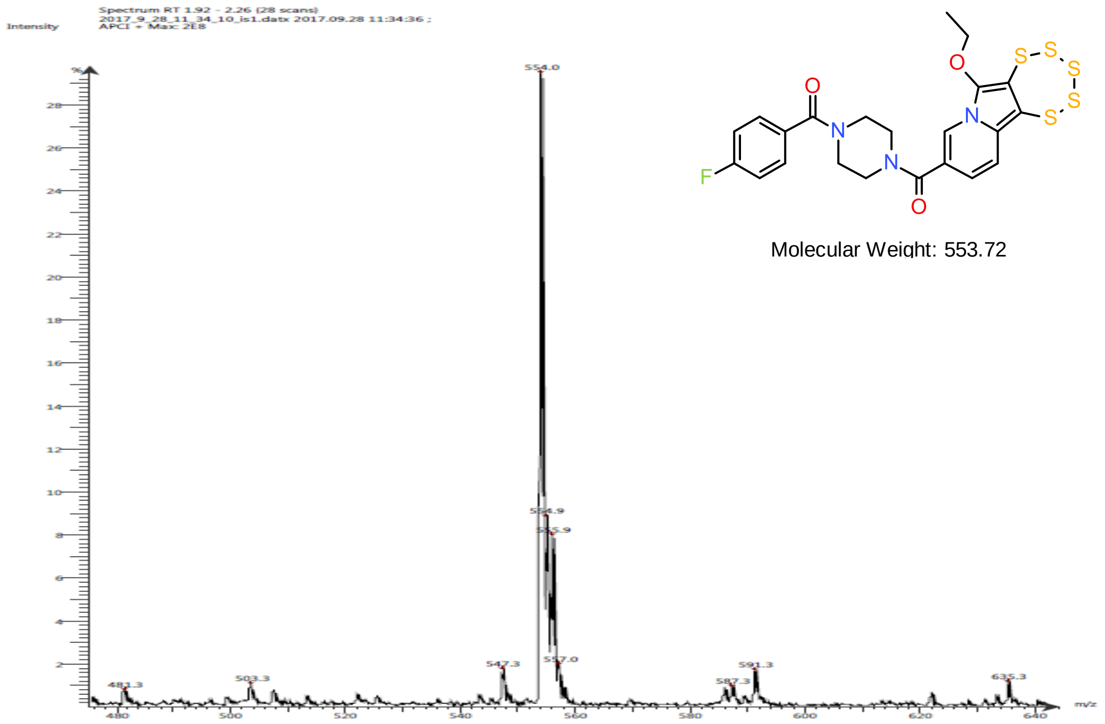

Figure S27: APCI-MS spectrum of **5**.

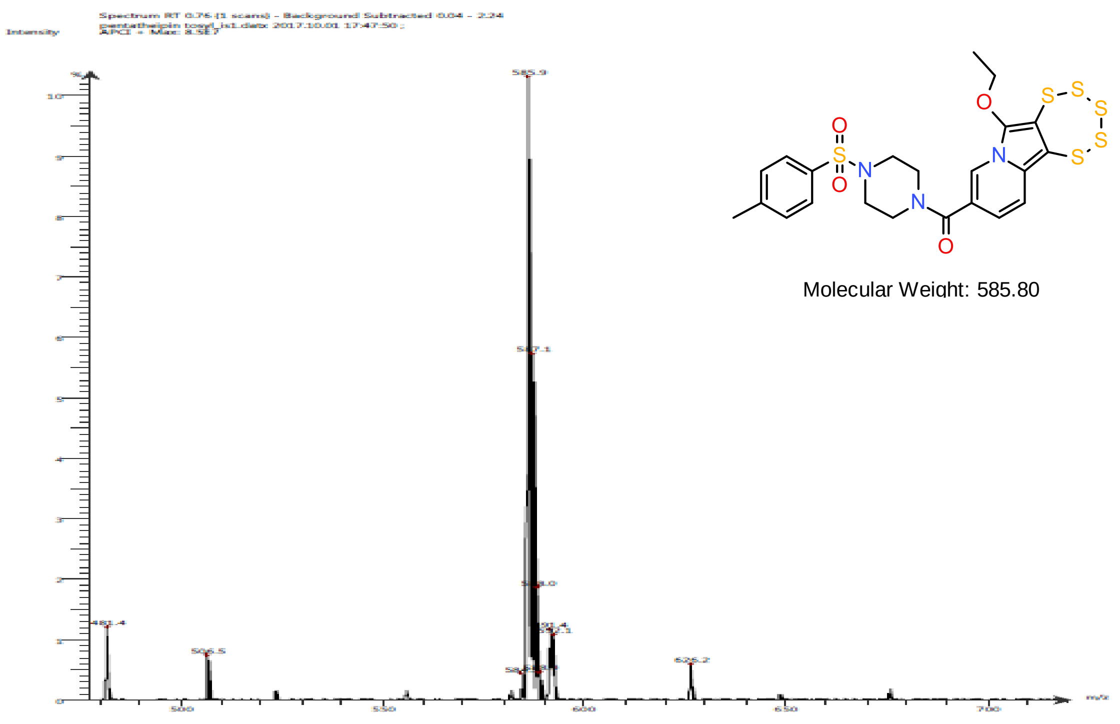

Figure S28: APCI-MS spectrum of **6**.

X-ray single-crystal diffraction analysis data:

Suitable single crystals of **2**, **3** and **4** were mounted on a thin glass fiber coated with paraffin oil. X-ray single-crystal structural data were collected using a STOE-IPDS II diffractometer equipped with a normal-focus 2.4 kW sealed-tube X-ray source with graphite-monochromated Mo K_α radiation (λ = 0.71073 Å) at room temperature or at low temperature (170 K). The program XArea was used for integration of diffraction profiles; numerical absorption corrections were carried out with the programs X-Shape and X-Red32; all from STOE©. The structures were solved by direct methods with SHELXT (G. Sheldrick, SHELXT - Integrated space-group and crystal-structure determination, Acta Cryst. A 71 (2015) 3-8.) and refined by full-matrix least-squares methods using SHELXL (G. Sheldrick, Crystal structure refinement with SHELXL, Acta Cryst. C 71 (2015) 3-8.). All calculations were carried out using the WinGX system, Ver 2018.3 (L. Farrugia, WinGX and ORTEP for Windows: an update, J. Appl. Crystallogr. 45 (2012) 849-854.) or an earlier version. All non-hydrogen atoms were refined anisotropically. The hydrogen atoms were refined isotropically on calculated positions using a riding model with their U_{iso} values constrained to 1.5 U_{eq} of their pivot atoms for methyl groups and 1.2 U_{eq} of their pivot atoms for all other groups unless stated otherwise. In the structure of compound **2** the morpholin ring is disordered over two oreintations with occupancies of 0.80 % and 20 %. This was modelled using SIMU, DELU and SADI constraints. The crystal was twinned and the reflexes of the major domain were extracted with XArea during integration in order to generate the hkl file which was used for the refinement. As a consequence the internal R-value is comparably high. General crystallographic, crystal and refinement data for the four structures are summarized in the table below. Crystallographic data were deposited with the Cambridge Crystallographic Data Centre, CCDC, 12 Union Road, Cambridge CB21EZ, UK. These data can be obtained free of charge on quoting the

depository numbers CCDC 2079764 (**2**) 2079765 (**3**) and 2079766 (**4**) by FAX (+44-1223-336-033), email (deposit@ccdc.cam.ac.uk) or their web interface (at http://www.ccdc.cam.ac.uk).

Table S1: Crystal and refinement data for the X-ray structural analyses of pentathiepins **2**, **3** and **4**.

Identifier	**2**	**3**	**4**
CCDC number	2079764	2079765	2079766
Chemical formula	$C_{16}H_{18}N_2O_2S_5$	$C_{15}H_{16}N_2O_3S_5$	$C_{15}H_{18}N_2O_2S_5$
M_r (g·mol^{-1})	430.62	432.60	418.61
Crystal system, space group	Orthorhombic, *Pbca*	Orthorhombic, *Pbca*	Triclinic, *P*-1
Temperature (K)	170	298	298
a, b, c (Å)	11.742 (2), 17.417 (3), 18.094 (4)	11.924 (2), 17.249 (3), 17.974 (4)	9.3490 (19), 10.261 (2), 11.274 (2)
α, β, γ (°)	90, 90, 90	90, 90, 90	70.63 (3), 81.12 (3), 66.08 (3)
V (Å^3)	3700.4 (12)	3696.8 (12)	932.4 (4)
Z	8	8	2
Absorption coefficient (mm^{-1})	0.64	0.65	0.63
F(000)	1792	1792	436
Crystal size (mm)	0.19 × 0.13 × 0.04	0.28 × 0.16 × 0.08	0.50 × 0.48 × 0.44
Theta range for data collection (°)	2.251 to 26.361	3.074 to 24.718	3.383 to 29.464
Limiting indices	-14<=h<=14, -19<=k<=21, -22<=l<=22	-13<=h<=14, -20<=k<=20, -20<=l<=21	-12<=h<=12, -13<=k<=14, -15<=l<=15
Absorption correction	none	Numerical, face indexed	Numerical, face indexed
T_{min}, T_{max}		0.863, 0.944	0.808, 0.878
Completeness to theta	100.0 %	99.8 %	99.5 %
Refinement method	Full-matrix least-squares on F^2	Full-matrix least-squares on F^2	Full-matrix least-squares on F^2
No. of measured, independent , observed [I > 2σ(I)] reflections	30042, 3784, 2095	25566, 3148, 1674	10431, 5100, 3388
R_{int}	0.167	0.114	0.033
(sin q/l)$_{max}$ (Å^{-1})	0.625	0.588	0.692
Goodness-of-fit on F^2	0.898	0.882	1.012
Final R indices [I>2sigma(I)]	R1 = 0.0475, wR2 = 0.0860	R1 = 0.0408, wR2 = 0.0848	R1 = 0.0423, wR2 = 0.1016
R indices (all data)	R1 = 0.1133, wR2 = 0.1060	R1 = 0.0955, wR2 = 0.0994	R1 = 0.0733, wR2 = 0.1137
Data / restraints / parameters	3784 / 0 / 227	3148 / 115 / 248	5100 / 0 / 220
Dñ$_{max}$, Dñ$_{min}$ (e Å^{-3})	0.36, -0.34	0.28, -0.24	0.43, -0.32

Biology

Original Images: Western Blots and total protein images for normalization

Table S2. Annotation table for the original Western blot images (Figures S23-S28).

**Cell Line**	**HAP-1**	**HAP-1.KO.GPx1**	**A2780**	**Siso**
Annotation	A	B	C	D
Solvent DMF (neg. con)	1	1	1	1
Pentathiepin **1** (IC_{90})	2	2	2	2
Pentathiepin **2** (IC_{90})	3	3	3	3
Pentathiepin **3** (IC_{90})	4	4	4	4
Pentathiepin **4** (IC_{90})	5	5	5	5
Pentathiepin **5** (IC_{90})	6	6	6	6
Pentathiepin **6** (IC_{90})	7	7	7	7
Doxorubicin 0.5 µM (pos. con)	8	8	8	8

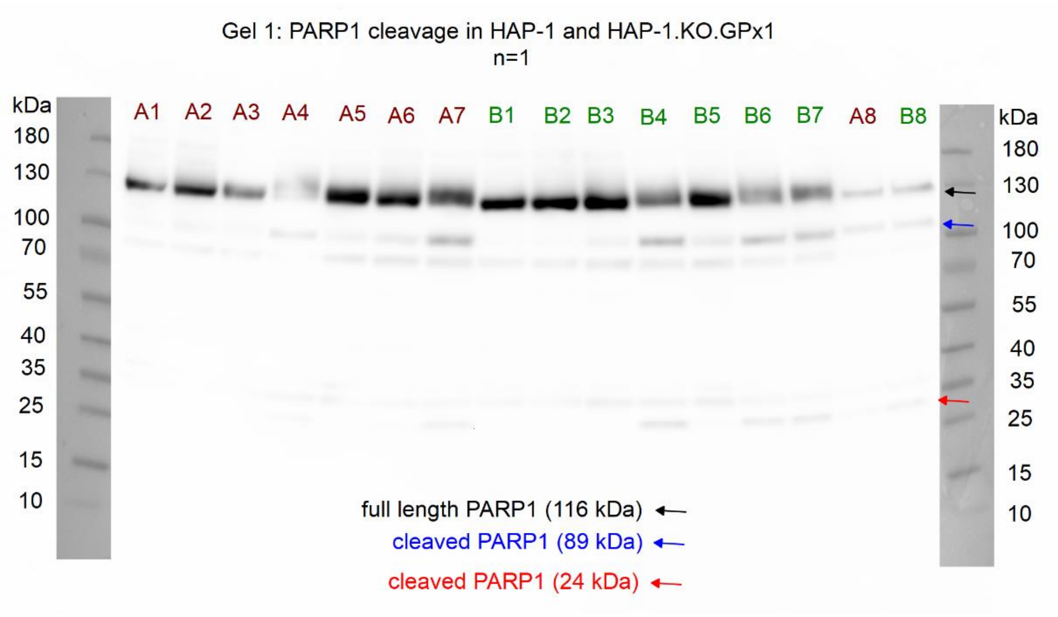

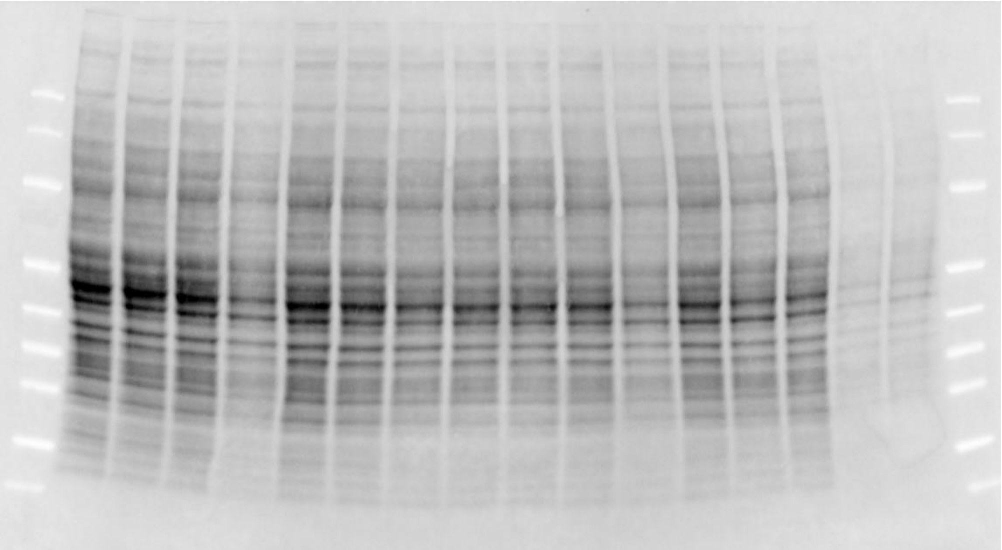

Figure S29: Original images. Top: Detection of chemiluminescent signal after incubation with anti-PARP1 primary (rb) and goat anti-rabbit-HRP secondary antibody providing data for Figure 17. Bottom: Image of stain-free blot for total protein normalisation.

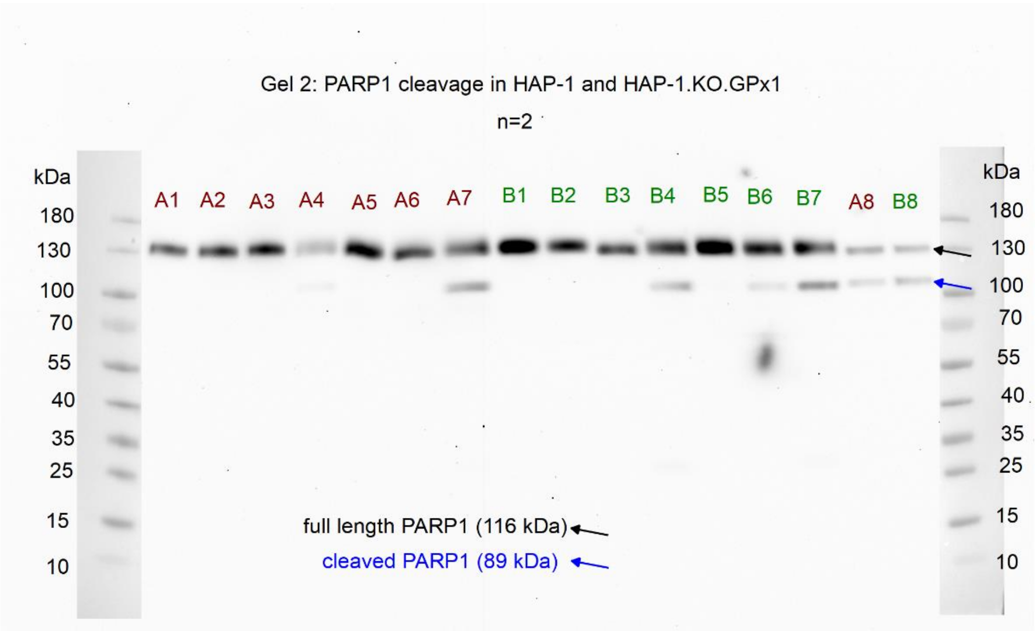

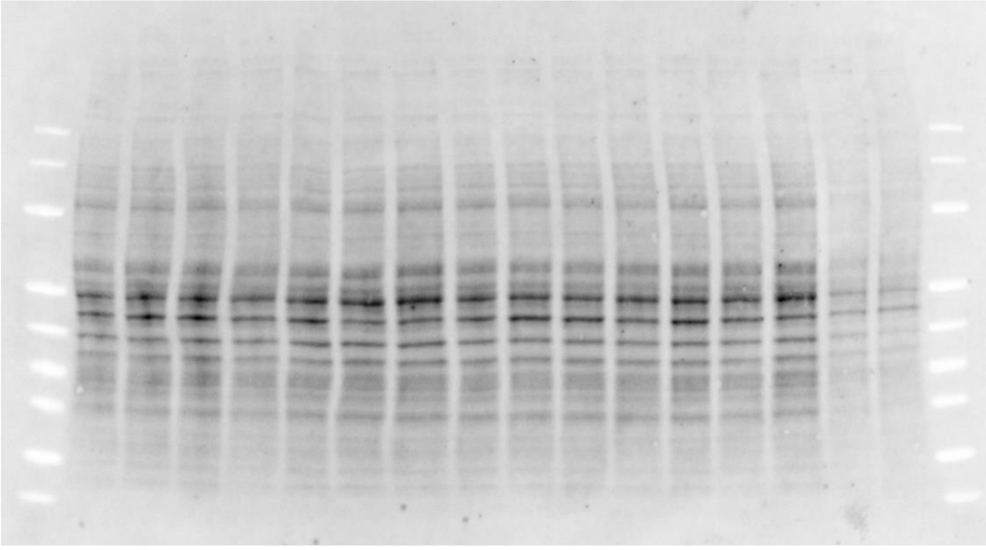

Figure S30: Original images. Top: Detection of chemiluminescent signal after incubation with anti-PARP1 primary (rb) and goat anti-rabbit-HRP secondary antibody providing data for Figure 17. Bottom: Image of stain-free blot for total protein normalisation.

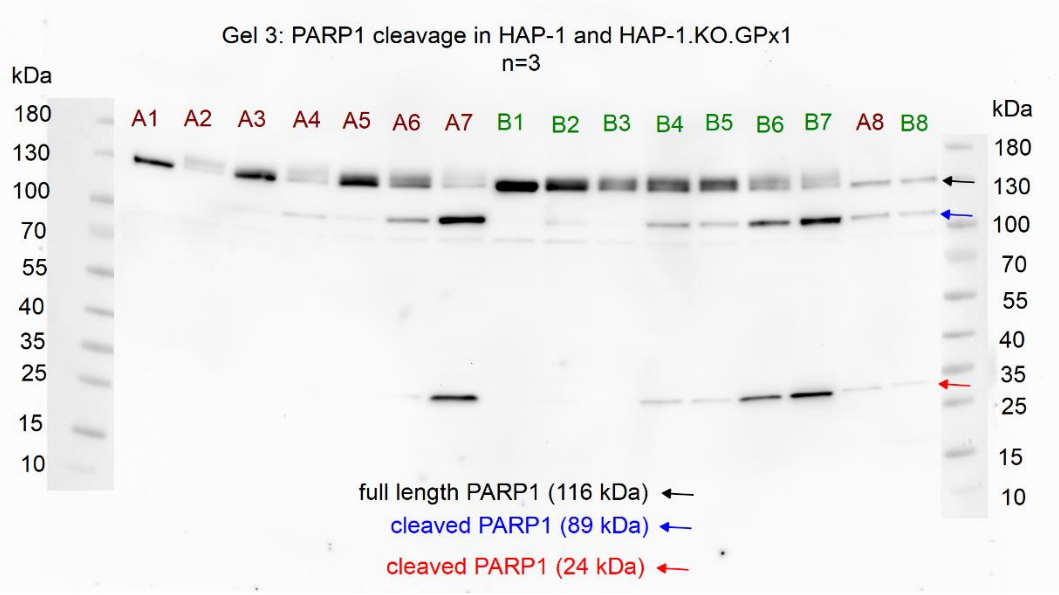

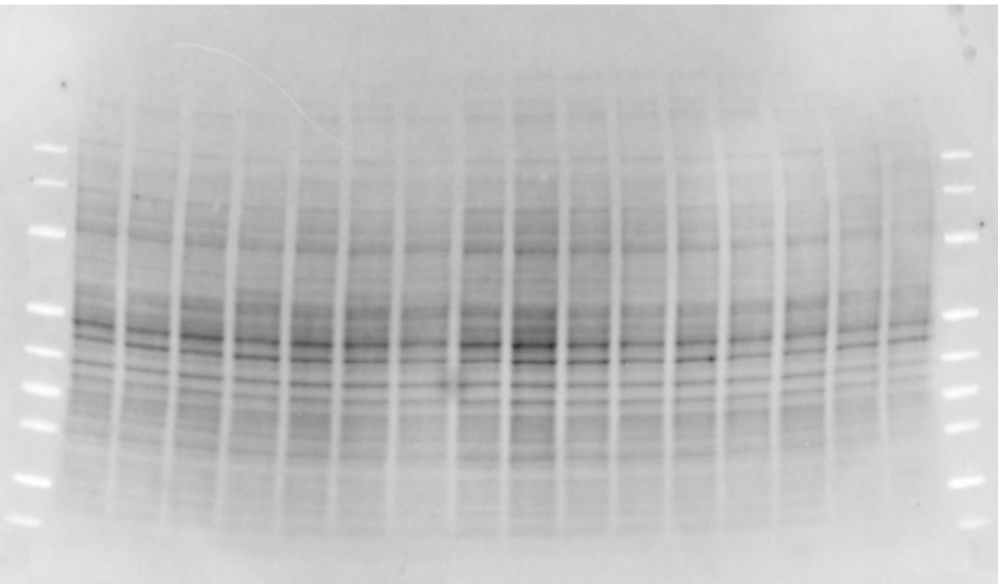

Figure S31: Original images. Top: Detection of chemiluminescent signal after incubation with anti-PARP1 primary (rb) and goat anti-rabbit-HRP secondary antibody providing data for Figure 17. Bottom: Image of stain-free blot for total protein normalisation.

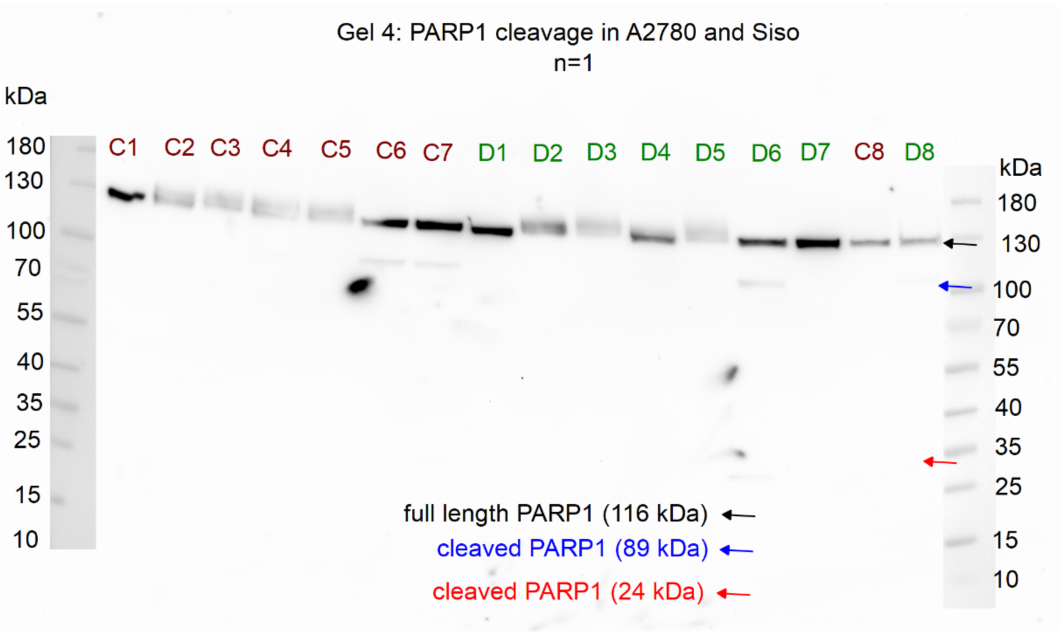

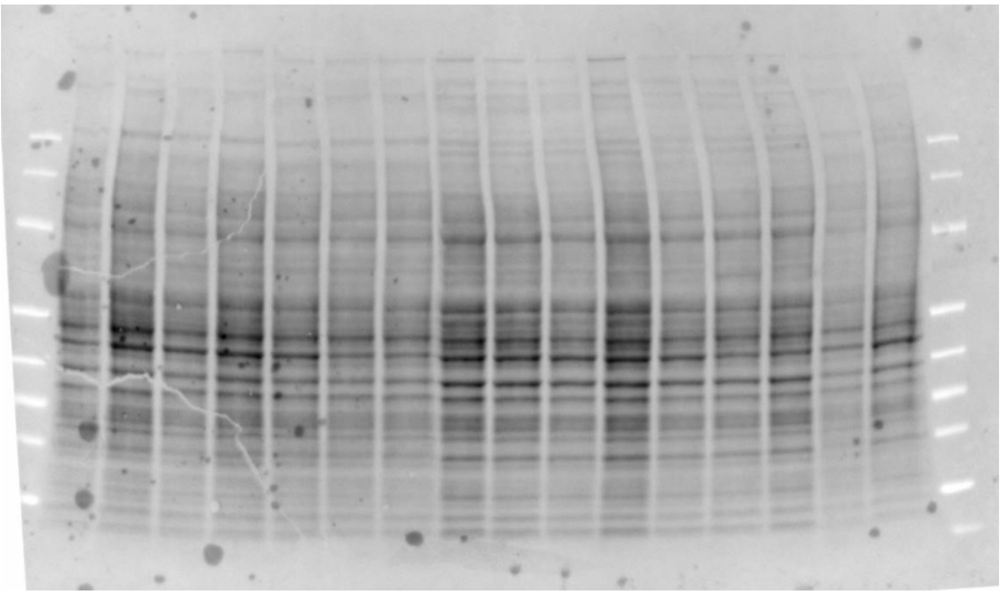

Figure S32: Original images. Top: Detection of chemiluminescent signal after incubation with anti-PARP1 primary (rb) and goat anti-rabbit-HRP secondary antibody providing data for Figure 17. Bottom: Image of stain-free blot for total protein normalisation.

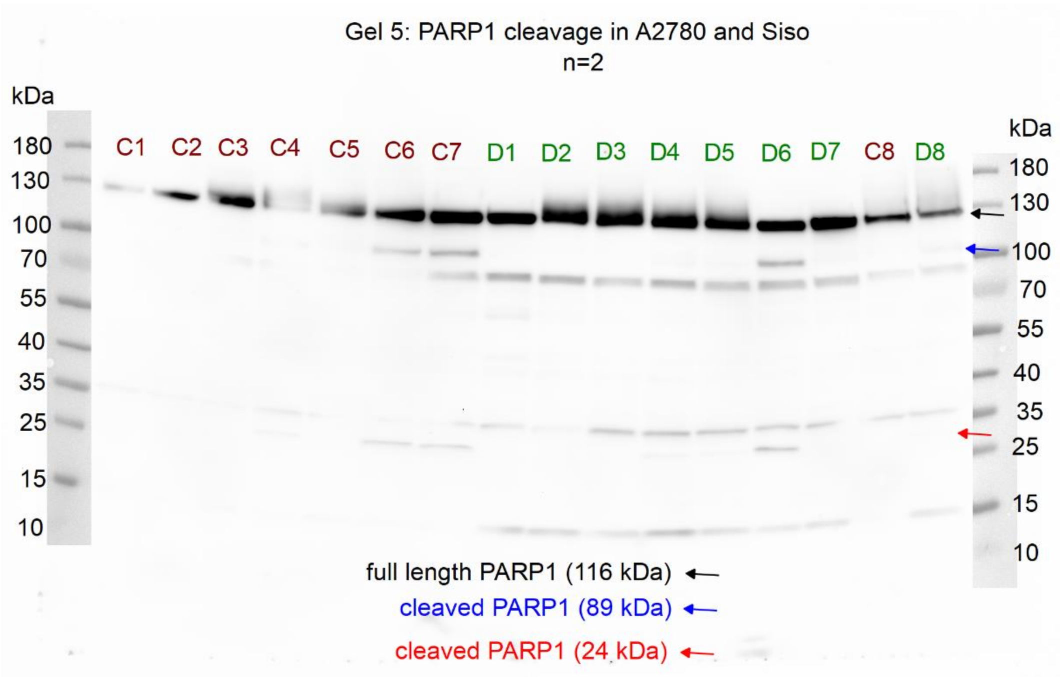

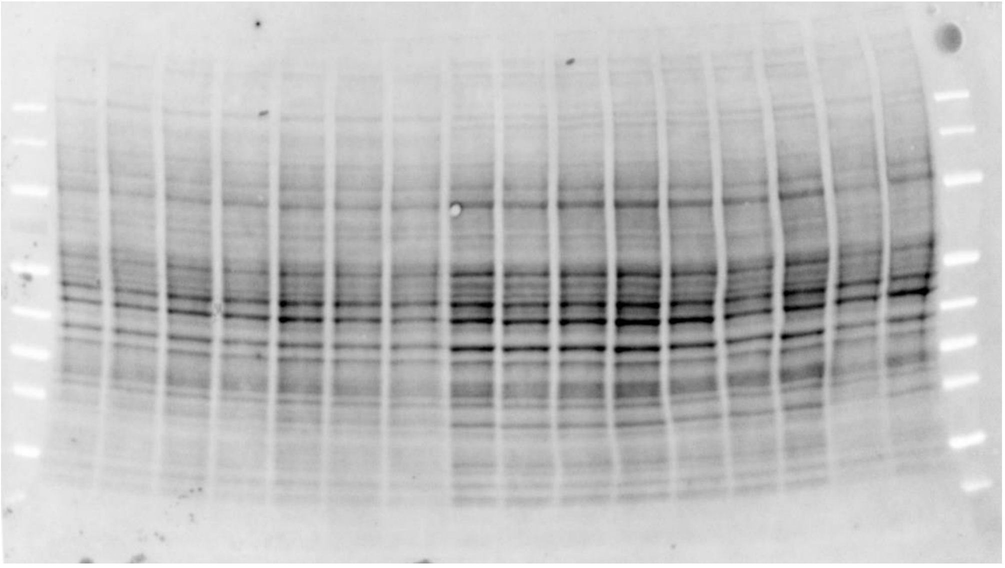

Figure S33: Original images. Top: Detection of chemiluminescent signal after incubation with anti-PARP1 primary (rb) and goat anti-rabbit-HRP secondary antibody providing data for Figure 17. Bottom: Image of stain-free blot for total protein normalisation.

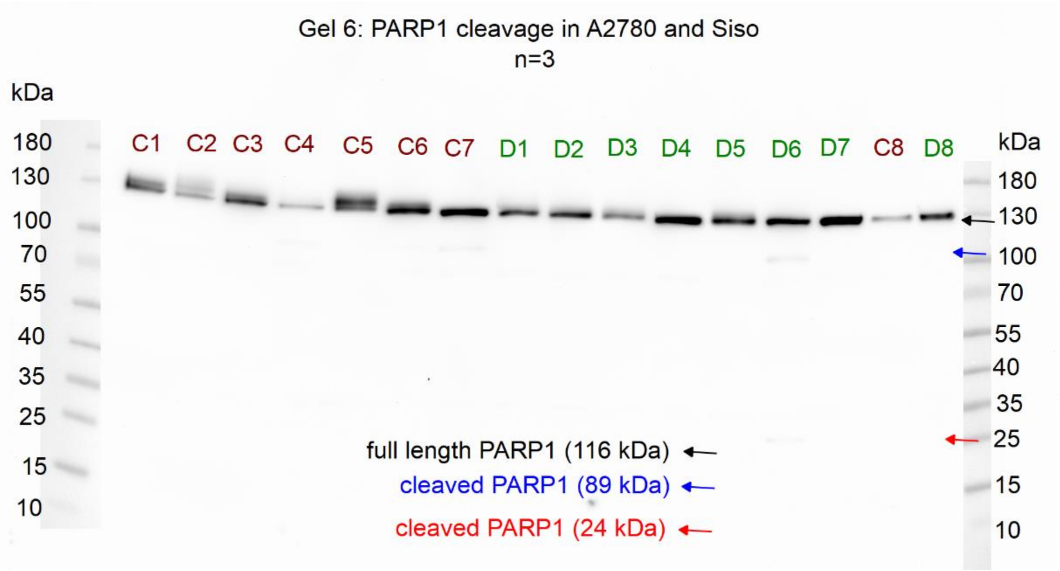

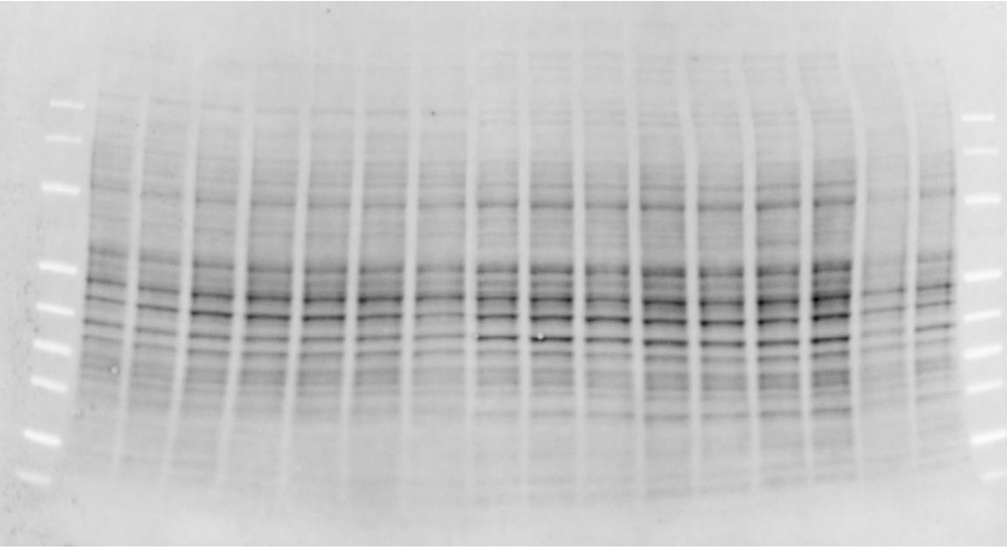

Figure S34: Original images. Top: Detection of chemiluminescent signal after incubation with anti-PARP1 primary (rb) and goat anti-rabbit-HRP secondary antibody providing data for Figure 17. Bottom: Image of stain-free blot for total protein normalisation.

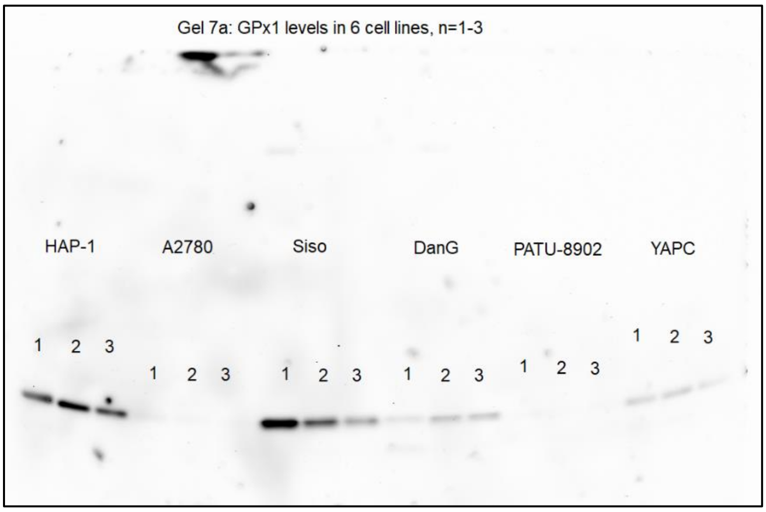

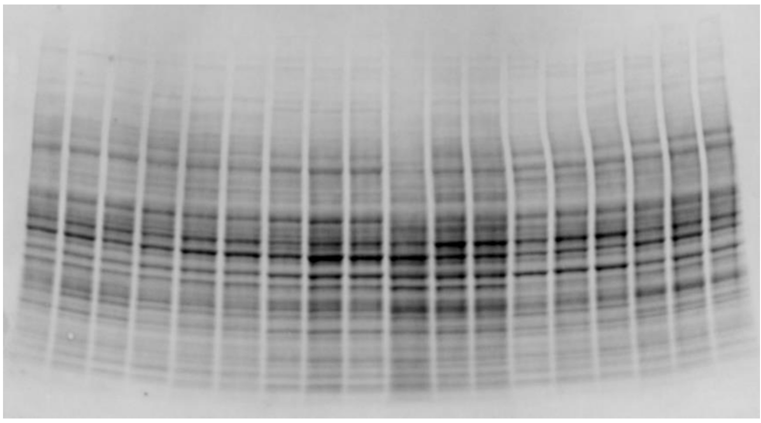

Figure S35: Original images. Top: Detection of chemiluminescent signal after incubation with anti-GPx1 primary (rb) and goat anti-rabbit-HRP secondary antibody providing data for Figure 7. Bottom: Image of stain-free blot for total protein normalisation.

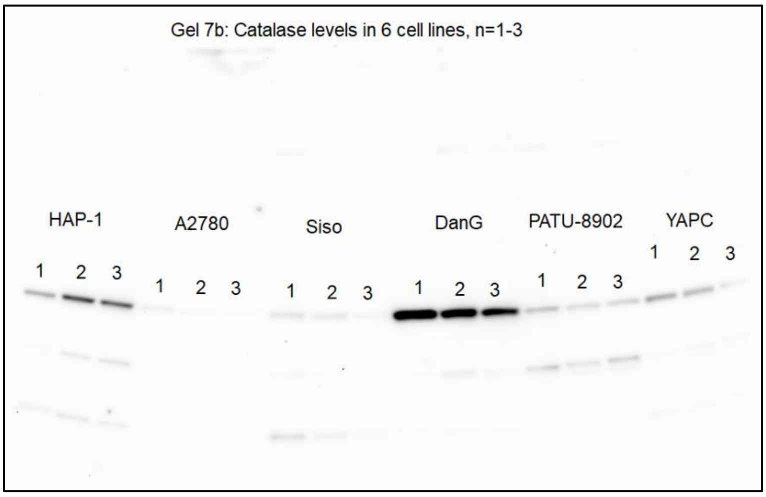

Figure S36: Original images. Top: Detection of chemiluminescent signal after incubation with anti-Catalase primary (rb) and goat anti-rabbit-HRP secondary antibody providing data for Figure 7. Image of stain-free blot for total protein normalisation is displayed in figure S29.

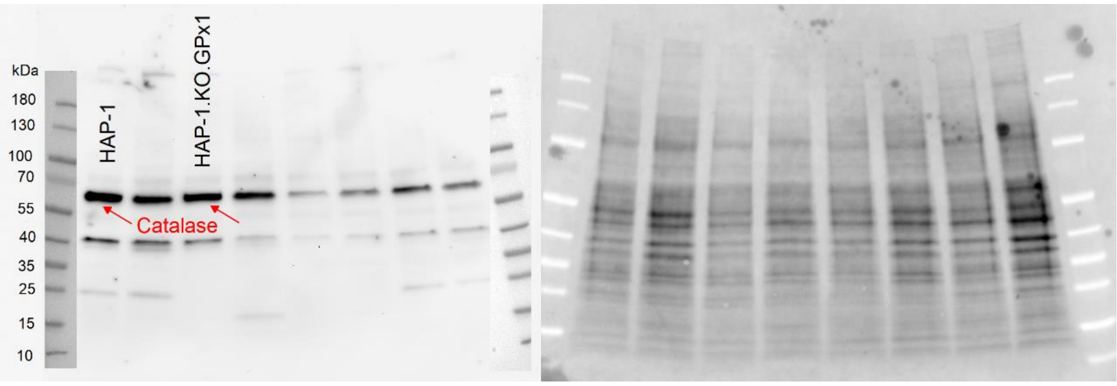

Figure S37: Original images. Left: Detection of chemiluminescent signal after incubation with anti-Catalase primary (rb) and goat anti-rabbit-HRP secondary antibody providing data for Figure 7. n=1. Right: Image of stain-free blot for total protein normalisation.

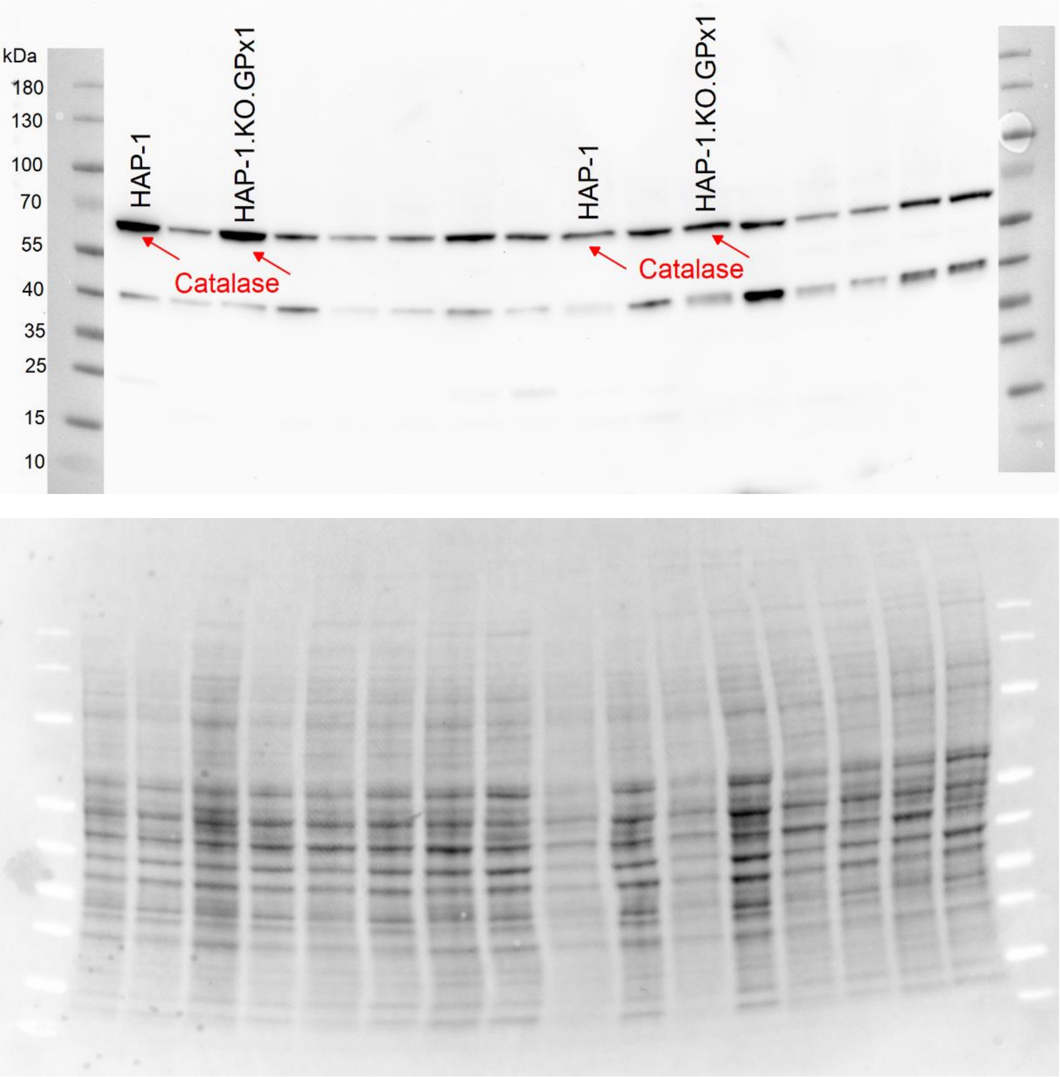

Figure S38: Original images. Top: Detection of chemiluminescent signal after incubation with anti-Catalase primary (rb) and goat anti-rabbit-HRP secondary antibody providing data for Figure 7. n=2+3. Bottom: Image of stain-free blot for total protein normalisation.

Original Images: DNA gel electrophoresis

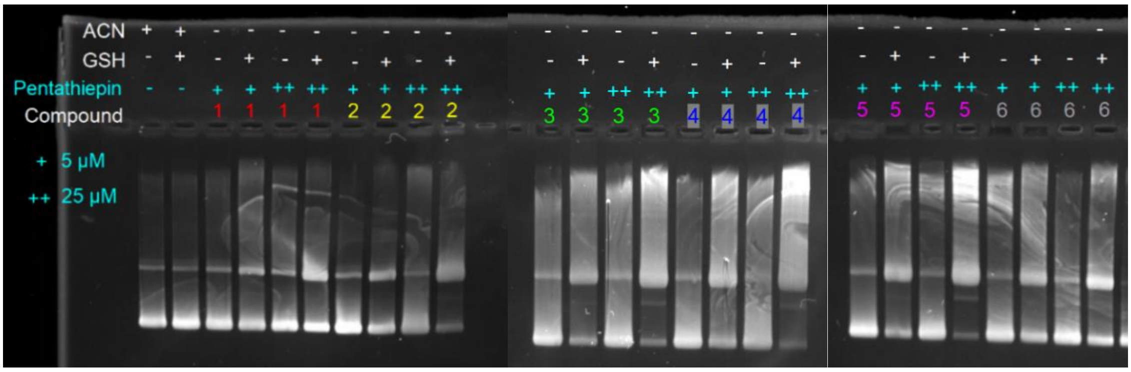

Figure S39: Original gel images providing data for Figure 11a, b, c. Samples from n=1 were run on three gels.

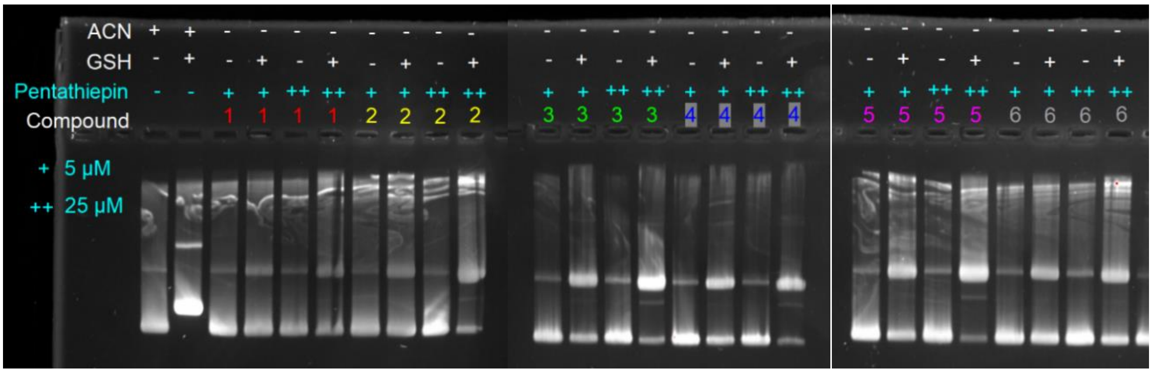

Figure S40: Original gel images providing data for Figure 11a, b, c. Samples from n=2 were run on three gels.

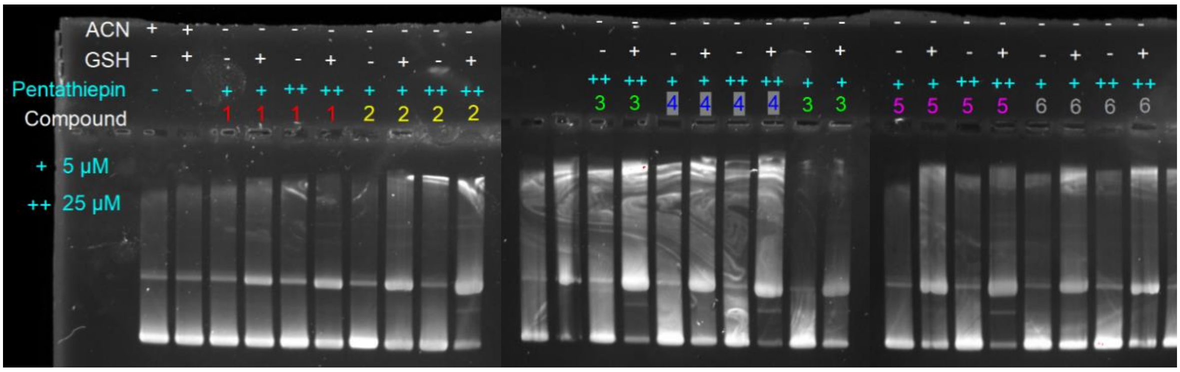

Figure S41: Original gel images providing data for Figure 11a, b, c. Samples from n=3 were run on three gels.

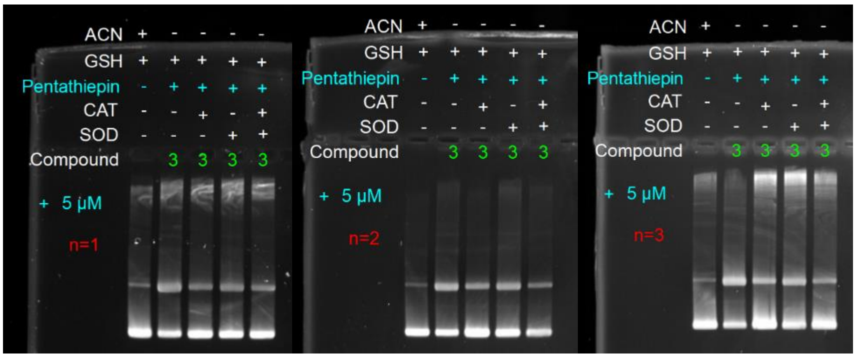

Figure S42: Original gel images providing data for Figure 11d. Samples from each replicate were run on a separate gel.

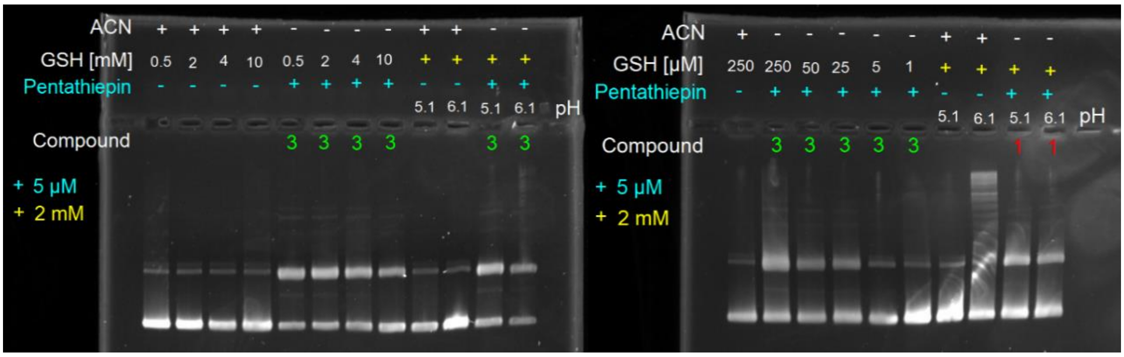

Figure S43: Original gel images providing data for Figure 11e, f. Samples from n=1 were run on two gels.

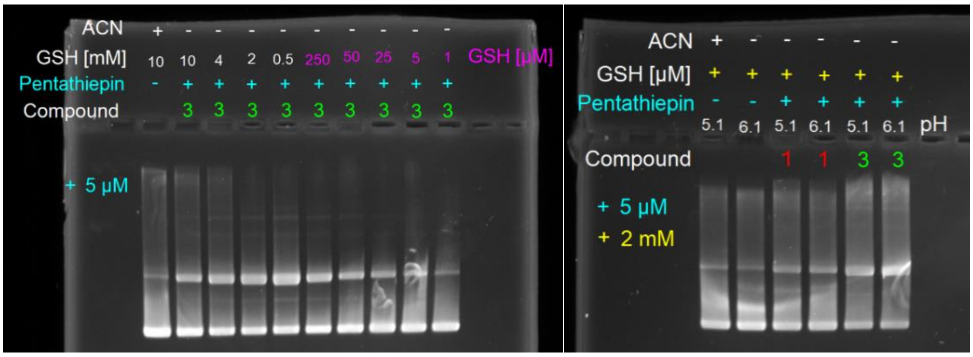

Figure S44: Original gel images providing data for Figure 11e, f. Samples from n=2 were run on two gels.

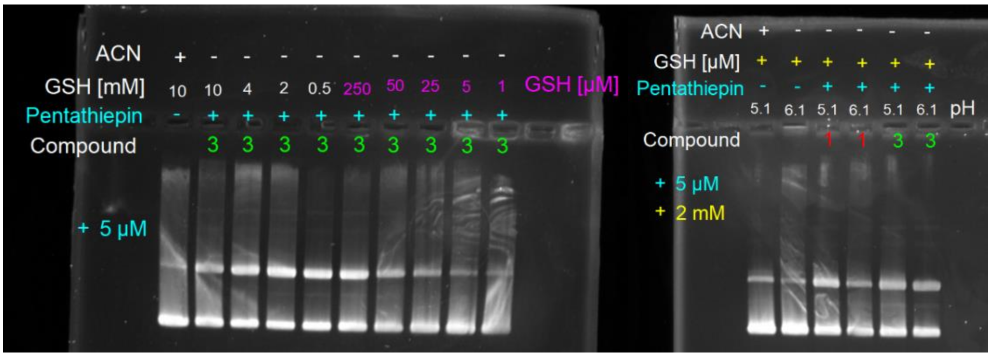

Figure S45: Original gel images providing data for Figure 11e, f. Samples from n=3 were run on two gels.

IC50 values of the pentathiepins in this study in the various cell lines tested

Table S3: IC_{50} and GI_{50} values in µM of the pentathiepins **1-6** determined with the MTT and crystal violet assay, respectively, including both normoxic (-N) and hypoxic (-H) incubation conditions. Listed are means and standard deviations (SD) from n≥3 independent experiments as well as means per compound (bottom line).

	**1**			**2**			**3**			**4**			**5**			**6**		
Assay<br>Cell line	MTT-N<br>mean<br>± SD	CV<br>mean<br>± SD	MTT-H<br>mean<br>± SD	MTT-N<br>mean<br>± SD	CV<br>mean<br>± SD	MTT-H<br>mean<br>± SD	MTT-N<br>mean<br>± SD	CV<br>mean<br>± SD	MTT-H<br>mean<br>± SD	MTT-N<br>mean<br>± SD	CV<br>mean<br>± SD	MTT-H<br>mean<br>± SD	MTT-N<br>mean<br>± SD	CV<br>mean<br>± SD	MTT-H<br>mean<br>± SD	MTT-N<br>mean<br>± SD	CV<br>mean<br>± SD	MTT-H<br>mean<br>± SD
HAP-1	0.398<br>0.09	0.339<br>0.04	0.384<br>0.10	0.600<br>0.15	0.161<br>0.03	0.847<br>0.28	0.263<br>0.06	0.064<br>0.01	0.657<br>0.21	0.263<br>0.05	0.084<br>0.01	0.832<br>0.09	0.270<br>0.03	0.120<br>0.05	0.661<br>0.08	0.722<br>0.29	0.507<br>0.12	1.325<br>0.29
HAP-1.<br>KO.GPx1	0.358<br>0.03	0.313<br>0.04	0.428<br>0.11	0.644<br>0.08	0.173<br>0.03	1.187<br>0.57	0.368<br>0.04	0.068<br>0.01	0.911<br>0.44	0.322<br>0.02	0.116<br>0.03	1.185<br>0.43	0.413<br>0.05	0.144<br>0.03	0.912<br>0.30	0.778<br>0.23	0.524<br>0.08	1.836<br>0.62
A2780	0.692<br>0.33	0.236<br>0.06	0.443<br>0.14	0.430<br>0.13	0.164<br>0.03	0.804<br>0.28	0.135<br>0.03	0.055<br>0.02	0.616<br>0.08	0.213<br>0.08	0.106<br>0.02	0.799<br>0.07	0.259<br>0.06	0.092<br>0.03	0.588<br>0.10	0.891<br>0.02	0.538<br>0.14	2.109<br>0.08
A2780cis	0.410<br>0.09	0.228<br>0.03	0.411<br>0.10	0.652<br>0.02	0.234<br>0.02	0.637<br>0.15	0.206<br>0.04	0.088<br>0.04	0.316<br>0.13	0.399<br>0.06	0.173<br>0.02	0.544<br>0.17	0.308<br>0.05	0.142<br>0.03	0.449<br>0.06	1.507<br>0.45	0.512<br>0.08	1.719<br>0.48
Siso	0.414<br>0.05	0.256<br>0.05	0.602<br>0.11	1.376<br>0.18	0.423<br>0.06	2.965<br>1.06	0.573<br>0.06	0.166<br>0.05	2.730<br>0.33	0.934<br>0.07	0.381<br>0.04	4.245<br>0.38	0.794<br>0.09	0.369<br>0.02	1.846<br>0.50	3.259<br>0.82	0.597<br>0.15	> 10.0<br>n.d.
Kyse-70	0.477<br>0.11	0.264<br>0.06	0.532<br>0.12	1.582<br>0.39	0.409<br>0.08	2.095<br>0.86	0.824<br>0.17	0.142<br>0.05	2.289<br>0.25	0.976<br>0.17	0.284<br>0.04	3.602<br>1.07	0.826<br>0.25	0.181<br>0.05	1.516<br>0.32	1.950<br>0.57	0.481<br>0.08	> 10.0<br>n.d.
RT-4	1.926<br>1.10	0.373<br>0.04	0.522<br>0.02	1.595<br>0.43	1.515<br>0.47	5.900<br>1.64	0.878<br>0.09	0.193<br>0.04	2.314<br>0.52	1.306<br>0.34	0.445<br>0.11	2.223<br>1.07	0.716<br>0.19	0.358<br>0.16	1.738<br>0.48	1.548<br>0.46	0.835<br>0.36	5.050<br>0.19
RT-112	0.399<br>0.07	0.465<br>0.10	0.306<br>0.04	1.341<br>0.70	0.559<br>0.08	1.716<br>0.05	1.047<br>0.14	0.246<br>0.04	1.471<br>0.17	1.197<br>0.06	0.469<br>0.06	1.782<br>0.25	1.079<br>0.15	0.384<br>0.02	1.121<br>0.18	3.408<br>0.64	1.104<br>0.12	> 10.0<br>n.d.
A-427	0.349<br>0.06	0.306<br>0.01	0.285<br>0.04	1.461<br>0.19	0.401<br>0.15	1.075<br>0.41	0.785<br>0.07	0.142<br>0.01	0.959<br>0.22	1.094<br>0.1	0.310<br>0	1.189<br>0.11	0.906<br>0.06	0.320<br>0.01	0.834<br>0.15	1.788<br>0.25	0.352<br>0.34	1.391<br>0.29
LCLC-<br>103H	1.416<br>0.17	0.441<br>0.12	0.552<br>0.29	1.357<br>0.61	0.552<br>0.20	1.54<br>0.82	0.571<br>0.17	0.128<br>0.03	0.820<br>0.04	0.674<br>0.05	0.306<br>0.08	0.827<br>0.21	0.800<br>0.29	0.232<br>0.08	0.677<br>0.06	2.816<br>1.29	0.646<br>0.37	2.532<br>0.22
DanG	1.114<br>0.49	0.340<br>0.08	0.478<br>0.09	1.990<br>0.44	0.644<br>0.11	4.354<br>0.72	1.447<br>0.21	0.382<br>0.09	2.660<br>0.36	1.648<br>0.28	0.459<br>0.03	4.460<br>0.43	1.177<br>0.21	0.398<br>0.03	1.620<br>0.22	2.179<br>0.27	0.711<br>0.16	> 10.0<br>n.d.
PATU-<br>8902	0.579<br>0.23	0.480<br>0.02	0.543<br>0.19	1.715<br>0.60	0.659<br>0.28	3.151<br>0.66	1.047<br>0.11	0.257<br>0.04	2.341<br>0.33	0.823<br>0.29	0.471<br>0.03	1.273<br>0.21	1.203<br>0.04	0.340<br>0.05	1.555<br>0.30	2.979<br>1.39	0.952<br>0.09	> 10.0<br>n.d.
YAPC	0.780<br>0.09	0.405<br>0.13	0.360<br>0.14	1.551<br>0.17	0.628<br>0.05	2.234<br>0.27	1.225<br>0.21	0.350<br>0.17	2.367<br>0.61	1.150<br>0.26	0.608<br>0.22	2.213<br>0.14	1.360<br>0.09	0.411<br>0.19	1.518<br>0.26	5.166<br>0.3	1.170<br>0.63	3.721<br>1.19
MCF-7	> 10.0<br>n.d.	0.209<br>0.01	0.970<br>0.12	3.040<br>0.45	0.384<br>0.07	> 10.0<br>n.d.	1.681<br>0.27	0.153<br>0.05	3.561<br>0.75	3.210<br>0.47	0.293<br>0.05	3.561<br>1.07	1.640<br>0.06	0.248<br>0.08	3.011<br>1.28	3.986<br>1.37	0.458<br>0.09	> 10.0<br>n.d.
Mean	0.716	0.333	0.487	1.381	0.493	2.193	0.789	0.174	1.715	1.015	0.322	2.053	0.839	0.267	1.289	2.356	0.671	2.460

IC_{90} values of the pentathiepins as applied in the biological assays

Table S4: IC_{90} values in µM of pentathiepins **1-6** calculated via GraphPad QuickCalcs based on the IC_{50} from MTT (Supplementary table S3) and slope factors of the dose-response curves listed for the relevant cell lines of the further biological evaluations.

	**1**	**2**	**3**	**4**	**5**	**6**
HAP-1	0.71	1.18	0.62	0.51	0.49	1.46
HAP-1.KO.GPx1	0.55	1.20	0.80	0.64	0.72	1.46
A2780	1.87	1.00	0.36	0.61	0.51	2.39
Siso	0.77	2.70	1.23	1.93	1.47	5.07
DanG	1.71	3.06	2.54	2.71	2.05	4.94
PATU-8902	1.22	6.52	2.48	6.00	2.57	7.58
YAPC	2.47	4.26	2.24	3.70	2.20	17.90

Representative histograms from flow cytometric cell cycle analysis

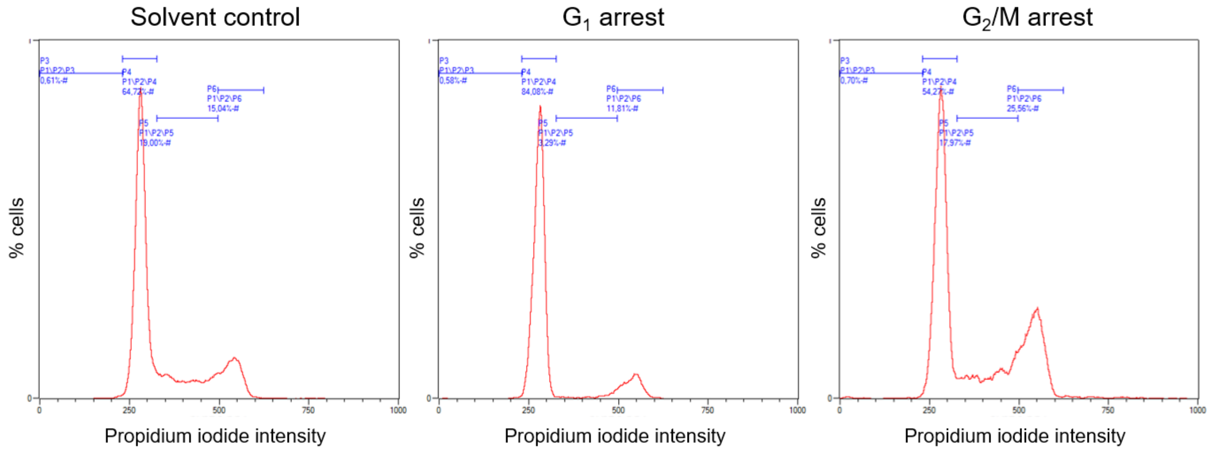

Figure S46: Representative histograms of cell cycle analysis including a reference phase distribution (solvent control, left), a G_1 arrest (middle) and a G_2/M arrest (right).

Representative dot plots from flow cytometric analysis of apoptosis

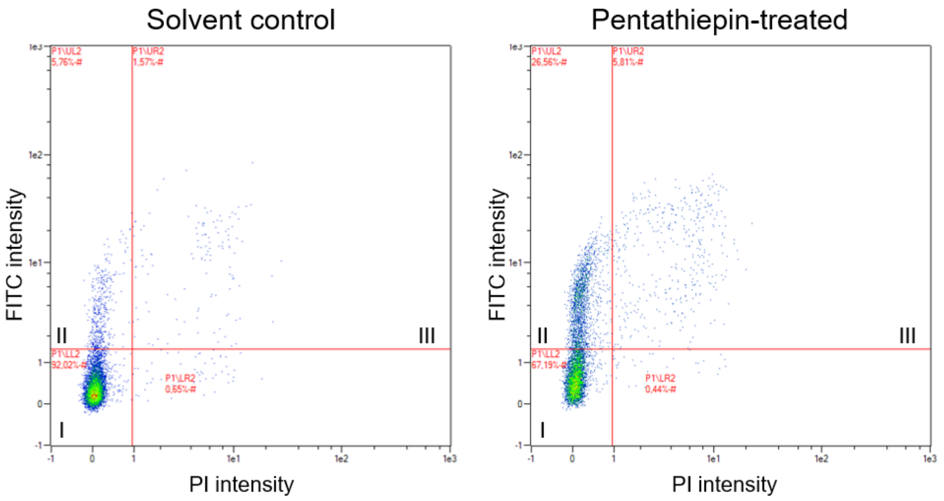

Figure S47: Representative dot plot of flow cytometric analysis of apoptosis using the Annexin V-FITC/PI assay. Quadrant I contains a double negative cell population ($FITC^-/PI^-$) corresponding to viable cells, quadrant II cells positive for Annexin V ($FITC^+/PI^-$) representing early apoptotic cells and quadrant III double positive cells ($FITC^+/PI^+$) that resemble late apoptotic/necrotic cells.

Representative images of comet formation in the Comet assay

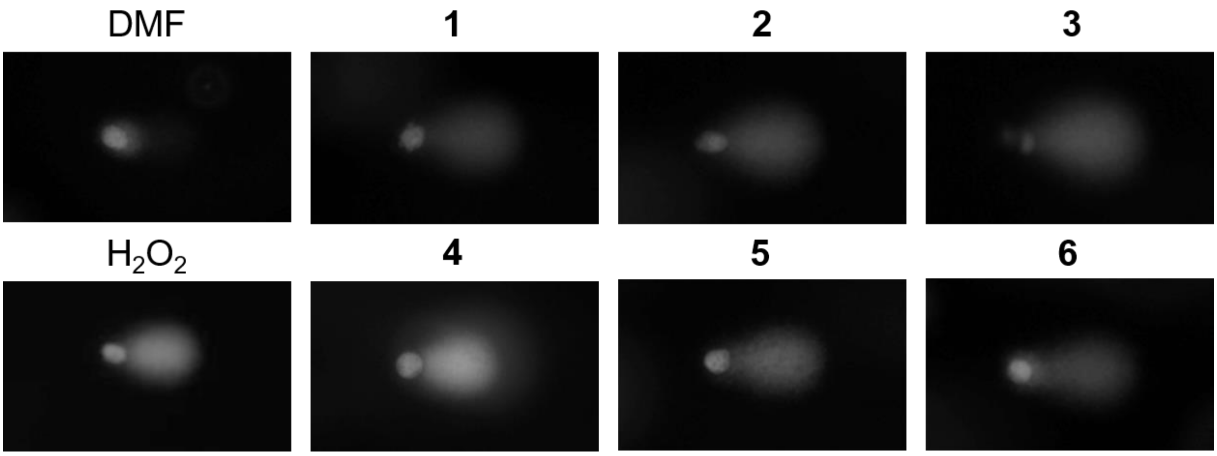

Figure S48: Representative images from comet assay analysis. Here, Siso cells were incubated with pentathiepins **1**-**6** (25 µM). DMF was used as negative control and H_2O_2 as positive control.

Cellular doubling times and Pearson correlation analysis

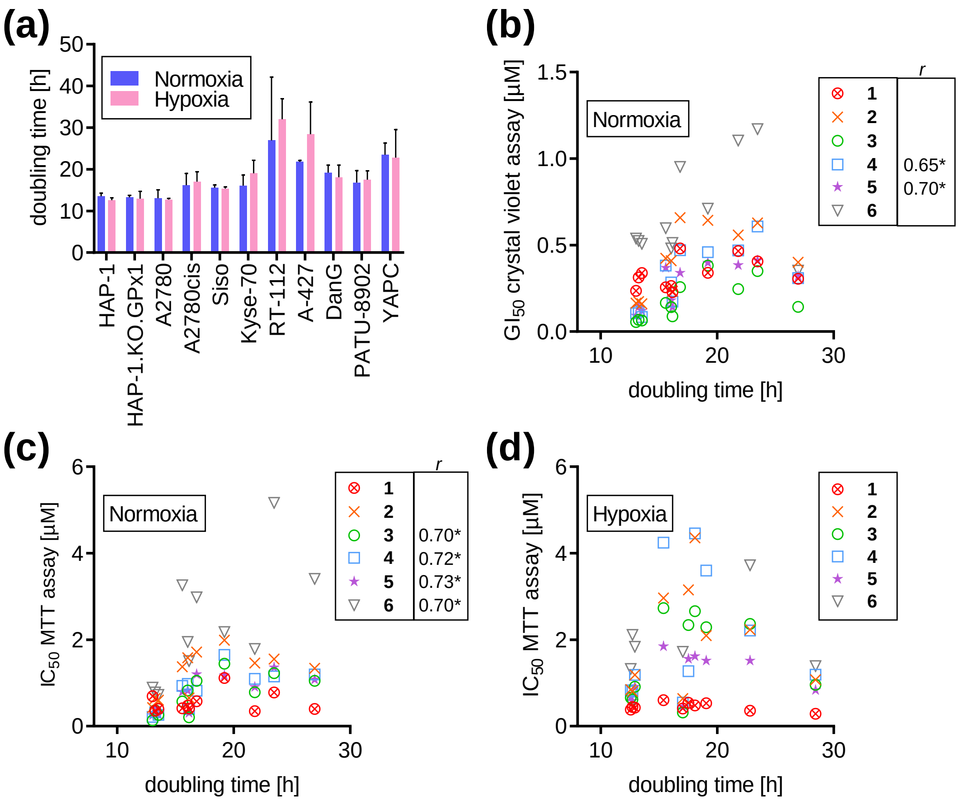

Figure S49. The influence of the cellular doubling time on the cytotoxicity of the pentathiepins **1**-**6**. (a) The doubling times of a panel of 11 human cancer cell lines under normoxic and hypoxic conditions determined with the crystal violet assay over the period of 24, 48, 72 and 96 h in n=3 independent experiments. Hypoxic conditions correspond to an amount of 1 % atmospheric oxygen in the incubator, normoxic conditions to 19 %. (b) A Pearson correlation analysis of the doubling times and the corresponding GI_{50} values from crystal violet assay with corresponding Pearson's r values and significance levels. Pearson correlation analyses of doubling times and the IC_{50} values from MTT under both normoxic (c) and hypoxic (d) conditions with corresponding Pearson's *r* values and significance levels. All correlations were calculated with Prism 7. n≥3 independent experiments. $^*p<0.05$

Testing for ferroptosis

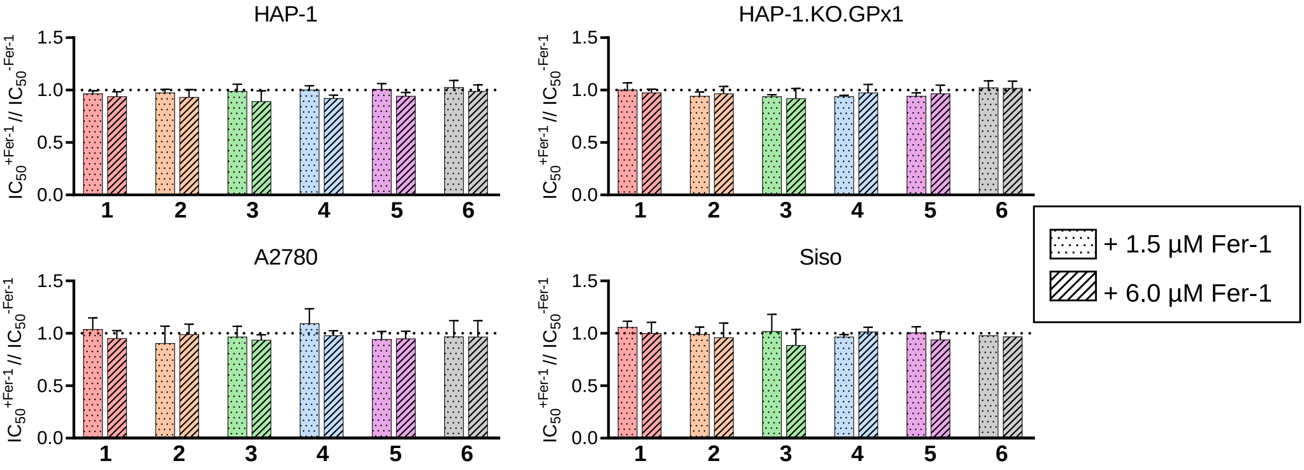

Figure S50: Relative IC_{50} in the four human cancer cell lines HAP-1, HAP-1.KO.GPx1, A2780 and Siso after treatment with pentathiepins **1**-**6** with either vehicle control (DMSO) or 1.5 or 6.0 µM ferrostatin-1 (Fer-1), respectively, for 48 h. The IC_{50} of Fer-1-treated cells were related to DMSO-treated samples (set to 1, dashed line) and data displayed as means and SD from n=3 independent experiments.

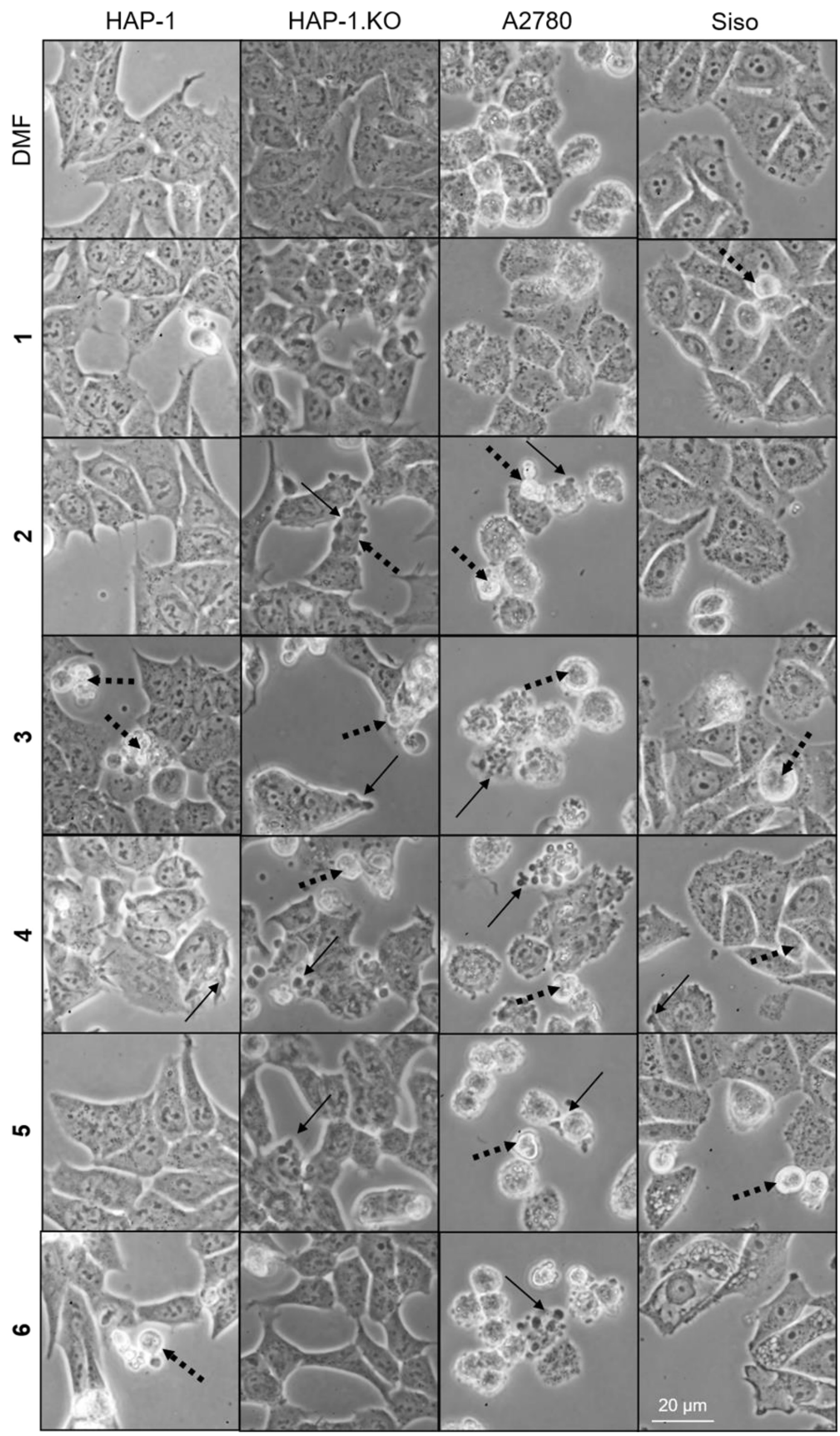

Figure S51: Representative images of cellular morphology after an incubation for 24 h with the respective pentathiepins **1**-**6** (IC_{90}) or a corresponding volume of DMF as negative control in HAP-1, HAP-1.KO.GPx1, Siso and A2780 cells. Arrows denote membrane blebbing, dashed arrows label cell shrinkage.
